# Enzyme-Mediated Dynamic Combinatorial Chemistry Enables Large-Scale Synthesis of δ-Cyclodextrin

**DOI:** 10.1021/jacs.5c02055

**Published:** 2025-04-09

**Authors:** Kasper H. Hansen, Andreas Erichsen, Dennis Larsen, Sophie R. Beeren

**Affiliations:** Department of Chemistry, Technical University of Denmark, Kemitorvet Building 207, Kongens Lyngby 2800, Denmark

## Abstract

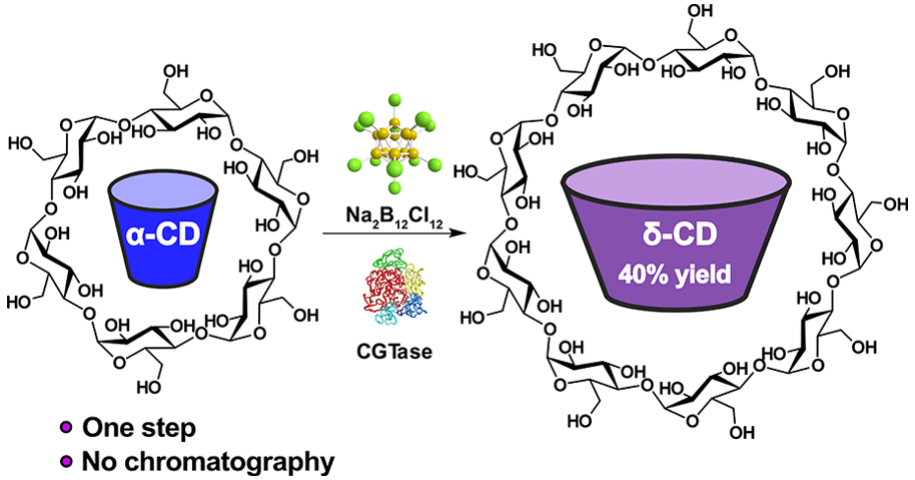

α-, β-, and γ-cyclodextrins (CDs) are macrocycles formed from six, seven, and eight α-1,4-linked d-glucopyranose units and are industrially produced on ton scales for use as hosts for bioactive guests in foods, cosmetics, and pharmaceuticals. Large-ring cyclodextrins, with more than eight glucose units, have been known for decades but never isolated in more than milligram quantities. We report a scalable method to synthesize δ-CD, formed from nine glucose units, in high yield (>40%), high purity (>95% purity without chromatography), and unprecedented quantities (multigram scale). We exploit a superchaotropic dodecaborate template, B_12_Cl_12_^2–^, to direct the selective synthesis of δ-CD from within an enzyme-mediated dynamic combinatorial library of interconverting cyclodextrins. Our single-step reaction uses a recyclable template, cheap starting materials, and a commercial ‘food-grade’ enzyme and can thus give access to large quantities of δ-CD. This work will enable the first large-scale investigations of the properties and applications of this little-known larger CD.

## Introduction

**Figure 1:**
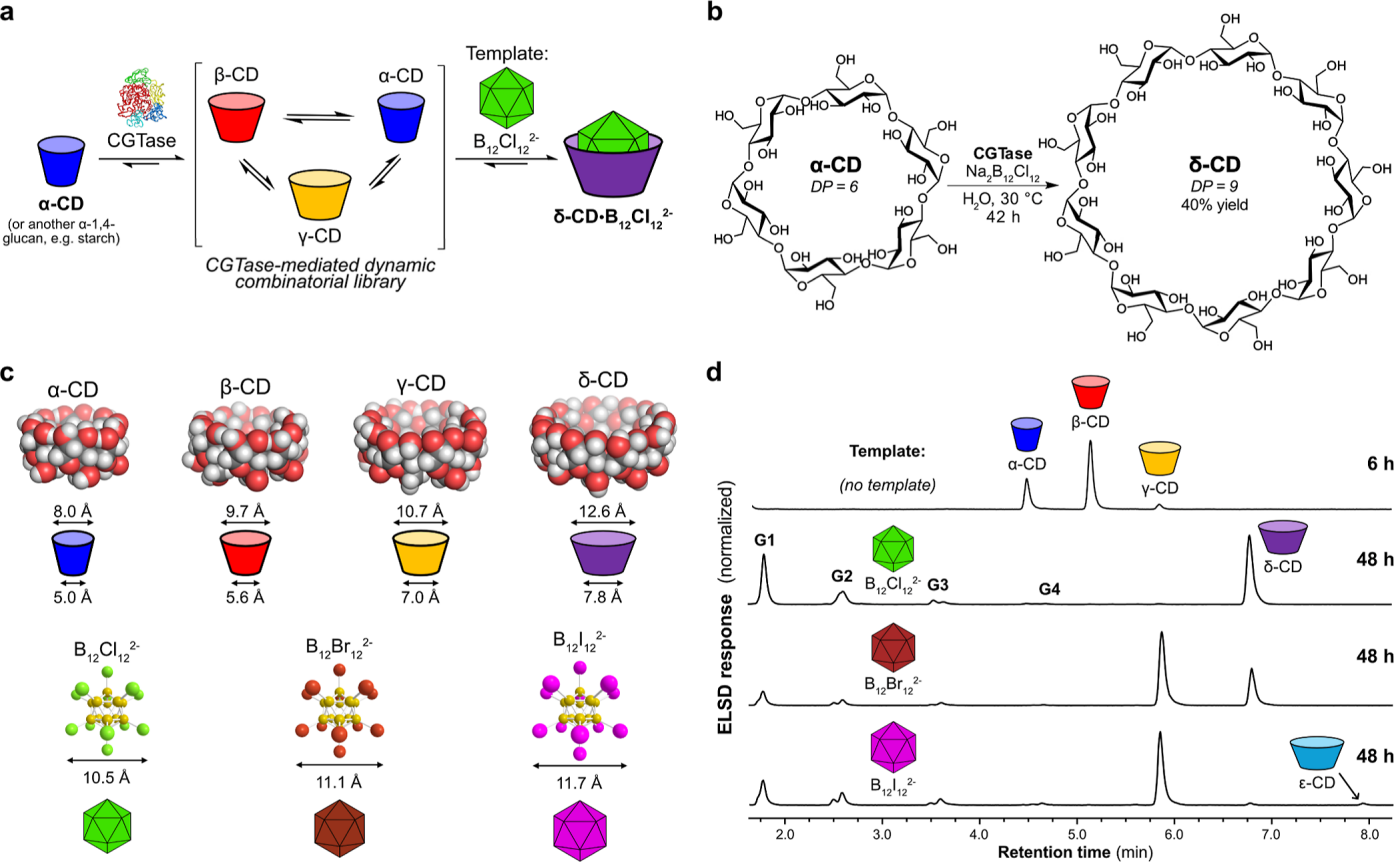
δ-CD synthesis from an enzyme-mediated dynamic combinatorial library (DCL). (a) Schematic showing the DCL achieved by the action of CGTase on α-CD (or any other α-1,4-glucan source) and the effect of using B_12_Cl_12_^2–^ as a template. (b) Reaction scheme for the synthesis of δ-CD from α-CD. (c) Structures of α-, β-, γ-, and δ-CD in space-filling models based on reported X-ray crystallography,^37,52−54^ along with truncated cone illustrations that represent the shape and size of the interior surface of the CDs,^48^ as well as idealized icosahedral structures of the dodecaborate anions. (d) Chromatograms (HPLC-ELSD) showing the product distribution obtained at equilibrium in the reaction of α-CD (10 mg/mL) with CGTase in the absence and presence of Na_2_B_12_Cl_12_, Na_2_B_12_Br_12_, or Na_2_B_12_I_12_ (5 mM). **G1** = d-glucose, **G2** = maltose, **G3** = maltotriose, etc.

Cyclodextrins (CDs), or more specifically, α-, β-, and γ-CD, are the most extensively studied and widely utilized macrocyclic host molecules available.^1−3^ These cyclic oligosaccharides, formed from six, seven, and eight α-1,4-linked d-glucopyranose units, were discovered more than a century ago, and their structures, properties, and derivatization have been rigorously studied throughout the years, especially since the 1970s when their industrial ton-scale production became possible.^1−3^ These three CDs, and their artificially modified analogues, bind hydrophobic and chaotropic guests,^4,5^ which has made them ubiquitous hosts in the field of supramolecular chemistry. Moreover, they serve to encapsulate, solubilize, stabilize, and deliver bioactive guests for a wide array of applications in the pharmaceutical, food, and cosmetics industries.^6−8^ Amid vast scientific and industrial advances, the basic CD toolbox, consisting of α-, β-, and γ-CD, has remained unchanged. Large-ring CDs, formed from more than eight glucose units, were discovered many years ago,^9−12^ but until now, no scalable methods to produce larger CDs have been developed. The organic synthesis of δ-CD, formed from nine glucose units, has been achieved in a 26-step synthesis starting from maltotriose,^13^ and smaller CDs with 3–5 glucose units have also been accessed using elaborate organic synthesis.^14,15^ In this work, we present the first multigram synthesis of a large-ring CD, namely, δ-CD or cyclomaltononaose (Figure 1).

The industrial production of α, β, and γ-CD is carried out via the enzymatic breakdown of starch by cyclodextrin glucanotransferase (CGTase, EC 2.4.1.19).^16,17^ While α, β, and γ-CD are the major products of this reaction, small amounts of large-ring CDs are also formed, mainly in the early stages of the reaction.^18^ Large-ring CDs with 9–31 glucose units have been isolated from complex CD mixtures by chromatography,^19−24^ and recently, the Zimmermann group achieved increased selectivity for large-ring CD production using engineered CGTases.^25,26^ CGTase catalyzes both fast, reversible transglycosylation and slow hydrolysis of α-1,4-glucosidic linkages.^16,27^

We have previously shown how the action of CGTase on an α-1,4-glucan generates a dynamic mixture of interconverting CDs and linear α-1,4-glucans—a dynamic combinatorial library (DCL).^28^ The CDs form a kinetically trapped subsystem that operates under *pseudo*-thermodynamic control wherein the product distribution reflects the relative stabilities of the CDs, and this distribution can be altered by the addition of templates that bind to and stabilize specific CDs.^28−36^ We have recently utilized this system with a bolaamphiphile template to achieve the enzymatic synthesis of δ-CD in a 7% yield, where δ-CD was isolated from a biased mixture of CDs using preparative HPLC.^33^

While accumulated knowledge of the binding properties of δ-CD is still quite limited, a handful of studies have shown that δ-CD can form weak inclusion complexes with cycloundecanone,^37^ I_3_^–^, sodium dodecasulfate,^38^ some steroidal compounds,^20^ and some hydrophobic drugs.^39^ It can help to solubilize fullerenes,^40−43^ discriminate between enantiomers of small racemic drugs,^44−46^ and form [2]-, [3]-, and [4]-*pseudo*rotaxanes with bolaamphiphiles with alkyl chain axles.^33^ Of particular relevance to this work, Nau and co-workers have discovered the extremely high-affinity binding of superchaotropic halogenated dodecaborate cluster dianions, B_12_Cl_12_^2–^, B_12_Br_12_^2–^, and B_12_I_12_^2–^, to γ-, δ-, and ε-CD, formed from 8, 9, and 10 glucose units, respectively.^47,48^ The strong binding of polyoxometallates to γ-CD has also been attributed to the chaotropic effect.^49−51^

Herein, we present a scalable, dynamic enzymatic synthesis of δ-CD that employs Na_2_B_12_Cl_12_ as a recyclable template to amplify δ-CD from within a dynamic mixture of interconverting CDs (Figure 1a,b). α-CD can be converted to δ-CD in 40% yield in a single reaction step to obtain a high-purity product (>95% purity) without the need for chromatography. Detailed binding studies using NMR spectroscopy and isothermal titration calorimetry (ITC) show how γ-, δ-, and ε-CD form various 1:1 and 2:1 complexes with the halogenated dodecaborate cluster dianions. Simulations of the DCL product distributions reveal how the delicate interplay between relative binding strengths, binding stoichiometry, and the relative stabilities of the free cyclodextrins can explain the extremely selective templating effect achieved using B_12_Cl_12_^2–^.

## Results and Discussion

### Enzyme-Mediated DCLs of CDs with Halogenated Dodecaborate Templates

A series of enzyme-mediated DCLs were set up to test the ability of dodecaborate anions to act as templates for the enzymatic synthesis of large-ring CDs. α-CD (10 mg/mL) was treated with CGTase in the presence of Na_2_B_12_Cl_12_, Na_2_B_12_Br_12_, or Na_2_B_12_I_12_ (5 mM) in phosphate-buffered water (50 mM, pH 7.5) at room temperature. The distribution of products formed in these DCLs was monitored over time using HPLC with evaporative light scattering detection (ELSD) (Figures S11, S13, and S14). Figure 1d displays chromatograms showing the distributions of α-1,4-glucan products formed once equilibrium had been reached (48 h for the templated libraries and 6 h for the untemplated library). In the untemplated library, α-CD, β-CD, and γ-CD were formed as the main products, and only trace amounts of large-ring CDs were detected. In the presence of Na_2_B_12_Cl_12_, remarkably selective production of δ-CD occurred such that δ-CD accounted for 84% of the CD composition. Some short linear glucans and glucose were also formed, which is due to an unavoidable background hydrolysis reaction.^28^ In both the DCLs templated with Na_2_B_12_Br_12_ and Na_2_B_12_I_12_, the dominant product was γ-CD, but a significant proportion of δ-CD (35%) was formed in the presence of Na_2_B_12_Br_12_ and small amounts of both δ-CD (7%) and ε-CD (9%) were formed in the library templated with Na_2_B_12_I_12_.

The highly selective conversion of α-CD to δ-CD seen in the Na_2_B_12_Cl_12_-templated DCL is comparable with some of the best template-directed macrocycle syntheses reported to date.^55,56^ It is especially remarkable, given the structural similarities between the different-sized CDs and the reported high affinity of each dodecaborate anion for several different CDs.^47,48^ In order to fully understand these templating effects, therefore, we decided to carry out a thorough investigation of the binding interactions of Na_2_B_12_Cl_12_, Na_2_B_12_Br_12_, and Na_2_B_12_I_12_ with γ-CD, δ-CD, and ε-CD, using a combination of ^1^H NMR spectroscopy and ITC. For this purpose, δ-CD and ε-CD were isolated from preparative-scale DCLs templated with Na_2_B_12_Cl_12_ and Na_2_B_12_I_12_, respectively, using preparative HPLC with a hydrophilic interaction liquid chromatography (HILIC) column.

### Investigation of the Binding Interactions between CDs and Dodecaborate Anions

**Figure 2:**
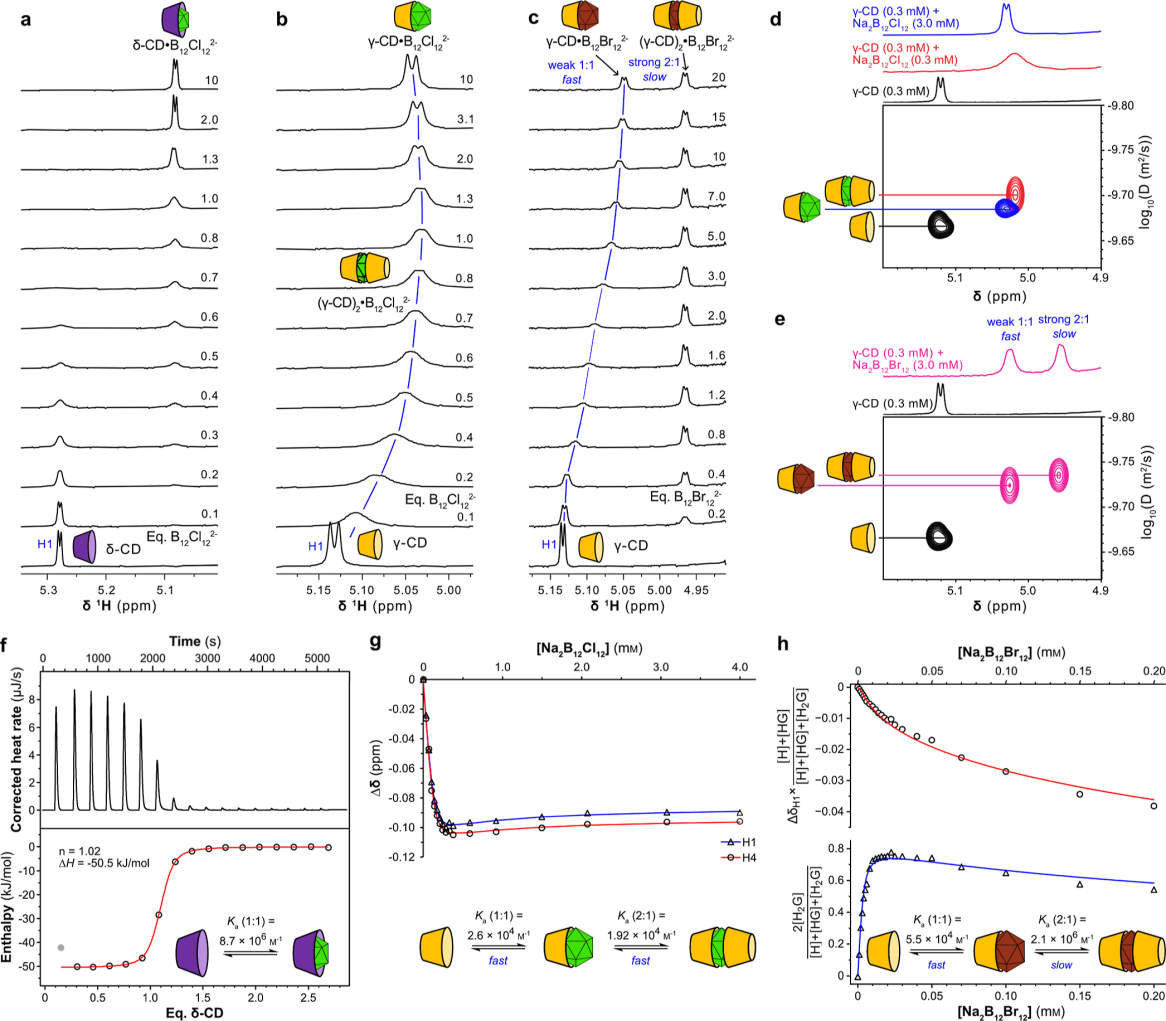
Binding studies with CDs and dodecaborates. (a–c) Partial ^1^H NMR spectra showing the anomeric region from the titrations of δ-CD (0.01 mM) with Na_2_B_12_Cl_12_, γ-CD (0.3 mM) with Na_2_B_12_Cl_12_, and γ-CD (0.01 mM) with Na_2_B_12_Br_12_ in D_2_O, respectively. (d,e) Partial DOSY NMR spectra showing the anomeric region of γ-CD alone and in the presence of Na_2_B_12_Cl_12_ and Na_2_B_12_Br_12_, respectively. (f) Baseline-corrected heat rate and fit obtained for the ITC titration of δ-CD with Na_2_B_12_Cl_12_ in H_2_O. (g) Fit to the changes in chemical shift (Δδ) during the titration of γ-CD with Na_2_B_12_Cl_12_. (h) Fit obtained in the titration of γ-CD with Na_2_B_12_Br_12_.

^1^H NMR titrations were performed by titrating the dodecaborate salts Na_2_B_12_X_12_ (X = Cl, Br, or I) into D_2_O solutions of γ-CD, δ-CD, and ε-CD. Previous work has shown that α- and β-CD only exhibit weak nonspecific interactions to these dodecaborate salts,^47,48^ which was confirmed for B_12_Cl_12_^2–^ (Figures S18–S21). In the titrations of δ-CD with each of the dodecaborate salts, binding was observed in slow exchange on the NMR chemical shift timescale (Figures 2a and S28–S33). Strong binding interactions with 1:1 stoichiometry were clearly indicated with affinities higher than could be determined using ^1^H NMR titrations (i.e., above ca. 10^6^m^–1^), and so, the association constants (*K*_a_) were determined using ITC (Figures 2f and S45–S47). Consistent with previous reports,^48^ the highest affinity for δ-CD was found for B_12_Br_12_^2–^ (*K*_a_ = 1.2 × 10^7^ M^–1^), followed by B_12_Cl_12_^2–^ (*K*_a_ = 8.7 × 10^6^ M^–1^) and B_12_I_12_^2–^ (*K*_a_ = 9.2 × 10^5^ M^–1^) (Table 1). In the NMR titrations of ε-CD with Na_2_B_12_Cl_12_ and Na_2_B_12_Br_12_, binding was observed in a fast exchange regime, and moderate to strong 1:1 binding was determined (*K*_a_ = 2.7 × 10^4^ M^–1^ and *K*_a_ = 2.5 × 10^5^ M^–1^, respectively) (Figures S34–37). Very strong binding of ε-CD to Na_2_B_12_I_12_ was seen in slow exchange, so again we turned to ITC to determine the 1:1 association constant (*K*_a_ = 3.4 × 10^6^ M^–1^) (Figures S38, S39, and S48).

**Table 1:** Association Constants (*K*_a_) for Binding of Dodecaborate Anions to γ-CD, δ-CD, or ε-CD^a^

	*K*_a_ (M^–1^) for dodecaborate guests^b^			
Host		B_12_Cl_12_^2–^	B_12_Br_12_^2–^	B_12_I_12_^2–^
γ-CD	*K*_a_ (1:1)	2.6 × 10^4^	5.5 × 10^4^	2.4 × 10^5^
	*K*_a_ (2:1)	1.9 × 10^4^	2.1 × 10^6^	2.1 × 10^4^
δ-CD^c^	*K*_a_ (1:1)	8.7 × 10^6^	1.2 × 10^7^	9.2 × 10^5^
ε-CD	*K*_a_ (1:1)	2.7 × 10^4^	2.3 × 10^5^	3.4 × 10^6^^c^

a Determined by ^1^H NMR spectroscopy titration in D_2_O, unless stated otherwise. Uncertainties on the fits are given in Section S9.

b All as their disodium salts.

c Determined by ITC in H_2_O.

The ^1^H NMR titrations with γ-CD showed greater complexity and revealed a hitherto undetected^47^ 2:1 binding stoichiometry in the solution phase ((γ-CD)_2_·B_12_X_12_^2–^). In the titrations with Na_2_B_12_Cl_12_ and Na_2_B_12_I_12_, binding was observed in fast exchange and the presence of complexes with two different stoichiometries was clearly indicated by the changing direction of the chemical shift perturbations upon addition of dodecaborate anions (Figures 2b, S22, S23, S26, and S27). Job plots likewise indicated 2:1 binding interactions, and in both cases, the chemical shift changes fit well to a 2:1 binding model (Figure 2g). For B_12_Cl_12_^2^, moderate 1:1 and 2:1 binding constants were measured (*K*_a(1:1)_ = 2.6 × 10^4^ M^–1^, *K*_a(2:1)_ = 1.9 × 10^4^ M^–1^), while for B_12_I_12_^2–^, the first binding was an order of magnitude higher (*K*_a(1:1)_ = 2.4 × 10^5^ M^–1^, *K*_a(2:1)_ = 2.1 × 10^4^ M^–1^). DOSY NMR experiments were performed on solutions of γ-CD and Na_2_B_12_Cl_12_ at varying ratios, showing that with substoichiometric equivalents of Na_2_B_12_Cl_12_, the observed diffusion coefficient was smaller than with higher equivalents of Na_2_B_12_Cl_12_, which is consistent with the formation of the (γ-CD)_2_·B_12_Cl_12_^2–^ and γ-CD·B_12_Cl_12_^2–^ complexes in fast exchange (Figures 2d and S41).

In the titration with Na_2_B_12_Br_12_, we observed an unusual situation, where the formation of the 1:1 complex was seen in fast exchange, while the 2:1 complex was formed in slow exchange (Figures 2c and S24 and S25). DOSY NMR experiments showed clearly the formation of two discrete complexes, γ-CD·B_12_Br_12_^2–^ and (γ-CD)_2_·B_12_Br_12_^2–^ with different diffusion coefficients (Figures 2e and S42). By monitoring both the chemical shift changes and the integrals of each signal (and building upon methodology we have developed previously to handle a similar situation albeit with 1:1 and 1:2 binding in fast and slow exchange),^33^ we could determine association constants, *K*_a(1:1)_ = 5.5 × 10^4^ M^–1^ and *K*_a(2:1)_ = 2.1 × 10^6^ M^–1^ (Figure 2h and Section S11). The strong and highly cooperative 2:1 binding of γ-CD to B_12_Br_12_^2–^ suggests that two γ-CD molecules can optimally encapsulate and thereby desolvate one molecule of B_12_Br_12_^2–^, alleviating the superchaotropic effect the dodecaborate ion exerts on the bulk water.^57^ This solution-state binding stoichiometry is consistent with the reported crystal structure for this complex.^47^ It is noteworthy that for the complexation of γ-CD with all three dodecaborates, large downfield shifts of the H3 but not the H5 proton signals were observed (Figure S40). This is indicative of shallow binding where only the protons inside the CD cavity close to the wide rim are perturbed and consistent with the formation of a 2:1 sandwich complex ((γ-CD)_2_·B_12_X_12_^2–^).^58^ In contrast, for the complexation of δ-CD with B_12_Cl_12_^2–^ and B_12_Br_12_^2–^, large downfield shifts for the H5 protons, which are located near the narrow rim of the CD, were observed, indicating that the guest penetrates deeply into the cavity, as would be expected for a strong 1:1 complex.

### DCL Simulations

**Figure 3:**
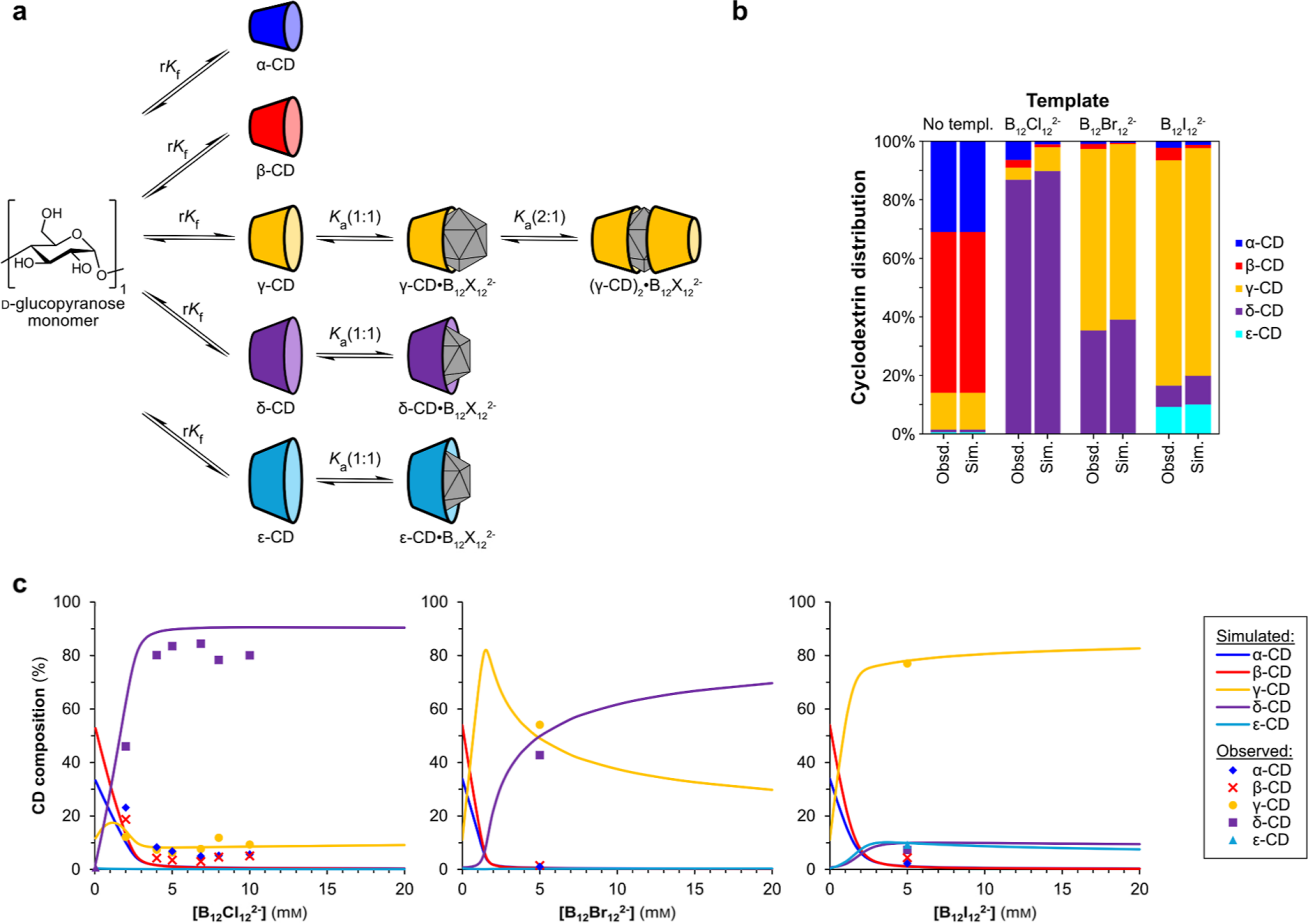
(a) DCLSim model used to simulate the product distribution in DCLs templated with dodecaborates. (b) Comparison between observed CD distributions at 48 h (Obsd.) and the simulated values (Sim.) at the same total CD concentration for the untemplated DCL and DCLs templated with 5 mM Na_2_B_12_Cl_12_, Na_2_B_12_Br_12_, or Na_2_B_12_I_12_. (c) DCLSim predictions of CD distribution (lines) and observed CD distributions (data points) as a function of template concentration, assuming a total CD concentration of 4 mg/mL.

We were surprised to find that although B_12_Cl_12_^2–^ appeared to be the most efficient template for the enzymatic synthesis of δ-CD, B_12_Br_12_^2–^ had the highest affinity for δ-CD. However, DCLs are complex networks of interconnected equilibria and can exhibit counterintuitive behavior.^59^ The product distribution reached at equilibrium is representative of the lowest energy of the entire system and, thus, is influenced not only by the strength of the individual binding interactions but also by the stoichiometry of the binding interactions, the number of building blocks (glucose units) required to assemble each library member, and the intrinsic stability of the different library members. To rationalize the observed product distributions, therefore, we used the DCLSim software developed in the Otto and Sanders groups to simulate DCLs of CDs according to the model shown in Figure 3a.^59^ We utilized our measured binding constants and determined relative formation constants for α-, β-, γ-, δ-, and ε-CD from the product distribution in an untemplated library ( Section S13). The DCLs were simulated at a total CD concentration of 4 mg/mL as it takes up to 48 h for the DCLs templated with dodecaborate anions to reach equilibrium, and by this time, approximately 60% of the CDs have been hydrolyzed to form **G1**–**G4**. Previous work has shown that *pseudo*-thermodynamic control operates for the CD subsystem, and so the linear glucans can be disregarded in the simulation.^28^Figure 3b shows a comparison between the simulated and experimental product distributions obtained for DCLs templated with 5 mM Na_2_B_12_Cl_12_, Na_2_B_12_Br_12_, and Na_2_B_12_I_12_.

A very good correlation between our experimentally determined yields of γ-, δ-, and ε-CD and the simulated results was found, which confirms the validity of the binding constants and stoichiometries that we determined. Compared to the simulations, marginally higher concentrations of α-CD and β-CD were obtained in the experiments, but this can be explained by the fact that the system is not truly at equilibrium. Short linear glucans are constantly exiting and re-entering the kinetically trapped CD subsystem, and there is a kinetic preference for them to form smaller CDs before eventual conversion to the template-stabilized larger CDs.^28,36^

Simulated DCLs were generated with varying concentrations of each template, and the predicted distributions of α-CD−ε-CD are plotted in Figure 3c (lines). In the DCLs templated with B_12_Br_12_^2–^ and B_12_Cl_12_^2–^, we found that there exists a competition between the formation of 1:1 complexes with δ-CD or 2:1 complexes with γ-CD. For B_12_Br_12_^2–^, the 2:1 complex with γ-CD is highly stable, and this results in the production of similar quantities of γ-CD and δ-CD when a 5 mM template concentration is employed. Simulations predict that with fewer equivalents of the template, γ-CD production will be favored, while increasing the template concentration favors δ-CD, albeit never reaching the yields of δ-CD obtained with B_12_Cl_12_^2–^ (Figure S54). For DCLs templated with B_12_Cl_12_^2–^, the highly selective production of δ-CD can be explained by the fact that B_12_Cl_12_^2–^ forms a much weaker 2:1 complex with γ-CD. The simulation indicated that 5 mM Na_2_B_12_Cl_12_ would be sufficient to give a high δ-CD yield. This result was confirmed experimentally by setting up a series of DCLs with different concentrations of the Na_2_B_12_Cl_12_ template. A sharp decrease in δ-CD yield was seen below 4 mM Na_2_B_12_Cl_12_, and it was observed that higher template concentrations (8–10 mM) caused slower evolution of the libraries and no benefit to the δ-CD yield (Figure 3c (data points) and Figure S12). For DCLs templated with B_12_I_12_^2–^, simulations predicted the formation of mainly γ-CD and only very small quantities of δ-CD and ε-CD even at high template concentrations. In the untemplated DCL, δ-CD and ε-CD make up less than 1% and 0.5% of the total CD concentration, respectively, while γ-CD accounts for 12.5% (Section S13). The product distribution in the untemplated DCL reflects the intrinsic stability of each library member. The concentration of library members in templated DCLs is then a function of their stability relative to one another and their relative affinity for the template. It seems that the affinity of B_12_I_12_^2–^ for these large-ring CDs is insufficiently high to cause their amplification in the DCL due to competitive binding by γ-CD and the much higher intrinsic stability of γ-CD.

### Large-Scale Synthesis of δ-CD

**Figure 4:**
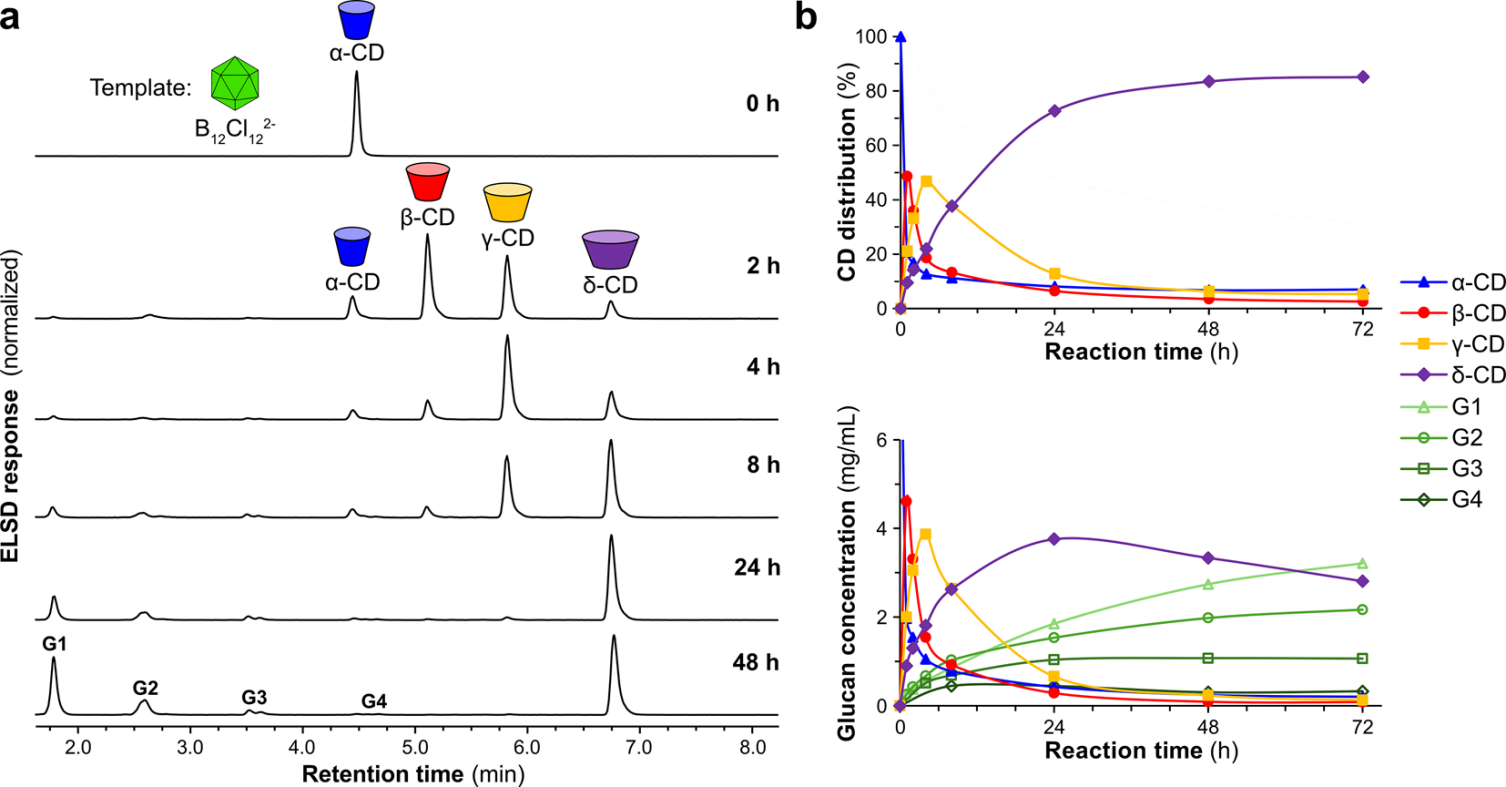
Optimization of δ-CD synthesis. (a) Chromatograms (HPLC-ELSD) showing the evolution of a DCL wherein α-CD was treated with CGTase in the presence of Na_2_B_12_Cl_12_ (5 mM) in sodium phosphate buffer at pH 7.5. **G1** = d-glucose, **G2** = maltose, **G3** = maltotriose, etc. (b) Distribution of glucans formed in the DCL as a function of time: (top) relative concentrations of CDs formed; (bottom) concentrations of all glucans formed. Smoothed lines are added to guide the eye.

Having determined the optimal template concentration for δ-CD production, the next step toward a scalable synthesis was to optimize the reaction time. The evolution of the product distribution in the DCL templated with 5 mM Na_2_B_12_Cl_12_ was monitored closely. Figure 4a displays chromatograms showing the gradual conversion of α-CD to β-CD, γ-CD, and finally δ-CD over 48 h. The changing CD distribution and concentrations of all glucans in the mixture (including linear and cyclic species) are plotted in Figure 4b. The highest selectivity toward δ-CD was observed after 48 h once the system had reached equilibrium and almost all γ-CD had been converted to δ-CD. However, the highest yield of δ-CD was obtained after 24 h as significant hydrolysis occurred during the second day, leading to a buildup of **G1**–**G4** and a lower total CD concentration. Since ease of purification is a critical consideration when developing a scalable synthesis, we judged that a longer reaction time is optimal, despite a small sacrifice in δ-CD yield, as the separation of short linear glucans from δ-CD by precipitation is relatively trivial, while the separation of γ-CD from δ-CD is challenging and requires chromatography.

**Figure 5:**
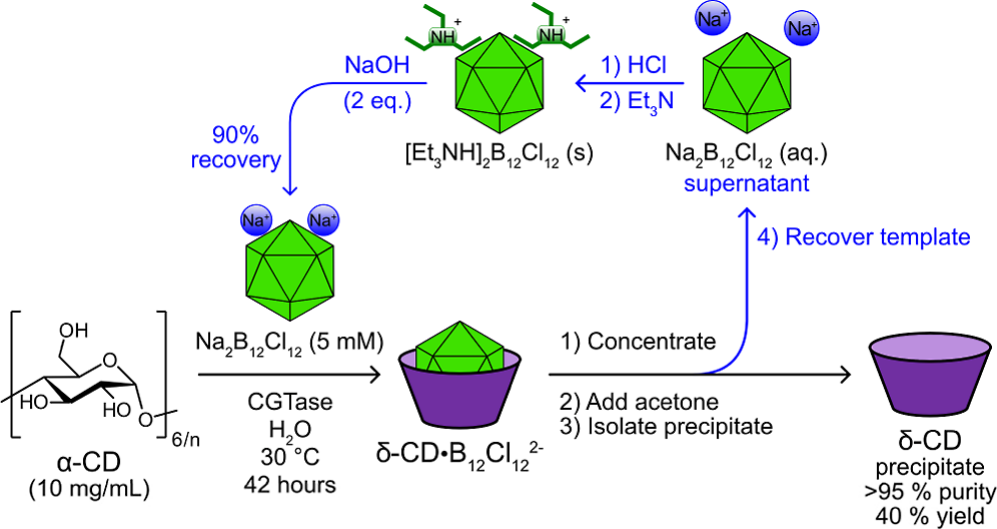
Schematic of the scalable reaction process for δ-CD synthesis from an α-glucan source, including the template recovery process (in blue arrows).

We have thus identified a scalable process for δ-CD production that yields high-purity δ-CD without the use of chromatography and with efficient template recovery (Figure 5).^60^ The reaction is carried out by combining α-CD (10.0 g), Na_2_B_12_Cl_12_ (3.0 g), and a commercial CGTase stock solution in water (1 L) at pH 7.5 and at 30 °C. After 42 h, the reaction mixture is boiled for 15 min to denature the enzyme, concentrated to 20% of the original reaction volume, and then δ-CD is precipitated by addition of acetone. Purification by three reprecipitations gives δ-CD in at least 40% yield with a purity of at least 95%, according to ^1^H NMR spectroscopy (Figure S1). ^11^B NMR spectroscopy is used to confirm the absence of any template in the precipitate. δ-CD with greater than 99% purity can be obtained by further purification using preparative HPLC with a HILIC column (Figure S3). Importantly, the template can be recovered with 90% efficiency and used in subsequent reaction cycles. The filtrate obtained after precipitation of δ-CD is concentrated and acidified with aqueous HCl. Et_3_N is added to precipitate [Et_3_NH]_2_[B_12_Cl_12_], and then NaOH is used for conversion to the water-soluble Na_2_B_12_Cl_12_. The template has been used in at least 3 sequential reaction cycles with no decrease in the efficiency of δ-CD production. Additionally, the purity of the recovered template was confirmed using ^1^H and ^11^B NMR spectroscopy (Figure S7).

To further showcase the utility of Na_2_B_12_Cl_12_ for the enzymatic synthesis of δ-CD, we tested starch as the α-1,4-glucan starting material as starch is the raw material typically used in the industrial synthesis of small CDs and would be a logical starting material for industrial-scale synthesis of δ-CD. Performed at an analytical scale, the reaction proceeded in a similar manner as when starting from α-CD (Figure S15). After 24 h, δ-CD was the main cyclic product. Small quantities of 6-*O*-α-d-glucopyranosyl-δ-CD were also formed, which can be explained by the fact that amylopectin, the main component of starch, has a dendrimer-like structure with long α-1,4-glucan chains connected by α-1,6-linked branching points. This branched CD side product could be avoided by addition of two debranching enzymes, isoamylase and pullulanase, alongside CGTase during the synthesis (Figure S16).

## Conclusions

In summary, we exploited the chaotropic effect in an enzyme-mediated DCL to access unprecedented quantities of a large-ring cyclodextrin. Where δ-CD has previously been obtained in milligram quantities,^13,20,33^ we have synthesized grams of this rare macrocycle using a cheap starting material (α-CD), a ‘food-grade’ enzyme, and a recyclable template. Due to the unusually high selectivity of this template-directed reaction, δ-CD can be isolated in >95% purity and >40% yield by simple precipitation. The template can be recovered by precipitation with high efficiency, and the recovered template has been used successfully for several subsequent δ-CD syntheses on milligram to 10 g scales. Careful analysis of the binding interactions between B_12_Cl_12_^2–^, B_12_Br_12_^2–^, and B_12_I_12_^2–^ and γ-CD, δ-CD, and ε-CD, and simulation of product distributions in DCLs, revealed that the high selectivity of this B_12_Cl_12_^2–^-templated synthesis results as much from the high affinity of B_12_Cl_12_^2–^ for δ-CD as from the lack of a competing strong 2:1 binding to γ-CD. We believe that our methodology could eventually enable the industrial-scale production of δ-CD so that in the future, δ-CD may find its place alongside α-CD, β-CD, and γ-CD, as a versatile host for a wide range of applications.

## Supporting Information Available

Experimental procedures, HPLC reaction monitoring of enzyme-mediated DCLs, NMR titrations, ITC experiments, details of simulations, and characterization data to support the conclusions (PDF)

## Abbreviations

CD: cyclodextrin
CGTase: cyclodextrin glucanotransferase
DCL: dynamic combinatorial library.
