# Supplementary material for: Enzyme-Mediated Dynamic Combinatorial Chemistry Enables Large-Scale Synthesis of δ-Cyclodextrin
Source: J Am Chem Soc. 2025 Apr 9;147(16):13851–8. doi: 10.1021/jacs.5c02055 (PMC12022984; doi:10.1021/jacs.5c02055)
Supplement: Supplementary file 1 — ja5c02055_si_001.pdf [file ja5c02055_si_001.pdf]

Enzyme-Mediated Dynamic Combinatorial Chemistry Enables Large-Scale Synthesis of δ-Cyclodextrin

*Kasper H. Hansen,[†] Andreas Erichsen,[†] Dennis Larsen, Sophie R. Beeren**

[†] *Equal first authors.*

* Corresponding Author: sopbee@kemi.dtu.dk

Technical University of Denmark, Department of Chemistry, Kemitorvet Building 207, Kongens Lyngby 2800, Denmark

Supplementary Information

Contents

S1 Materials 2

S2 Instruments and methods 3

S3 Synthesis of templates 4

S4 Large scale synthesis of δ-CD 6

S5 Characterization of batches of δ-CD 8

S6 Synthesis and isolation of ε-CD (CD10) 16

S7 Synthesis of 6-O-α-D-glucopyranosyl-δ-cyclodextrin (G1-δ-CD) 17

S8 CGTase-mediated Dynamic Combinatorial Libraries (DCLs) at an analytical scale 20

S9 NMR spectroscopy titrations of CDs (α-, β-, γ-, δ-, ε-CD) with $Na_2B_{12}X_{12}$ (X = Cl, Br, I) 28

S10 DOSY NMR experiments 51

S11 Algorithm for determining association constants from data obtained in NMR titrations with 2:1 binding in mixed fast and slow exchange 53

S12 ITC titrations of δ-CD or ε-CD with $Na_2B_{12}X_{12}$ (X = Cl, Br, I) 56

S13 Simulation of library distributions 61

S14 References 69

S1 Materials

All chemicals and solvents were obtained from commercial suppliers and used as received for both synthesis and chromatographic analysis, unless stated otherwise. Solvents were of HPLC (high performance liquid chromatography) grade or better. Soluble starch (product no. S9765) was purchased from commercial supplier Sigma Aldrich. Isoamylase (18500000 U/ml) from *Pseudonomas sp.* (E.C. 3.2.1.68) was purchased from Sigma Aldrich and pullulanase (650 U/ml) from *Krebsiella planticola* (E.C. 3.2.1.41) was purchased from Megazyme.

A stock solution of the enzyme CGTase derived from *Bacillus macerans* was purchased from Amano Enzyme, Inc., Nagoya, Japan and stored at 5 °C. According to specifications from the supplier, the stock solution contains approximately 20% glycerol. Glycerol-free CGTase was obtained by spin filtration as previously described.[1] The commercial stock solution of CGTase and the glycerol-free stock solution of CGTase were used in the enzymatic reactions as indicated for each experiment. In general, the commercial enzyme solution was used for preparative scale syntheses, while the glycerol-free stock solution was used for analytical scale experiments.

High purity water used in both chromatographic analysis and in reactions was obtained using a Merck Millipore Synergy UV water purification system. Colorless microcentrifuge tubes (0.6 ml, low retention, sterile) were used for analytical scale enzymatic reactions and sample preparation (acidification, dilution and centrifugation). Colorless 2 ml glass vials with PTFE-lined (polytetrafluoroethylene) screw-cap septa and 0.2 ml glass inserts were used for short-term sample storage and injection on HPLC equipment. NMR samples were analyzed in capped standard 5 mm borosilicate glass NMR tubes. Colorless spin filter tubes (VIVASPIN TURBO 30K MWCO, 50 ml) were used for spin filtration of enzymatic reaction mixtures.

S2 Instruments and methods

Chromatographic analysis was performed on a Thermo Scientific Dionex Ultimate 3000 HPLC (ultra-high pressure) system equipped with a Waters Acquity Glycan BEH Amide 1.7 µm 2.1 × 150 mm column maintained at 30 °C and an autosampler maintained at 20 °C. Detection was carried out using an Agilent Technologies 1260 Infinity ELSD (evaporative light scattering detector), operating with the evaporator at 90° C, nebulizer at 70 °C and a N_2 gas flow of 1.0 l/min. The gradient profile for HPLC was a linear gradient from 75% acetonitrile in water to 55% acetonitrile in water over 8 min with a flow rate of 0.6 ml/min. Both eluents contained 0.1% formic acid by volume. For quantification from HPLC chromatograms, calibration curves for α, β, γ and δ-CD and short linear α-1,4-glucans glucose (**G1**), maltose (**G2**) and maltotriose (**G3**) were used to correct for differences in the ELS detector response for different oligosaccharides using a previously published method.[2] For linear α-1,4-glucans with more than three glucose units, the calibration parameters for **G3** were used. For 6-O-α-D-glucopyranosyl-δ-CD (G1-δ-CD), the calibration parameters for δ-CD were used.

NMR spectra were acquired on a Bruker Avance III 400 MHz NMR spectrometer equipped with a Prodigy broadband observe (BBO) probe, a Bruker Avance III 400 MHz NMR spectrometer equipped with a BBO Smartprobe, a Bruker Avance III 600 MHz spectrometer or a Bruker Avance III 800 MHz NMR spectrometer equipped with a Bruker TCI Cryoprobe. NMR spectra were referenced to residual solvent peaks and measured at 298 K unless stated otherwise. NMR spectra were processed in the software Topspin 3.6.2 or the software MestreNova 11.0. Chemical shifts (δ) are quoted in ppm and coupling constants (*J*) are quoted in Hz. Data from NMR spectroscopy titrations was analyzed using the supramolecular.org web applet (https://supramolecular.org).[3,4]

ITC titration experiments were performed on a TA Instruments Nano ITC calorimeter equipped with a "standard volume" (SV) cell of 998 µl. The calorimeter was calibrated by titration of a standardized 10.0 mM HCl solution into a 0.04 M tris(hydroxymethyl)aminomethane (TRIS) solution as per the procedure provided by the instrument manufacturer. ITC titration data was analyzed using NanoAnalyze Data Analysis software version 3.15.5 from TA Instruments.

MALDI-TOF-MS experiments were carried out on a Bruker autoflex speed instrument. ESI-MS experiments were performed using an Agilent Infinity II 1290 UHPLC system (Agilent Technologies, Santa Clara, CA, USA) to provide a 0.1 ml/min flow of 1:1 acetonitrile/10 mM ammonium acetate as carrier solvent for injection into an Agilent 6545 QTOF mass spectrometer equipped with an Agilent Dual Jet Stream electrospray ion source with a drying gas temperature of 200 °C, gas flow of 8 l/min, nebulizer pressure of 35 psi, sheath gas temperature of 200 °C, sheath gas flow of 11 l/min, a fragmenter value of 175 V, skimmer voltage of 65 V and octapole 1 frequency of 750 Vpp. Data was analyzed and visualized using MassHunter Qualitative Analysis version 10.0 from Agilent.

Purification by preparative HPLC to isolate 6-O-α-D-glucopyranosyl-δ-CD and ε-CD and to obtain δ-CD of extra high purity was carried out with a Buchi C-850 FlashPrep Purification system equipped with an XBridge BEH Amide OBD Prep column (5 µm, 19 × 150 mm) from Waters. A Heraeus Biofuge Pico centrifuge equipped with a Sorvall Heraeus #3328 rotor was used for the preparation of samples for HPLC analysis.

An Eppendorf Centrifuge 5810R was used during the synthesis of δ-CD. UV-Vis absorption spectra were measured with a PerkinElmer Lambda 25 Spectrometer using a 3.5 ml Hellma Quartz cuvette with a 10 mm path length at room temperature.

S3 Synthesis of templates

S3.1 Synthesis of $Na_2B_{12}Cl_{12}$

The synthesis was performed according to a published procedure,[5] with one main modification: During the oxidation of $Na_2B_{12}H_{12}$ to $Na_2B_{12}Cl_{12}$, chlorine gas was supplied using an *in situ* chlorine generator instead of a chlorine gas cylinder.

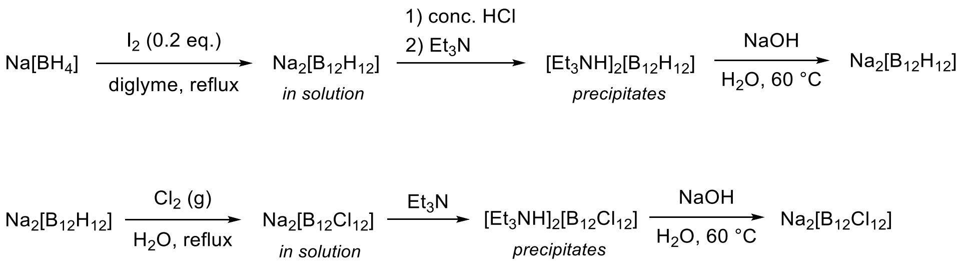

$[NHEt_3][B_{12}H_{12}]$. A suspension of $NaBH_4$ (47.0 g, 1.24 mol) in diglyme (200 ml) was heated to 100 °C under a nitrogen atmosphere. To this, a solution of iodine (103 g, 0.41 mol) in diglyme (175 ml) was then added dropwise. The dropwise addition was carried out over 24 hours. During the addition, the amount of insoluble $NaBH_4$ gradually decreased while the color of the reaction mixture became yellow. The reaction mixture was then stirred at 100 °C overnight, after which it was refluxed for another 24 hours. At this point, the reaction mixture had become a colorless suspension with white precipitate. The solvent was removed by vacuum distillation and the resulting white solid was dissolved in water (300 ml). Concentrated hydrochloric acid (145 ml) was added slowly, leading to the evolution of hydrogen gas. The solution was placed in a fridge at 5 °C overnight to precipitate boric acid as a white solid, which was removed by filtration. The filtrate was then treated with triethylamine (200 ml), leading to the precipitation of a white solid, which was collected by vacuum filtration, re-suspended in water (125 ml) and stirred for two hours to dissolve any remaining boric acid. The resulting suspension was then filtered by vacuum filtration and dried *in vacuo* to yield $[NHEt_3][B_{12}H_{12}]$ as a white solid (11.5 g, 33 mmol, 41%, based on iodine). NOTE: 5 equivalents of iodine are used to produce one $[B_{12}H_{12}]^{2-}$.

$Na_2[B_{12}H_{12}]$. $[NHEt_3][B_{12}H_{12}]$ (11.5 g, 33.8 mmol) was added to a solution of NaOH (2.78 g, 69 mmol) in water (120 ml) in a polypropylene beaker. The suspension formed was heated on a water bath (60 °C) until a clear solution was formed. The solvents were then removed *in vacuo* to yield $Na_2[B_{12}H_{12}]$ as a white solid (6.2 g, 33 mmol, quantitative).

$[Et_3NH]_2[B_{12}Cl_{12}]$. $Na_2[B_{12}H_{12}]$ (3.0 g, 16 mmol) was dissolved in water (30 ml) in a three-necked round-bottom flask fitted with a reflux condenser. This flask was then connected to a 'chlorine generator' flask and a 'chlorine trap' system. The original paper[6] contains important safety guidelines as well as practical considerations on how to set up the apparatus for this synthesis. The chlorine trap contained an aqueous solution of NaOH (1 M). The chlorine generator consisted of a three-necked round-bottom flask containing solid $Ca(ClO)_2$ (100 g, 0.70 mol) equipped with an addition funnel with concentrated hydrochloric acid (100 ml). Chlorine was then generated and bubbled through the solution of $Na_2[B_{12}H_{12}]$ at room temperature for 5 hours by the slow, dropwise addition of hydrochloric acid to the $Ca(ClO)_2$ solids (2 × 100 ml hydrochloric acid used). The reaction was then refluxed overnight (without chlorine generation). Another chlorine generator (same quantities of $Ca(ClO)_2$ and hydrochloric acid) was then set up, and chlorine was bubbled through the solution again for 6 hours at room temperature. The reaction was then refluxed overnight (no chlorine generation). The process of chlorine generation and bubbling through the reaction followed by reflux overnight was repeated once more. Completion of chlorination was checked by ^{11}B NMR spectroscopy over the course of the

reaction. After complete reaction, the resulting acidic, clear solution was treated with Et_3N (100 ml) leading to the immediate precipitation of a white solid. The solid was then isolated by vacuum filtration, washed repeatedly with water, and dried *in vacuo* to yield $[Et_3NH]_2[B_{12}Cl_{12}]$ as a white solid (7.33 g, 9.65 mmol, 60%).

$Na_2[B_{12}Cl_{12}]$. $[Et_3NH]_2[B_{12}Cl_{12}]$ (7.2 g, 9.5 mmol) was added to a solution of NaOH (0.80 g, 19.9 mmol) in water (80 ml) in a polypropylene beaker. The suspension formed was heated on a water bath (60 °C) until a clear solution was formed. The solvents were then removed *in vacuo* to yield $Na_2[B_{12}Cl_{12}]$ as a white solid (5.7 g, 9.5 mmol, quantitative).

S3.2 Synthesis of $Na_2B_{12}Br_{12}$

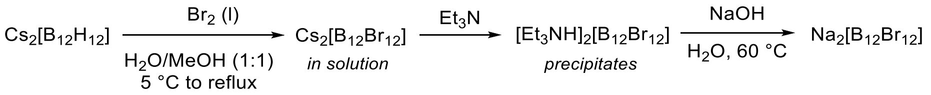

The synthesis was performed according to a published procedure.[7]

$[Et_3NH]_2[B_{12}Br_{12}]$. $Cs_2[B_{12}H_{12}]$ (400 mg, 0.98 mmol) was dissolved in water/methanol (1:1, 15 ml) in a three-necked round-bottom flask fitted with an addition funnel and placed in an ice bath at 5 °C. Bromine (2.0 ml, 39 mmol) was added dropwise under vigorously stirring for 3 hours. The ice bath was removed followed by another dropwise addition of bromine (4.0 ml, 79 mmol) under vigorously stirring for 5 hours. The reaction was then refluxed overnight, followed by removal of the solvents under vacuum. The white solid residue was redissolved in HCl (1 M, 20 ml) and treated with treated with Et_3N (5 ml) leading to the immediate precipitation of a white solid. The solid was then isolated by vacuum filtration, washed repeatedly with water, and dried *in vacuo* to yield $[Et_3NH]_2[B_{12}Br_{12}]$ as a white solid (1.21 g, 0.94 mmol, 95%).

$Na_2[B_{12}Br_{12}]$. $[Et_3NH]_2[B_{12}Br_{12}]$ (1.2 g, 0.93 mmol) was added to a solution of NaOH (78 mg, 1.95 mmol) in water (15 ml) in a polypropylene beaker. The suspension formed was heated on a water bath (60 °C) until a clear solution was formed. The solvents were then removed *in vacuo* to yield $Na_2[B_{12}Br_{12}]$ as a white solid (1.0 g, 0.93 mmol, quantitative).

S3.3 Synthesis of $Na_2B_{12}I_{12}$

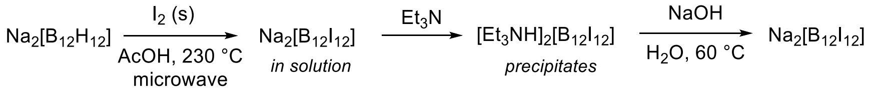

The synthesis was performed according to a published procedure.[7]

$[Et_3NH]_2[B_{12}I_{12}]$. In a 20 mL microwave vial $Na_2[B_{12}H_{12}]$ (200 mg, 1.06 mmol) and iodine (8.65 g, 34.1 mmol) were suspended in glacial acetic acid (10 ml) and heated at 230 °C for 2.5 h in a microwave reactor. The reaction mixture was then quenched with Na_2SO_3 (2.0 g) dissolved in water (50 ml) making a clear yellow solution containing a black precipitate, which was removed by filtration. The filtrate was treated with Et_3N (3 ml) leading to the immediate precipitation of a white solid. The solid was then isolated by vacuum filtration, washed repeatedly with water, and dried *in vacuo* to yield $[Et_3NH]_2[B_{12}I_{12}]$ as a white solid (1.84 g, 0.99 mmol, 93%).

$Na_2[B_{12}I_{12}]$. $[Et_3NH]_2[B_{12}I_{12}]$ (1.5 g, 0.81 mmol) was added to a solution of NaOH (68 mg, 1.7 mmol) in water (10 ml) in a polypropylene beaker. The suspension formed was heated on a water bath (60 °C) until a clear solution was formed. The solvents were then removed *in vacuo* to yield $Na_2[B_{12}I_{12}]$ as a yellow solid (1.4 g, 0.81 mmol, quantitative).

S4 Large scale synthesis of δ-CD

S4.1 General method for the synthesis of δ-CD

α-CD (10.00 g, 10.03 mmol) and $Na_2B_{12}Cl_{12}$ (3.00 g, 4.99 mmol) were dissolved in water (900 ml) and the pH adjusted to 7.5 using hydrochloric acid (1 M). The mixture was transferred to a 1-liter volumetric flask. A solution of CGTase (25 mL, commercial stock solution) was added to start the reaction and water was added until the 1-liter mark. The reaction mixture was transferred to a 2-liter round-bottomed flask and placed in a water bath at 30 °C for 42 hours, after which the mixture was boiled for 15 minutes to stop the reaction. The reaction mixture was concentrated to 0.2 l *in vacuo*, then centrifuged (60 min, 10000 rpm, 12074 RCF) and the supernatant decanted, leaving behind precipitated enzyme. The supernatant was then centrifuged two more times (60 min, 10000 rpm, 12074 RCF) to remove brown impurities (presumably impurities present in the CGTase stock solution). δ-CD was then precipitated by the addition of acetone (1.0 l) to the supernatant (0.2 l), and isolated by filtration. The solids (white, sometimes with an off-white/brownish tint) were then collected and dissolved in water (100 ml) and precipitated with acetone (0.5 l), followed by filtration to isolate the solids. This process was repeated 3 times, after which solids were lyophilized to yield the product δ-CD as a white solid (42 %). The spectroscopic data was in accordance with previously published results.[1] δ-CD was characterized analyzed using ^{1}H and ^{11}B NMR spectroscopy, UV-Vis spectroscopy and HPLC with ELS detection. A purity of 96% was determined by 1H NMR spectroscopy. See section S5 for details.

S4.2 Recovery of $Na_2B_{12}Cl_{12}$

The filtrate and washings from the isolation procedure of δ-CD (section S4.1) were combined and concentrated *in vacuo* to remove acetone and give a total volume of 0.2 l. Then HCl (37%, 20 ml) was added to reach pH 2. Triethylamine (15 ml) was then added in small portions while stirring leading to precipitation. The white precipitate that formed ($[Et_3NH]_2[B_{12}Cl_{12}]$) was collected by filtration and washed several times with cold water, then dried *in vacuo*. $[Et_3NH]_2[B_{12}Cl_{12}]$ could then be converted to $Na_2[B_{12}Cl_{12}]$ following the procedure described in section S3.1. (90% recovery, high purity confirmed by ^{1}H and ^{11}B NMR (Figure S6, S7). Note that the boron cluster was stored as $[Et_3NH]_2[B_{12}Cl_{12}]$ and converted to $Na_2[B_{12}Cl_{12}]$ as needed for δ-CD synthesis, as the sodium salt proved to be highly hygroscopic.

S4.3 Methods for further purification of δ-CD

δ-CD obtained using the general procedure (section S4.1) contains small amounts of unidentified UV-active impurities (originating from the enzyme stock solution and most likely protein and peptides) and small amounts of oligosaccharides: **G4–G7** and α-, β-, and γ-CD (see section S5). Higher purities of δ-CD can be achieved as described in the following section.

S4.4 Spin filtration

The UV-active impurities can be avoided in the final product by substituting the second and third centrifugation steps in the general procedure with a spin-filtration step. The supernatant from after first centrifugation step (that removed precipitated enzyme) was added to spin filter tubes with a 30 kDa threshold (10 ml per tube) and centrifuged (10 min, 10000 rpm, 12074 RCF) to remove dissolved enzyme and protein residue. δ-CD was then precipitated from the filtrate of the spin-filtration by the addition of acetone (0.5 l), followed by filtration to isolate the solids. Further precipitations were then carried out as described in the general procedure (section S4.1).

S4.5 Preparative HPLC

The UV-active impurities as well as small amounts of **G4–G7** and α-, β-, and γ-CD present in the δ-CD obtained from the general procedure (section **S4.1**) can be removed using preparative HPLC. δ-CD (41 mg, dissolved in 0.5 ml water) was injected on a HILIC column (XBridge BEH Amide OBD Prep column from Waters, 130 Å, 5 µm, 19 × 150 mm) using a Buchi C-850 FlashPrep Purification system equipped with an ELS detector. Gradient elution was performed: 7 ml/min flow rate, 25% water in acetonitrile to 43% water in acetonitrile over 50 minutes. δ-CD eluted from 40 to 43 minutes. All fractions containing δ-CD were combined, concentrated, and lyophilized. 39 mg (95%) of purified δ-CD were recovered.

^{1}H NMR (400 MHz, D_2O) *δ* 5.27 (d, *J* = 3.9 Hz, 9H, **H1**), 3.97 (t, *J* = 9.6 Hz, 9H, **H3**), 3.94 – 3.81 (m, 27H, **H5**, **H6**), 3.65 (t, *J* = 9.6 Hz, 9H, **H4**), 3.62 (dd, *J* = 9.6, 3.9 Hz, **H2**). **^{13}C NMR** (101 MHz, D_2O, referenced to residual solvent in DMSO-*d6* locktube) δ 98.9 (**C1**), 77.0 (**C4**), 71.9 (**C3**), 71.2 (**C2**), 70.3 (**C5**), 59.3 (**C6**). **HRMS** (ESI) (*m/z*): $[M+H]^+$ calcd. for $C_{54}H_{91}O_{45}$ 1459.4827; found 1459.4818.

S5 Characterization of batches of δ-CD

The following section contains the characterization of δ-CD obtained by the general method (Section 4.1) shows evidence of the purity of the $Na_2B_{12}Cl_{12}$ recovered after the reaction (Section 4.2) and documents how the different methods of further purification (Section 4.3) are successful in further removing linear and cyclic side products, $Na_2B_{12}Cl_{12}$ and peptide and protein residue from the commercial CGTase stock solution.

S5.1 NMR spectra

δ-CD: Synthesized via the general method (i.e. 3 rounds of precipitation, no chromatography, obtained in 42% yield and 96% purity). Note: there are very small amounts of other CDs, linear glucans, and protein and peptide residue (1.2–2.2 ppm). After 3 rounds of precipitation there is residual $B_{12}Cl_{12}^{2-}$ present, but after 5 rounds of precipitation this is removed.

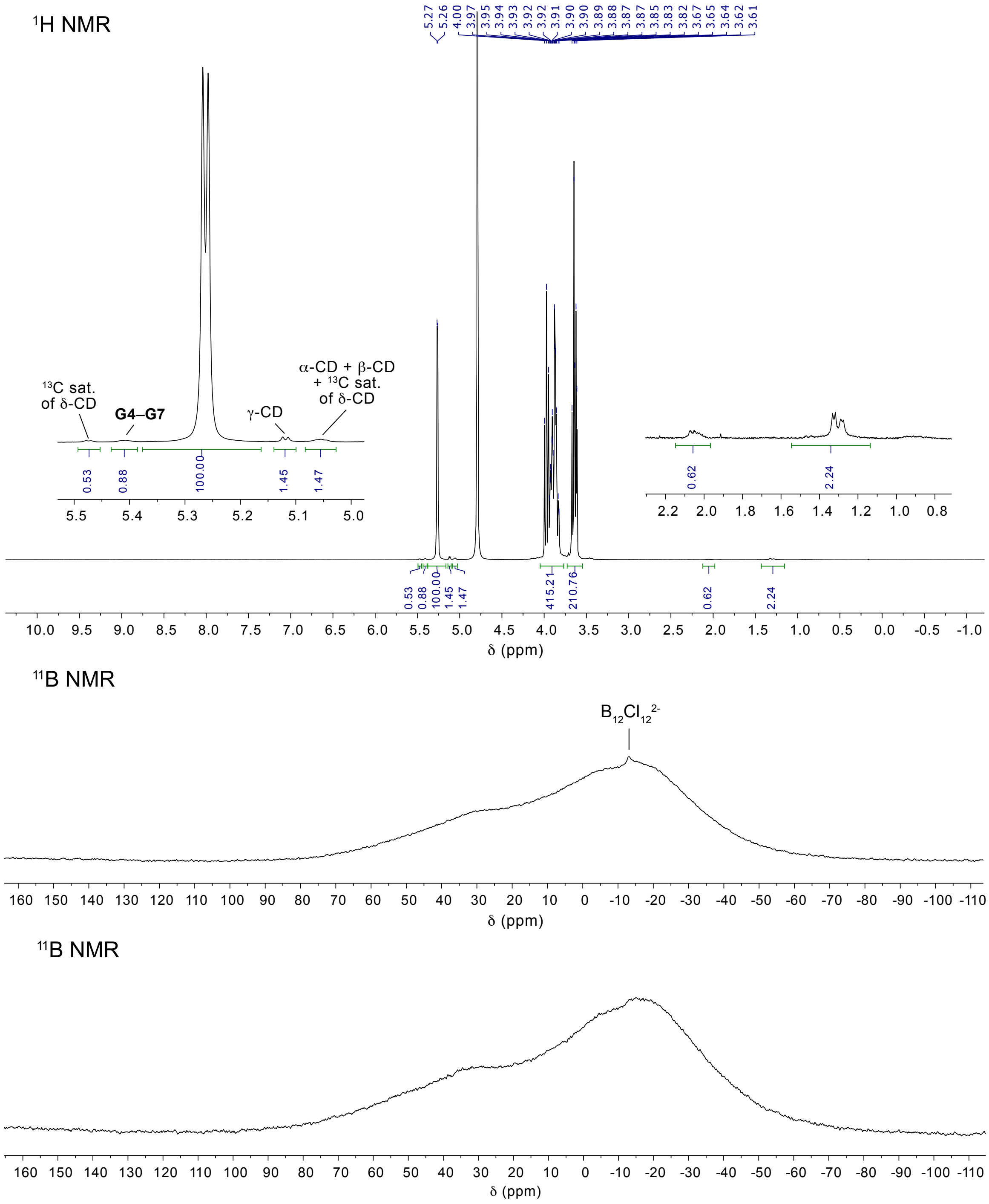

Figure S1. NMR spectra (^{1}H: 400 MHz, ^{11}B: 128 MHz) of δ-CD (8 mg/ml in D_2O in a standard borosilicate glass NMR tube) obtained using the general procedure described in **S4.1** and after a total of 3 precipitations (*top* and *middle*) or 5 precipitations (*bottom*). Identified impurities noted on the figure. ^{13}C sat.: ^{13}C satellite. **G4–G7**: Linear α-1,4-glucans with 4–7 glucose units.

δ-CD: Synthesized via the general method, but with an additional spin filtration step to removed dissolved protein material as described in Section 4.3. Note that the signals at 1.2–2.2 are much reduced.

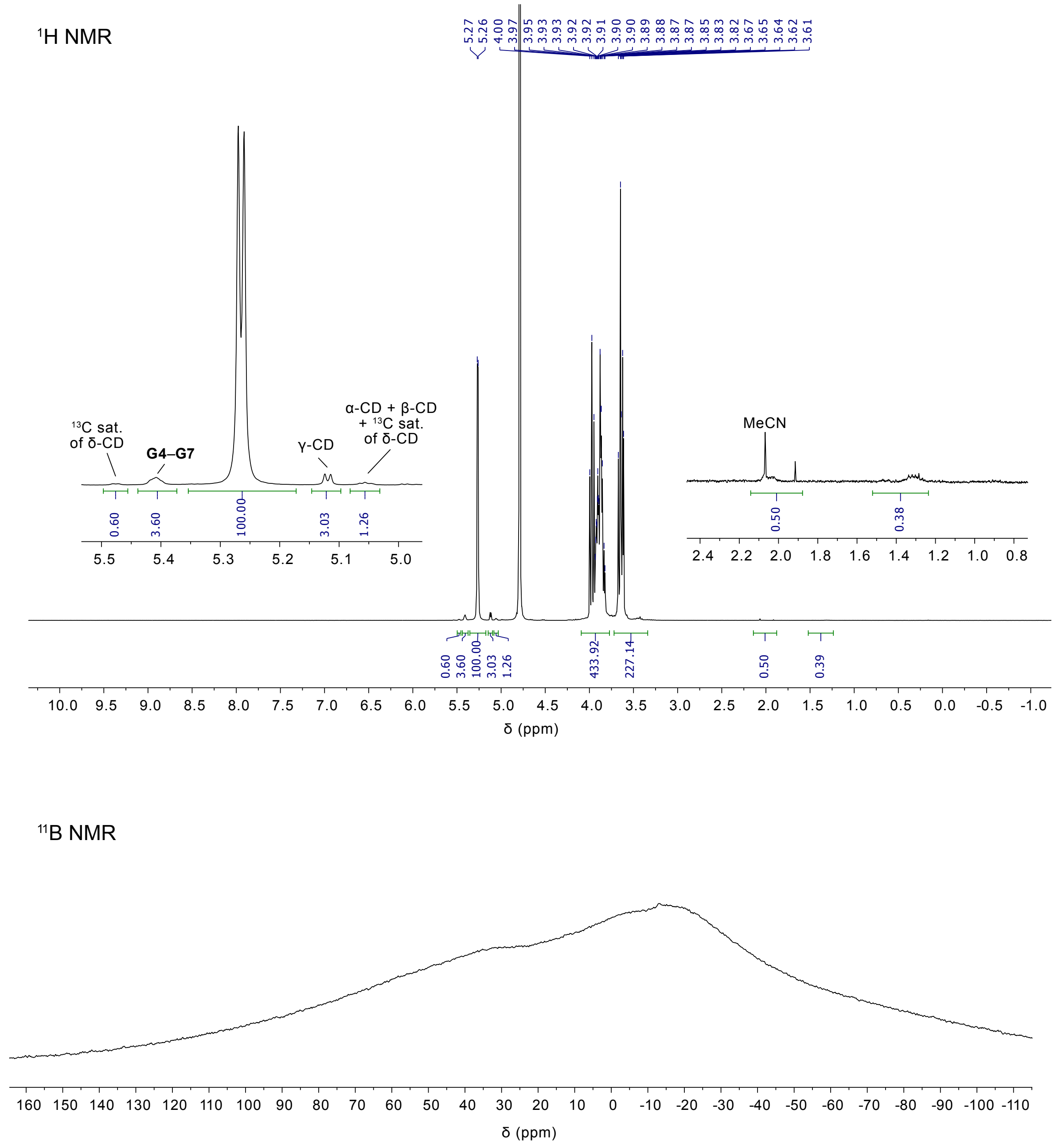

Figure S2. NMR spectra (^{1}H: 400 MHz, ^{11}B: 128 MHz) of a batch of δ-CD (8 mg/ml in D_2O in a standard borosilicate glass NMR tube) obtained using the general procedure described in section S4.1 and the modification described in section S4.4 (spin filtration of reaction mixture) followed by 5 precipitations. Identified impurities denoted on figure. ^{13}C sat.: ^{13}C satellite. **G4–G7**: Linear α-1,4-glucans with 4–7 glucose units.

δ-CD: Synthesized via the general method and then further purified by HPLC. Note that all impurities have been removed, including other glucans, residual boron cluster and protein/peptide residues.

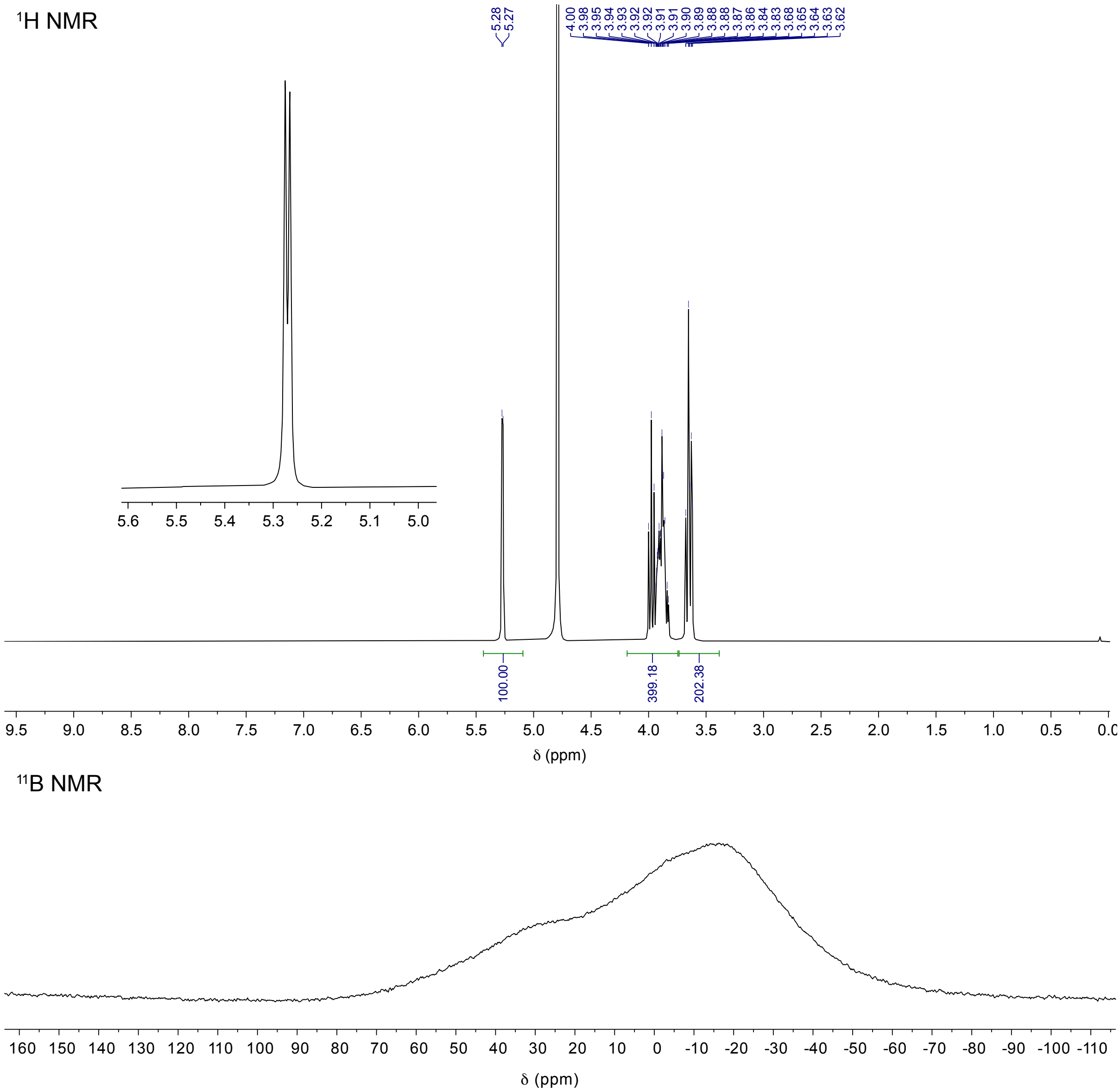

Figure S3. NMR spectra (^{1}H: 400 MHz, ^{11}B: 128 MHz) of a batch of δ-CD (6 mg/ml in D_2O in a standard borosilicate glass NMR tube) obtained using the general procedure described in section S4.1 with 5 precipitations followed by isolation using preparative HPLC as described in section S4.5. Identified impurities denoted on figure.

S5.2 HPLC-ELSD chromatograms

Below is shown HPLC-ELSD chromatograms for δ-CD after purification by 1, 3, or 5 rounds of precipitation (A–C), with spin filtration prior to precipitation (D) and by HPLC (E). Note that this analysis is insufficient to check the purity of the synthesized δ-CD as small amounts of other CDs and linear glucans were detectable in samples B–D by ^{1}H NMR spectroscopy but are below the limit of detection in this analysis.

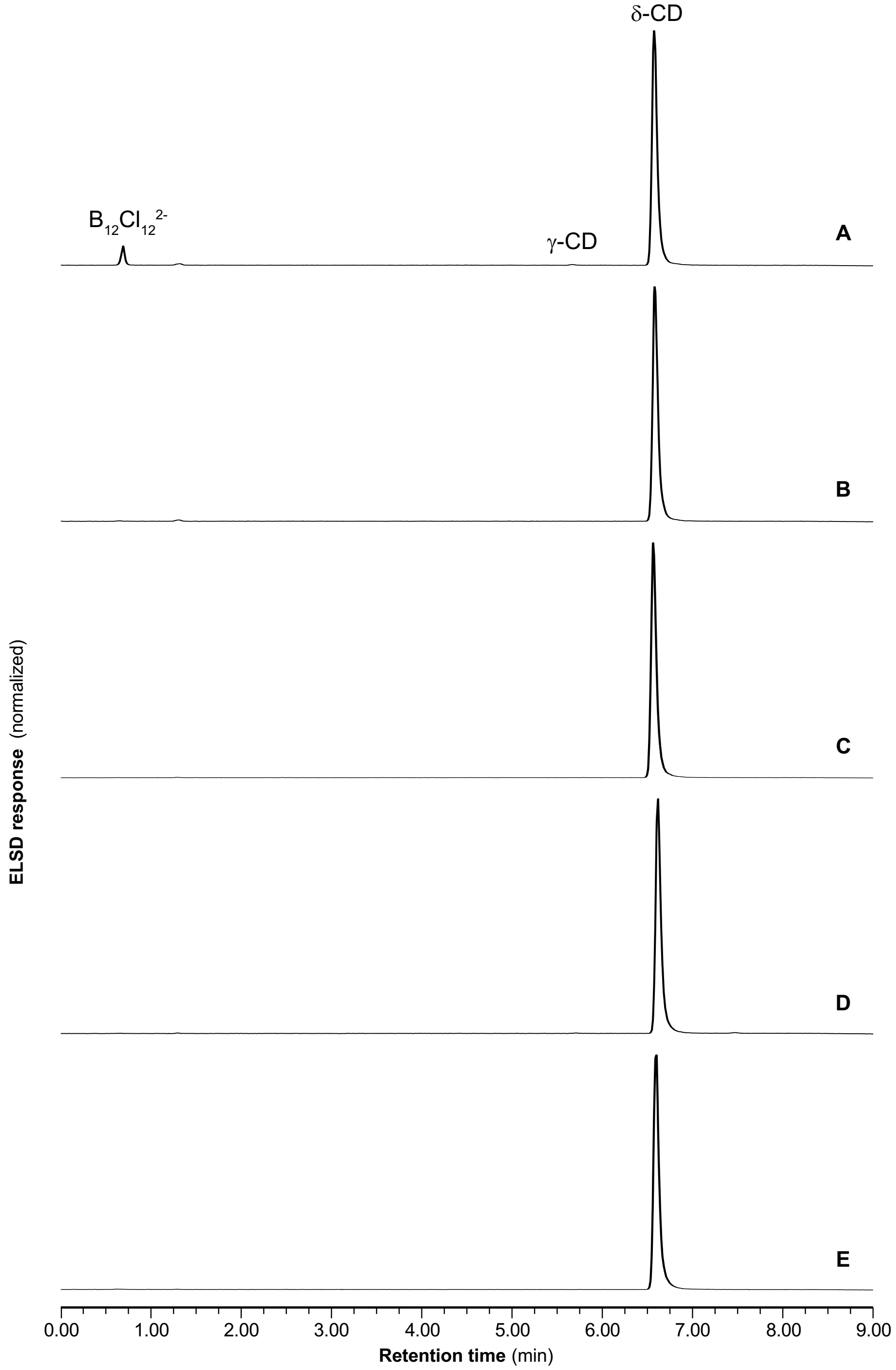

Figure S4. HPLC-ELSD chromatograms of various batches of δ-CD. (A), (B), (C): Obtained using the general procedure described in section S4.1 and after a total of 1, 3 and 5 precipitations, respectively. (D): Obtained using the modification described in section S4.4 (spin filtration of reaction mixture) followed by 5 precipitations. (E): Obtained by isolating δ-CD from (C) using preparative HPLC as described in section S4.5.

S5.3 UV-Vis spectra

Optically-active impurities were detected in the 96% pure δ-CD produced according to the general method (Section S4.1) and purified only by precipitation. Spectroscopically pure δ-CD could be obtained either by including a spin filtration step in the precipitation procedure (Section S4.4) or by purification using HPLC (Section S4.5).

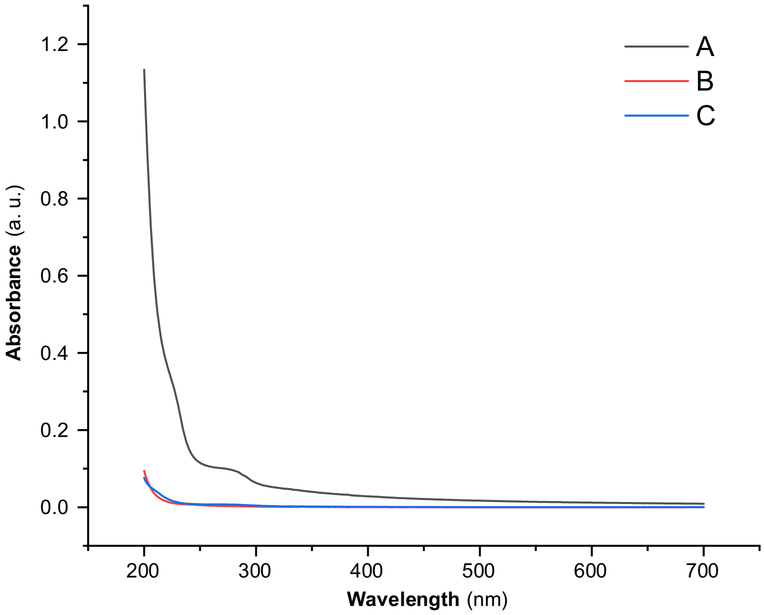

Figure S5. UV-vis absorption spectra of various batches of δ-CD. (A): Obtained using the general procedure described in section S4.1 and after a total of 5 precipitations. (B): Obtained using the modification described in section S4.4 (spin filtration of reaction mixture) followed by 5 precipitations. (C): Obtained by isolating δ-CD from (A) using preparative HPLC as described in section S4.5.

S5.4 Analysis of recovered $Na_2B_{12}Cl_{12}$ after δ-CD synthesis

To confirm that the recovered boron cluster was successfully isolated after use as a template for the production of δ-CD, it was analyzed using ^{1}H and ^{11}B NMR spectroscopy.

^{1}H NMR spectra of $Na_2B_{12}Cl_{12}$ before and after δ-CD synthesis

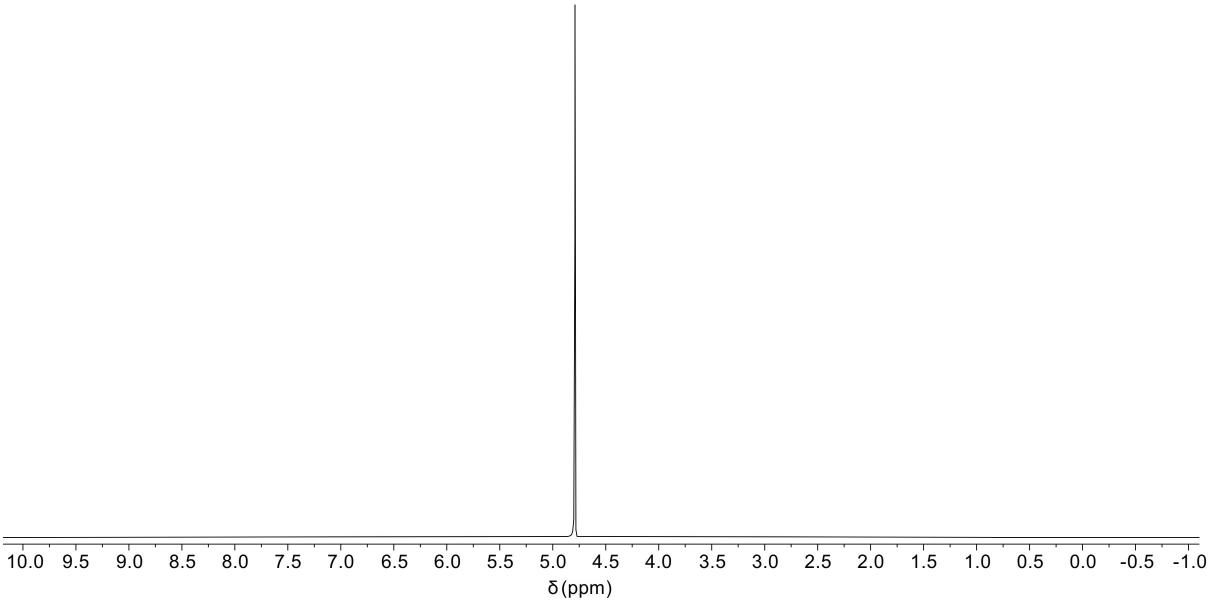

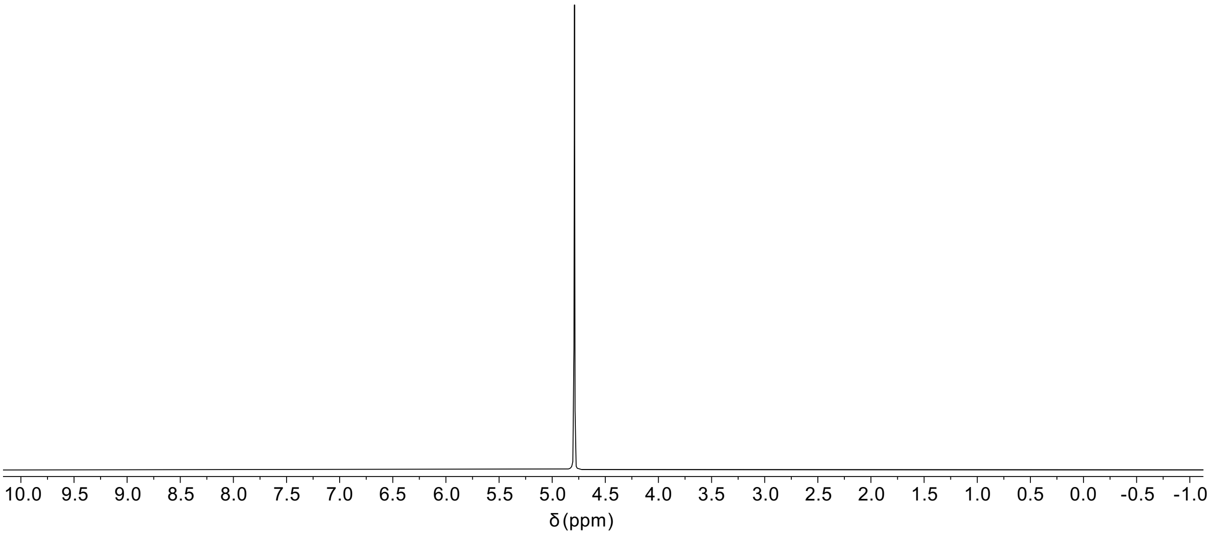

Figure S6. ^{1}H NMR (400 MHz) spectra of $Na_2B_{12}Cl_{12}$ in D_2O before (top) and after (bottom) recovery from an enzymatic δ-CD production (as described in sections S3.1 and S4.2. respectively). Note that no peaks are expected, and indeed only the solvent peak is observed in both cases.

^{11}B NMR spectra of $Na_2B_{12}Cl_{12}$ before and after δ-CD synthesis

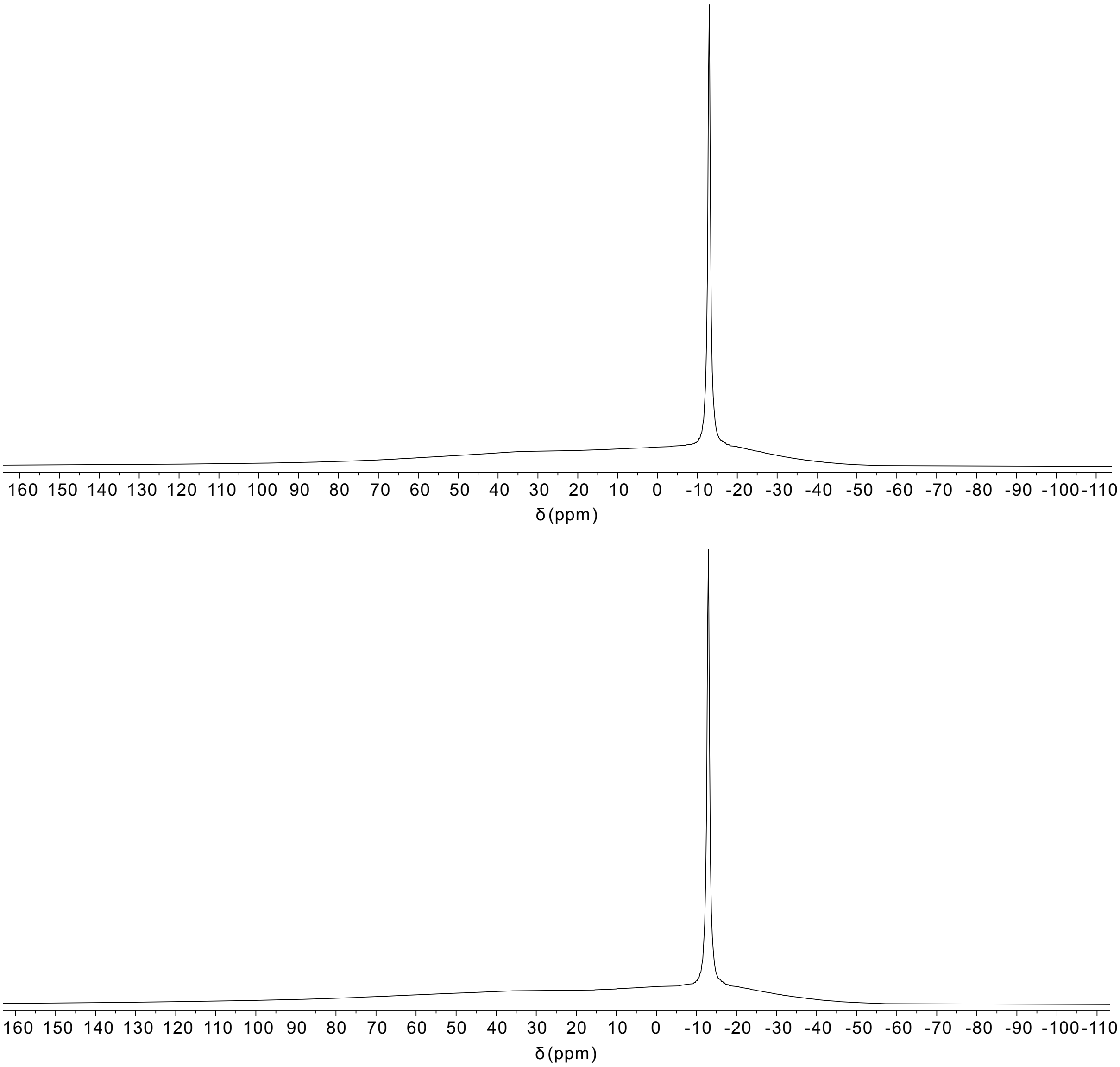

Figure S7. ^{11}B NMR (128 MHz) spectra of $Na_2B_{12}Cl_{12}$ in D_2O before (top) and after (bottom) recovery from an enzymatic δ-CD production (as described in sections S3.1 and S4.2, respectively).

S6 Synthesis and isolation of ε-CD (CD10)

α-CD (0.20 g) and $Na_2B_{12}I_{12}$ (0.17 g, 0.10 mmol) were dissolved in water (15 ml). Sodium phosphate buffer (50 mM, pH 7.5, 4.0 ml) and commercial CGTase stock solution (1.0 ml) were then added to give a total reaction volume of 20 ml and final concentrations of α-CD (10 mg/ml), $Na_2B_{12}I_{12}$ (5 mM), sodium phosphate buffer (10 mM) and CGTase (50 µl stock per ml reaction mixture). The reaction was kept at room temperature for 3 hours and then heated to 100 ºC for 15 min to stop the reaction. The reaction mixture was then centrifuged (20 min, 2200 rpm, 785 RCF) and the supernatant collected and concentrated *in vacuo* until 10 ml remained. Acetone (35 ml) was then added, and the resulting precipitate was isolated by centrifugation (10 min, 2200 rpm, 785 RCF). The solids were then dissolved in water (1.3 ml) and filtered through a syringe filter. The solution was then split into two portions and each portion injected on a HILIC type column (XBridge BEH Amide OBD Prep column from Waters, 130 Å, 5 µm, 19 × 150 mm) using a Buchi C-850 FlashPrep Purification system equipped with an ELS detector. Gradient elution was performed: 7 ml/min flow rate, 25% water in acetonitrile to 45% water in acetonitrile over 55 min. ε-CD eluted from 53 to 55 min. All fractions containing ε-CD were combined, concentrated, and lyophilized. Yield: 2.7 mg, 1.4%.

^{1}H NMR (400 MHz, D_2O) δ 5.36 (d, *J* = 3.9 Hz, 10H, **H1**), 4.01 (t, *J* = 9.5 Hz, 10H, **H3**), 3.94 (m, 10H, **H5**), 3.89 (s, 10H, **H6**), 3.83 (dd, *J* = 12.3, 4.8 Hz, 10H, **H6'**), 3.67 (t, *J* = 9.5 Hz, 10H, **H4**), 3.62 (dd, *J* = 9.5, 3.9 Hz, 10H, **H2**). **HRMS** (ESI) *m/z*: $[M+H]^+$ calcd. for $C_{60}H_{101}O_{50}^+$: 1621.5355; found: 1621.5355.

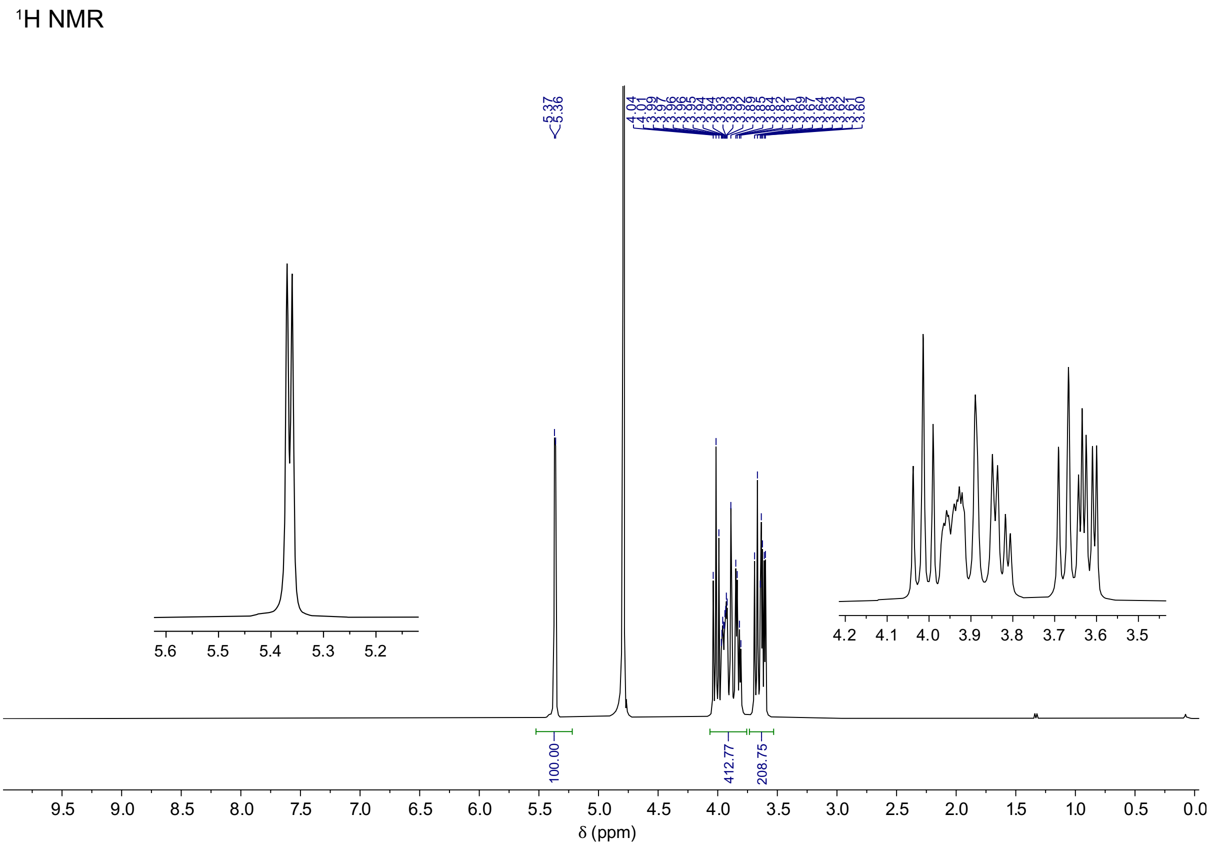

Figure S8. ^{1}H NMR spectrum (400 MHz) of ε-CD in D_2O.

S7 Synthesis of 6-O-α-D-glucopyranosyl-δ-cyclodextrin (G1-δ-CD)

A stock solution of soluble starch (15 mg/ml) in sodium phosphate buffer (50 mM, pH 6.0) was prepared by heating starch and buffer in the microwave (8 heating cycles with vigorous mixing between the cycles) until dissolution. Starch stock solution (5 ml), a stock solution of $Na_2B_{12}Cl_{12}$ (50 mM, 0.75 ml) in sodium phosphate buffer (50 mM, pH 6.0) and sodium phosphate buffer (50 mM, pH 6.0, 1.75 ml) were mixed with commercial CGTase stock solution (0.375 ml) to give a total reaction volume of 7.5 ml and final concentrations of starch (10 mg/ml), $Na_2B_{12}Cl_{12}$ (5 mM) and CGTase (50 µl stock per ml reaction mixture). The reaction was kept at room temperature for eight days and then heated to 100 ºC for 15 min to stop the reaction. The reaction mixture was then centrifuged (5 min, 2200 rpm, 785 RCF) and the supernatant collected and concentrated *in vacuo*. The solids were then dissolved in water (0.6 ml) and the solution filtered through a syringe filter and injected on a HILIC type column (XBridge BEH Amide OBD Prep column from Waters, 130 Å, 5 µm, 19 × 150 mm) using a Buchi C-850 FlashPrep Purification system equipped with an ELS detector. Gradient elution was performed: 7 ml/min flow rate, 25% water in acetonitrile to 43% water in acetonitrile over 49.5 min, then from 43% to 45% acetonitrile in water over 8 min. G1-δ-CD eluted from 54 to 56 min. All fractions containing G1-δ-CD were combined, concentrated, and lyophilized. Yield: 1.8 mg, 2.4%.

S7.1 Characterization of G1-δ-CD

G1-δ-CD has the same molecular mass as ε-CD (CD10) but had a different retention time when analyzed using HPLC as described in section S2. G1-δ-CD isolated as described above contains impurities, presumably corresponding to branched non-cyclic maltodextrins with 7 and 8 glucose units containing both α-1,4-, and α-1,6-linkages, as suggested by the ^{1}H NMR spectrum (Figure S9) the ESI-MS spectrum (Figure S10). The ^{1}H NMR spectrum of G1-δ-CD is analogous to the ^{1}H NMR spectra of G1-α-CD and G1-β-CD reported in the literature.[8]

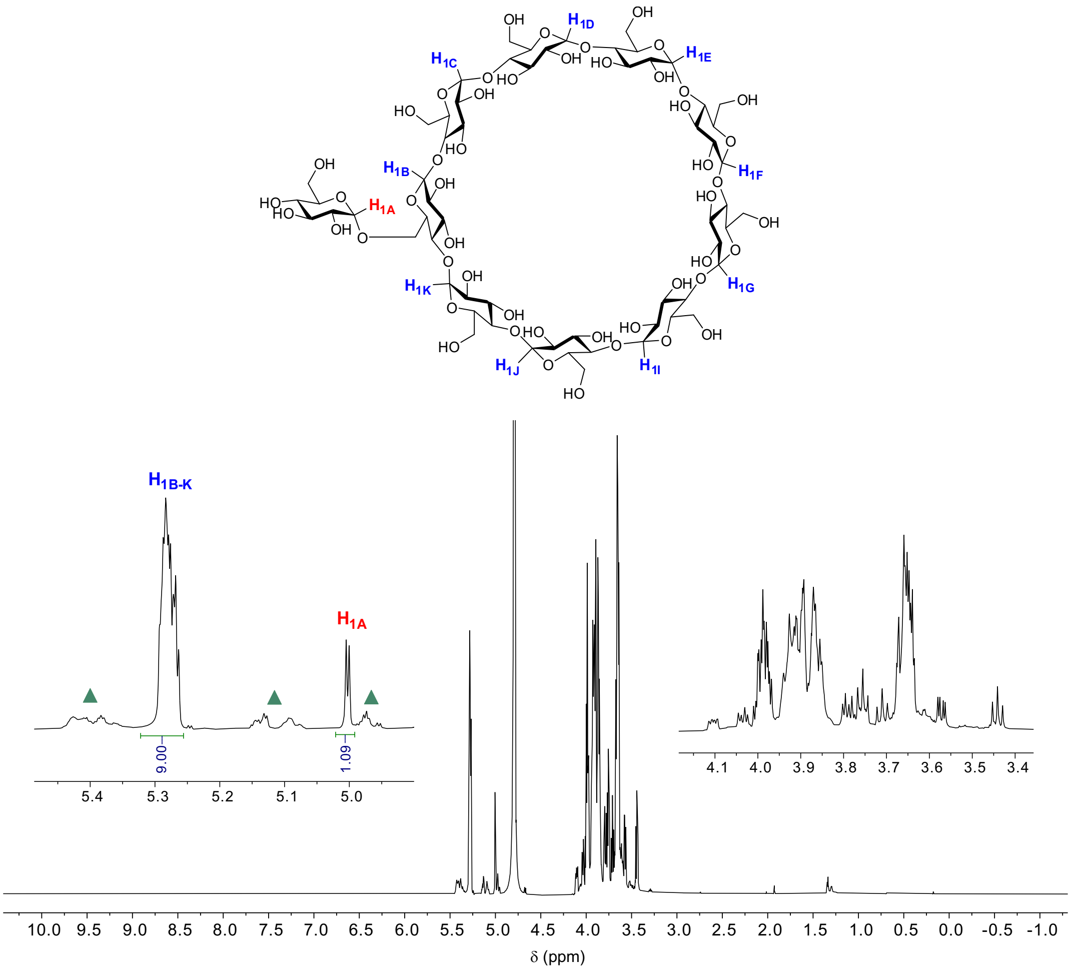

Figure S9. ^{1}H NMR spectrum (800 MHz) of G1-δ-CD in D_2O. The anomeric (H1) protons of G1-δ-CD follow the same pattern reported for G1-α-CD and G1-β-CD:[8] The H1 protons next to 1,4-linkages, **H1B-K**, give rise to overlapping signals (relative integral: ~9) with roughly the same chemical shift as the H1 protons of the unsubstituted CD, while the H1 proton of the 1,6-substiuted glucose unit (**H1A**) gives rise to a doublet at roughly 5 ppm (relative integral: ~1). Green triangles: Peaks presumably corresponding to branched non-cyclic maltodextrins containing both α-1,4-, and α-1,6-linkages.

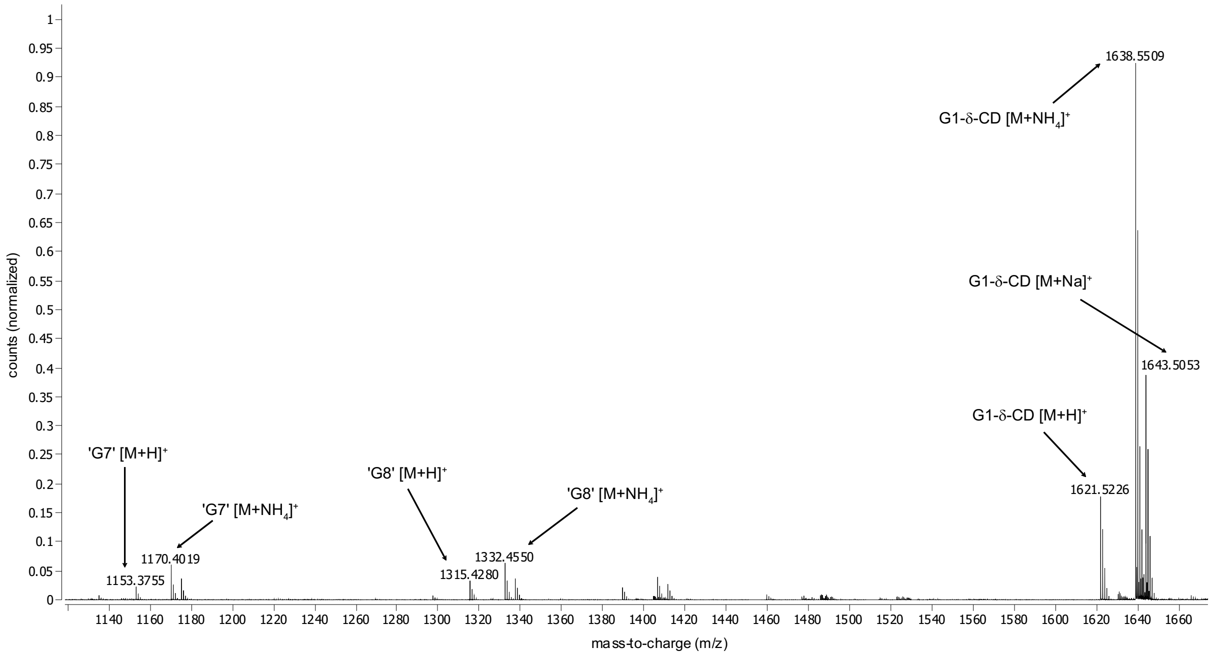

Figure S10. ESI-MS spectrum of G1-δ-CD. 'G7' and 'G8' correspond to non-cyclic maltodextrins (linear or branched) with a total of 7 or 8 glucose units, respectively.

S8 CGTase-mediated Dynamic Combinatorial Libraries (DCLs) at an analytical scale

S8.1 CGTase-mediated DCLs with α-CD as starting material and $Na_2B_{12}Cl_{12}$ as template

Stock solutions of α-CD (20 mg/mL) and $Na_2B_{12}Cl_{12}$ (50 mM) in sodium phosphate buffer (50 mM, pH 7.5) were mixed with sodium phosphate buffer (50 mM, pH 7.5) and spin-filtered CGTase stock solution to give a total reaction volume of 100 µl and final concentrations of α-CD (10 mg/ml), $Na_2B_{12}Cl_{12}$ (2–10 mM) and CGTase (65 µl stock solution per ml reaction mixture). The reactions were kept at room temperature and monitored at various time points. Aliquots for analysis (3 µl) were removed and the enzymatic reaction was stopped by immediate addition to a quenching mixture (90 µl) consisting of 1% trifluoroacetic acid in acetonitrile/water (3:1) with ammonium chloride (10 mM). The samples were centrifuged (4 min, 10000 rpm, 6708 RCF) and the top 80 µl fractions were transferred to HPLC vials for analysis.

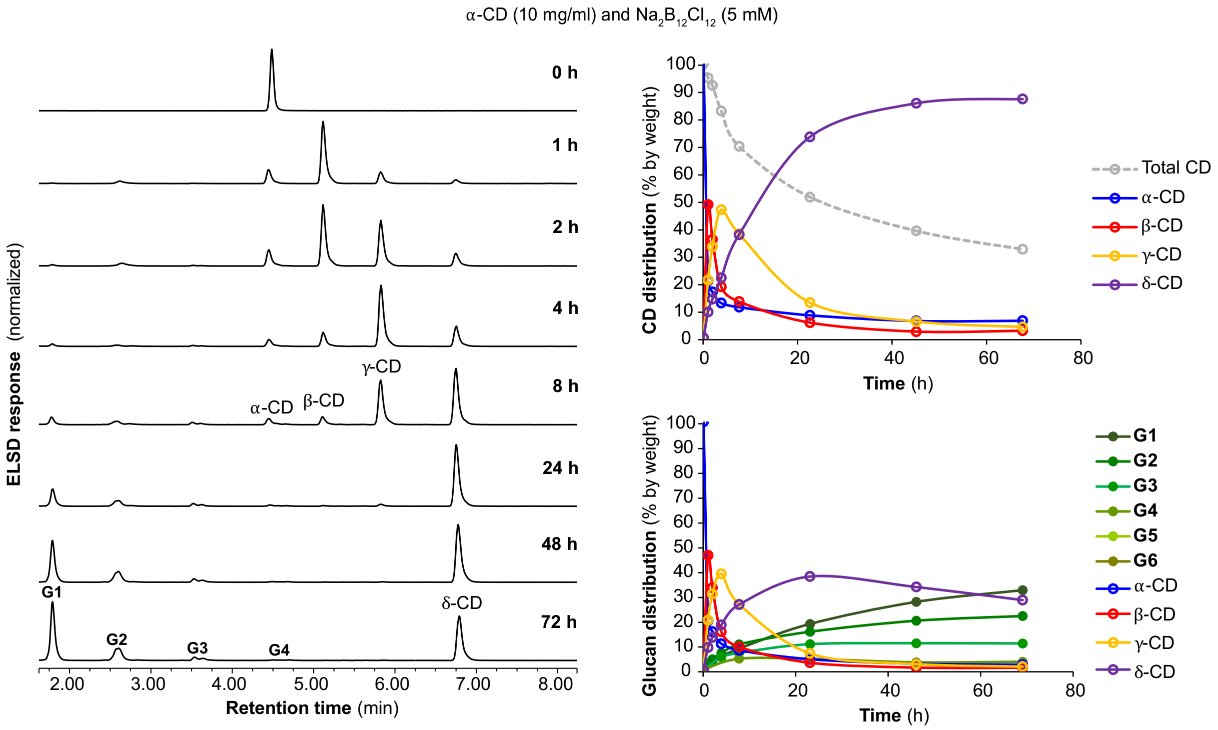

Figure S11. Reaction monitoring of the CGTase-mediated reaction with α-CD (10 mg/ml) in the presence of $Na_2B_{12}Cl_{12}$ (5 mM). *Left:* HPLC-ELSD chromatograms from the reaction. *Top right*: Distribution within the CD subsystem (α-, β-, γ-, δ-CD) and % of glucans that are CDs. *Bottom right*: Distribution of all glucans in the reaction mixture including, CDs (α-, β-, γ-, δ-CD) and linear α-1,4-glucans (**G1**–**G6**).

S8.2 CGTase-mediated DCLs with different concentrations of $Na_2B_{12}Cl_{12}$ template

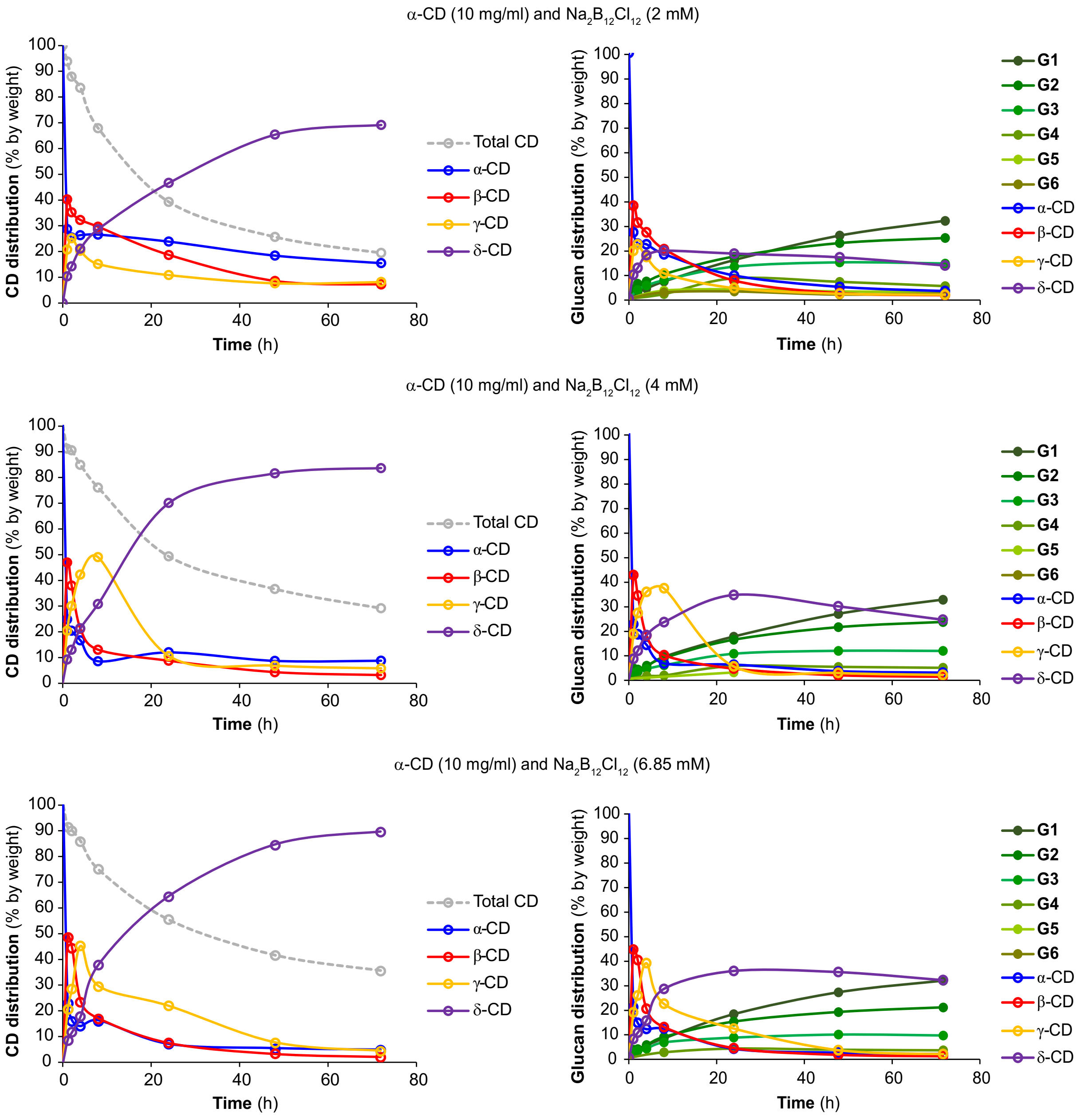

Figure S12. Reaction monitoring of the CGTase-mediated reaction of α-CD (10 mg/ml) in the presence of $Na_2B_{12}Cl_{12}$ (at concentrations as indicated on figure). *Left*: Distribution within the CD subsystem (α-, β-, γ-, δ-CD) and % of all glucans present that are CDs. *Right*: Distribution of all glucans in the reaction mixture including CDs (α-, β-, γ-, δ-CD) and linear α-1,4-glucans (**G1**–**G6**).

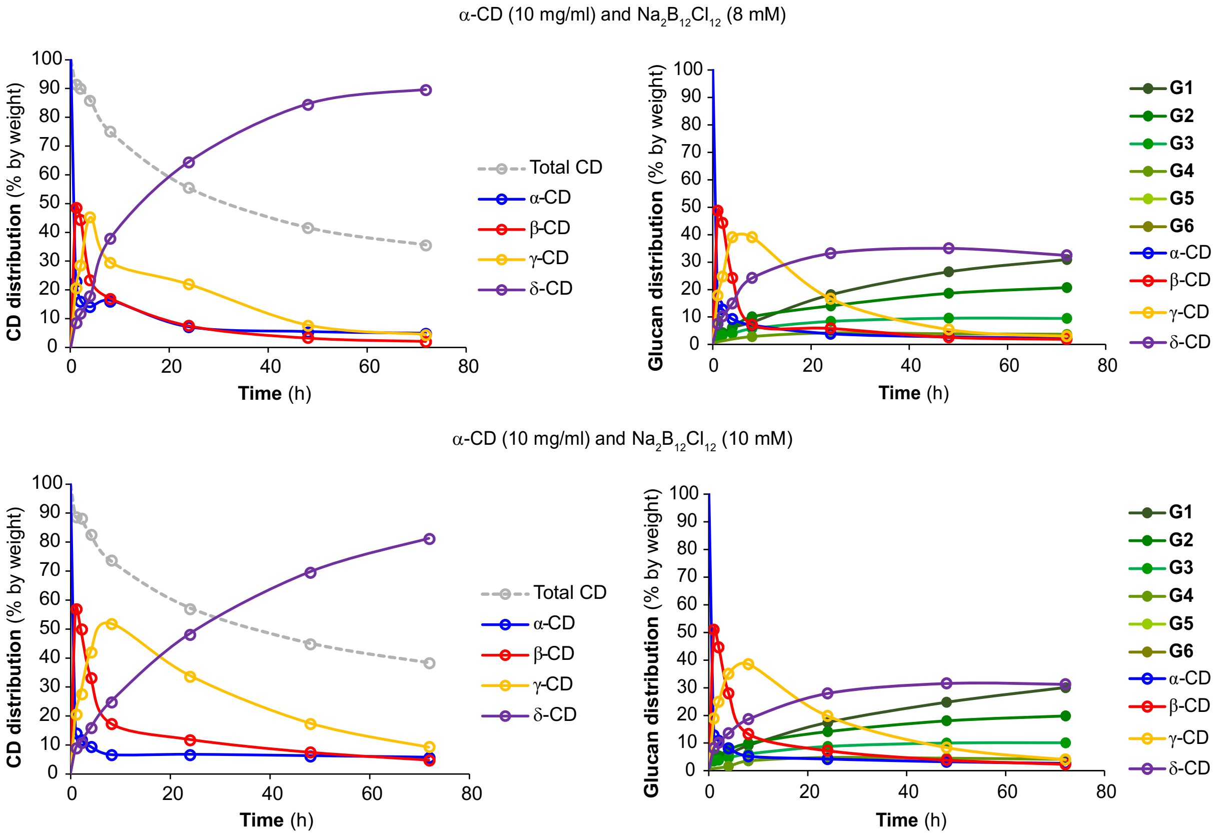

Figure S12 (continued). Reaction monitoring of the CGTase-mediated reaction of α-CD (10 mg/ml) in the presence of $Na_2B_{12}Cl_{12}$ (at concentrations as indicated on figure). *Left*: Distribution within the CD subsystem (α-, β-, γ-, δ-CD) and % of all glucans present that are CDs. *Right*: Distribution of all glucans in the reaction mixture including CDs (α-, β-, γ-, δ-CD) and linear α-1,4-glucans (**G1**–**G6**).

S8.3 CGTase-mediated DCLs with $Na_2B_{12}Br_{12}$ and $Na_2B_{12}I_{12}$ as templates

Stock solutions of α-CD (20 mg/mL) and $Na_2B_{12}Br_{12}$ or $Na_2B_{12}I_{12}$ (10 mM) in sodium phosphate buffer (50 mM, pH 7.5) were mixed with sodium phosphate buffer (50 mM, pH 7.5) and spin-filtered CGTase stock solution to give a total reaction volume of 100 µl and final concentrations of α-CD (10 mg/ml), $Na_2B_{12}Br_{12}$ or $Na_2B_{12}I_{12}$ (5 mM) and CGTase (65 µl stock solution per ml reaction mixture). The reactions were kept at room temperature and monitored at various time points. Aliquots for analysis (3 µl) were removed and the enzymatic reaction was stopped by immediate addition to a quenching mixture (90 µl) consisting of 1% trifluoroacetic acid in acetonitrile/water (3:1) with ammonium chloride (10 mM). The samples were centrifuged (4 min, 10000 rpm, 6708 RCF) and the top 80 µl fractions were transferred to HPLC vials for analysis.

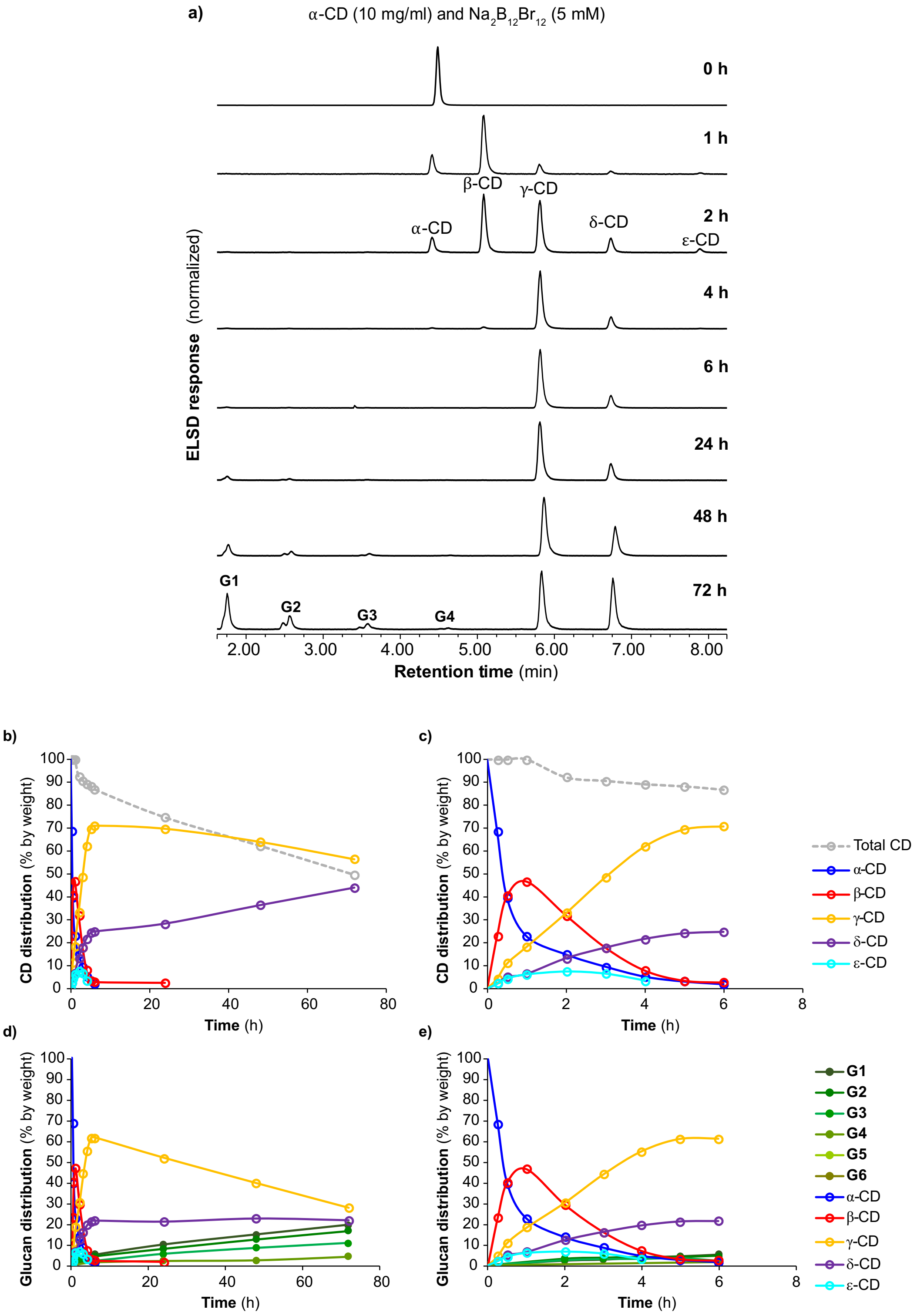

Figure S13. Reaction monitoring of the CGTase-mediated reaction with α-CD (10 mg/ml) in the presence of $Na_2B_{12}Br_{12}$ (5 mM). (a) HPLC-ELSD chromatograms from the reaction. (b, c) Distribution within the CD subsystem (α-, β-, γ-, δ-, ε-CD) and % of all glucans present that are CDs in the library (over 72 hours and during the first 6 hours). (d, e). Distribution of all glucans in the library, including CDs (α-, β-, γ-, δ-, ε-CD) and linear α-1,4-glucans (**G1**–**G6**) (over 72 hours and during the first 6 hours).

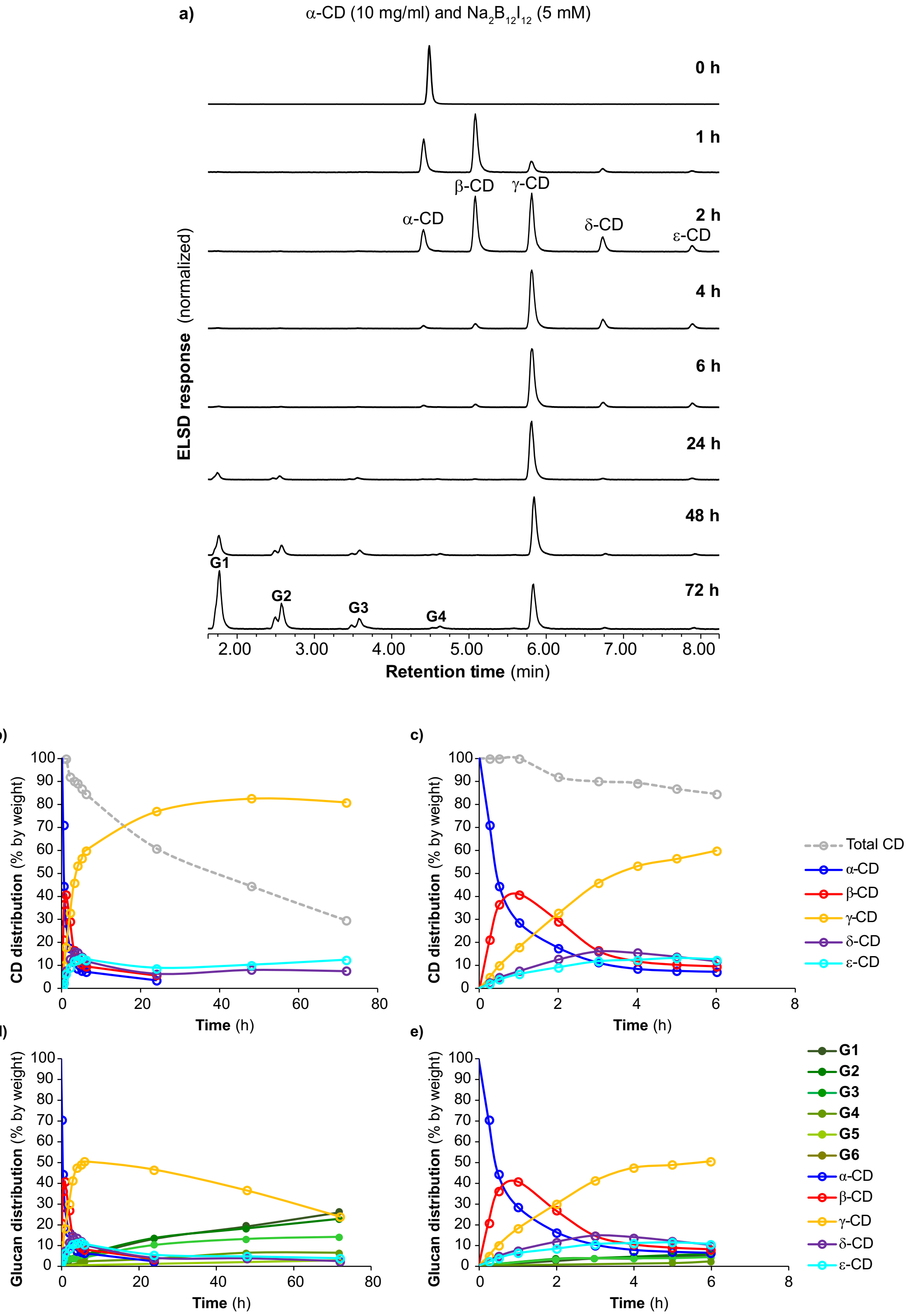

Figure S14. Reaction monitoring of the CGTase-mediated reaction with α-CD (10 mg/ml) in the presence of $Na_2B_{12}I_{12}$ (5 mM). (a) HPLC-ELSD chromatograms from the reaction. (b, c) Distribution within the CD subsystem (α-, β-, γ-, δ-, ε-CD) and % of all glucans present that are CDs in the library (over 72 hours and during the first 6 hours). (d, e) Distribution of all glucans in the library, including CDs (α-, β-, γ-, δ-, ε-CD) and linear α-1,4-glucans (**G1**–**G6**) (over 72 hours and during the first 6 hours).

S8.4 CGTase-mediated DCL with starch as starting material and $Na_2B_{12}Cl_{12}$ as template

A stock solution of soluble starch (24 mg/ml) in sodium phosphate buffer (50 mM, pH 7.5) was prepared by heating starch and buffer in the microwave (8 heating cycles with vigorous mixing between the cycles) until dissolution. Starch stock solution (83.3 µl), a stock solution of $Na_2B_{12}Cl_{12}$ (50 mM, 20 µl) in sodium phosphate buffer (50 mM, pH 7.5) and sodium phosphate buffer (50 mM, pH 7.5, 83.7 µl) were mixed with spin-filtered CGTase stock solution (13 µl) to give a total reaction volume of 200 µl and final concentrations of starch (10 mg/ml), $Na_2B_{12}Cl_{12}$ (5 mM) and CGTase (65 µl stock solution per ml reaction mixture). The reaction was kept at room temperature and monitored at various time points. Aliquots for analysis (3 µl) were removed and the enzymatic reaction was stopped by immediate addition to a quenching mixture (90 µl) consisting of 1% trifluoroacetic acid in acetonitrile/water (3:1) with ammonium chloride (10 mM). The samples were centrifuged (8 min, 10000 rpm, 6708 RCF) and the top 83 µl fractions were transferred to HPLC vials for analysis.

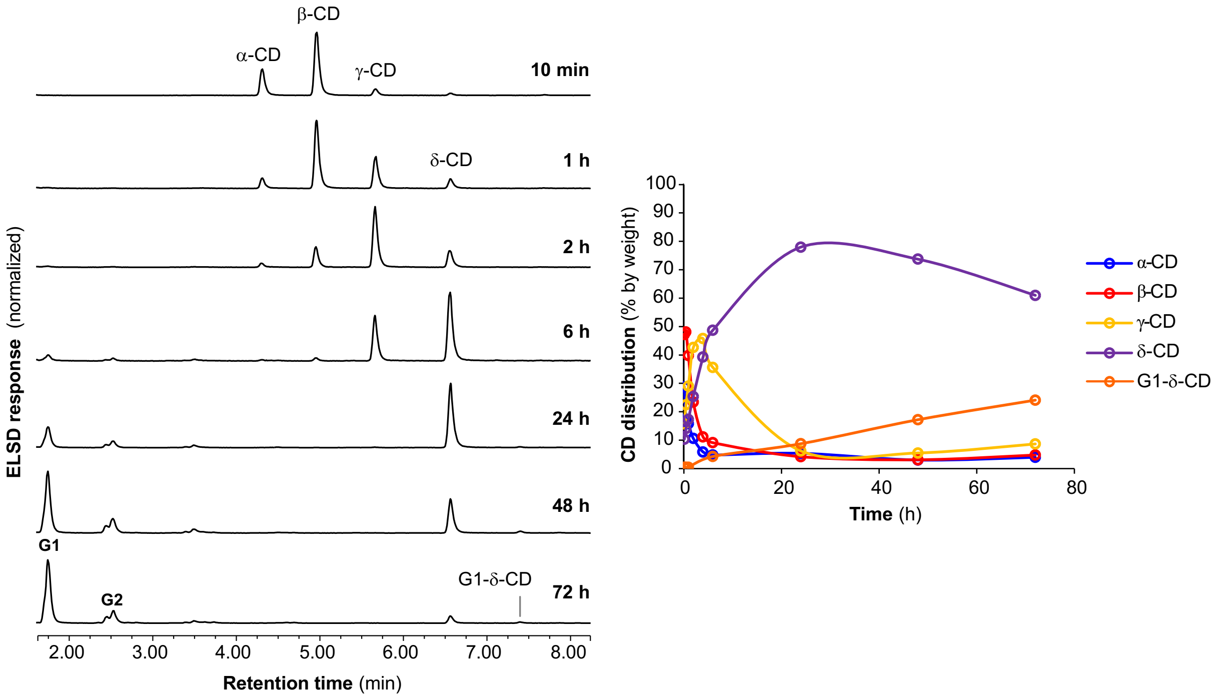

Figure S15. Reaction monitoring of the CGTase-mediated reaction with starch (10 mg/ml) in the presence of $Na_2B_{12}Cl_{12}$ (5 mM). *Left:* HPLC-ELSD chromatograms from the reaction. *Right:* Distribution within CD subsystem (α-, β-, γ-, δ-CD and G1-δ-CD). See section S7 for isolation and identification of G1-δ-CD. The peaks corresponding to linear α-1,4-glucans **G2**–**G6** could not be accurately integrated due to overlap with peaks presumably corresponding to branched non-cyclic maltodextrins containing both α-1,4-, and α-1,6-linkages, so the full glucan distribution was not plotted.

S8.5 CGTase-, isoamylase-, and pullulanase-mediated reactions with starch as starting material and $Na_2B_{12}Cl_{12}$ as template

A stock solution of soluble starch (15 mg/ml) in sodium phosphate buffer (50 mM, pH 6.0) was prepared by heating starch and buffer in the microwave (8 heating cycles with vigorous mixing between the cycles) until dissolution. Starch stock solution, a stock solution of $Na_2B_{12}Cl_{12}$ (50 mM) in sodium phosphate buffer (50 mM, pH 6.0) and sodium phosphate buffer (50 mM, pH 6.0) were mixed with spin-filtered CGTase stock solution, isoamylase (4 µl of a 1800000 U/ml stock solution prepared by 26 -fold dilution with buffer of an 46.8 MU/ml commercial stock) and pullulanase (2 µl of a 650 U/ml stock) to give a total reaction volume of 200 µl and final concentrations of starch (10 mg/ml), $Na_2B_{12}Cl_{12}$ (0 mM or 5 mM) and CGTase (65 µl stock per ml reaction mixture). The reaction was kept at room temperature and monitored at various time points. Aliquots for analysis (3 µl) were removed and the enzymatic reaction was stopped by immediate addition to a quenching mixture (90 µl) consisting of 1% trifluoroacetic acid in acetonitrile/water (3:1) with ammonium chloride (10 mM). The samples were centrifuged (8 min, 10000 rpm, 6708 RCF) and the top 83 µl fractions were transferred to HPLC vials for analysis.

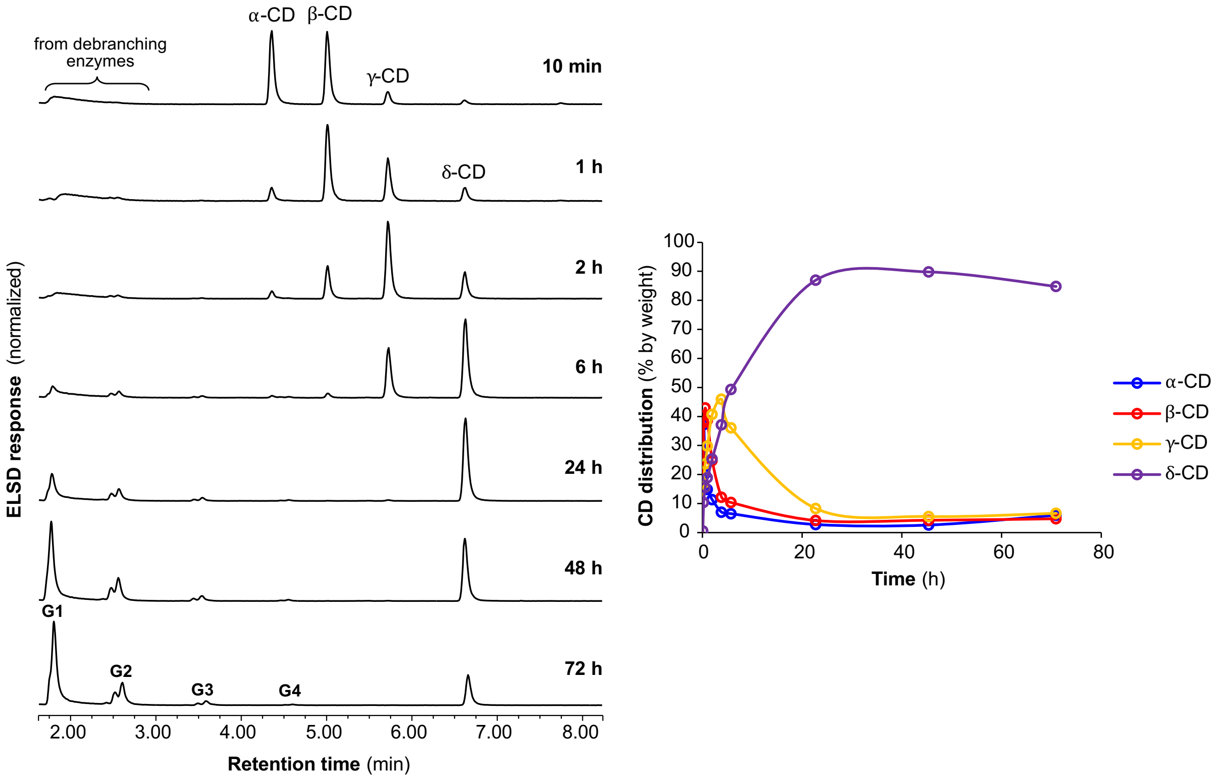

Figure S16. Reaction monitoring of the treatment of starch (10 mg/ml) with CGTase, isoamylase, and pullulanase in the presence of $Na_2B_{12}Cl_{12}$ (5 mM). *Left:* HPLC-ELSD chromatograms from the reaction. *Right*: Distribution within CD subsystem (α-, β-, γ-, δ-CD). The addition of debranching enzymes led to the presence of a broad peak overlapping with the peaks of **G1** and **G2**. Integrals for the peaks corresponding to **G1** and **G2** could not be accurately determined, so the full glucan distribution was not plotted.

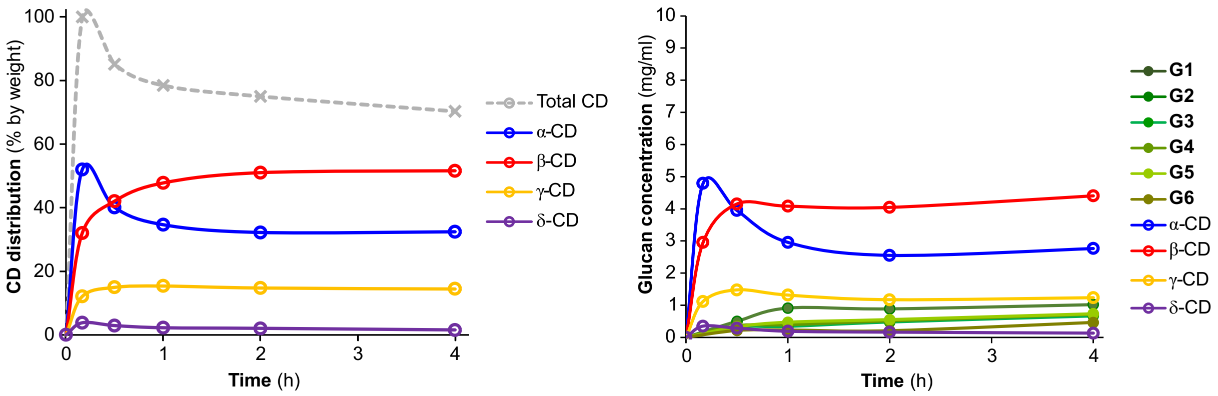

Figure S17. Reaction monitoring of the treatment of starch (10 mg/ml) with CGTase, isoamylase, and pullulanase in the absence of template. *Left*: Distribution within CD subsystem (α-, β-, γ-, δ-CD) (% by weight) as a function of time. *Right*: Distribution of all glucans in the reaction mixture (by weight) as a function of time. The addition of the debranching enzymes led to the presence of a broad peak overlapping with the peaks of **G1** and **G2**. Integrals of **G1** and **G2** were estimated by using the earliest point (10 min) as a 'baseline', which was subtracted from the integrals of later time points.

S9 NMR spectroscopy titrations of CDs (α-, β-, γ-, δ-, ε-CD) with $Na_2B_{12}X_{12}$ (X = Cl, Br, I)

In all NMR titrations, the sodium salt of the dodecaborate ion was titrated into a solution of the cyclodextrin (α-, β-, γ-, δ- or ε-CD, typically at a specific concentration in 0.01–2 mM range). All titrations were performed in D_2O at 298 K on a 400 MHz instrument, except for the titrations of γ-CD with $Na_2B_{12}Br_{12}$, δ-CD with $Na_2B_{12}Cl_{12}$, $Na_2B_{12}Br_{12}$, and $Na_2B_{12}I_{12}$, and ε-CD with $Na_2B_{12}I_{12}$, which were performed at 800 MHz. The results from the titrations were analyzed and fitted to either a 1:1 or a 2:1 binding model.

S9.1 α-CD and $Na_2B_{12}Cl_{12}$

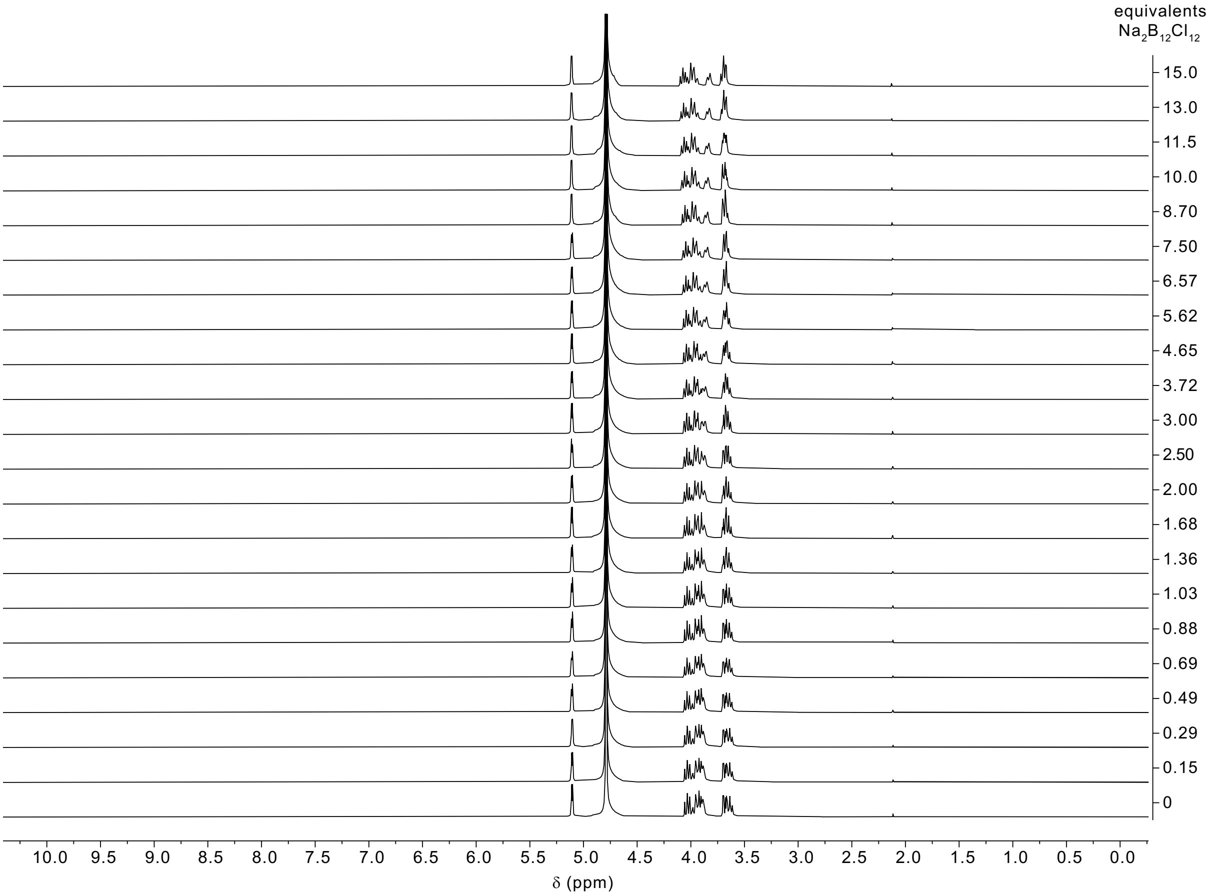

Figure S18. ^{1}H NMR spectra (400 MHz) obtained during the titration of α-CD (2 mM) with increasing equivalents of $Na_2B_{12}Cl_{12}$ in D_2O.

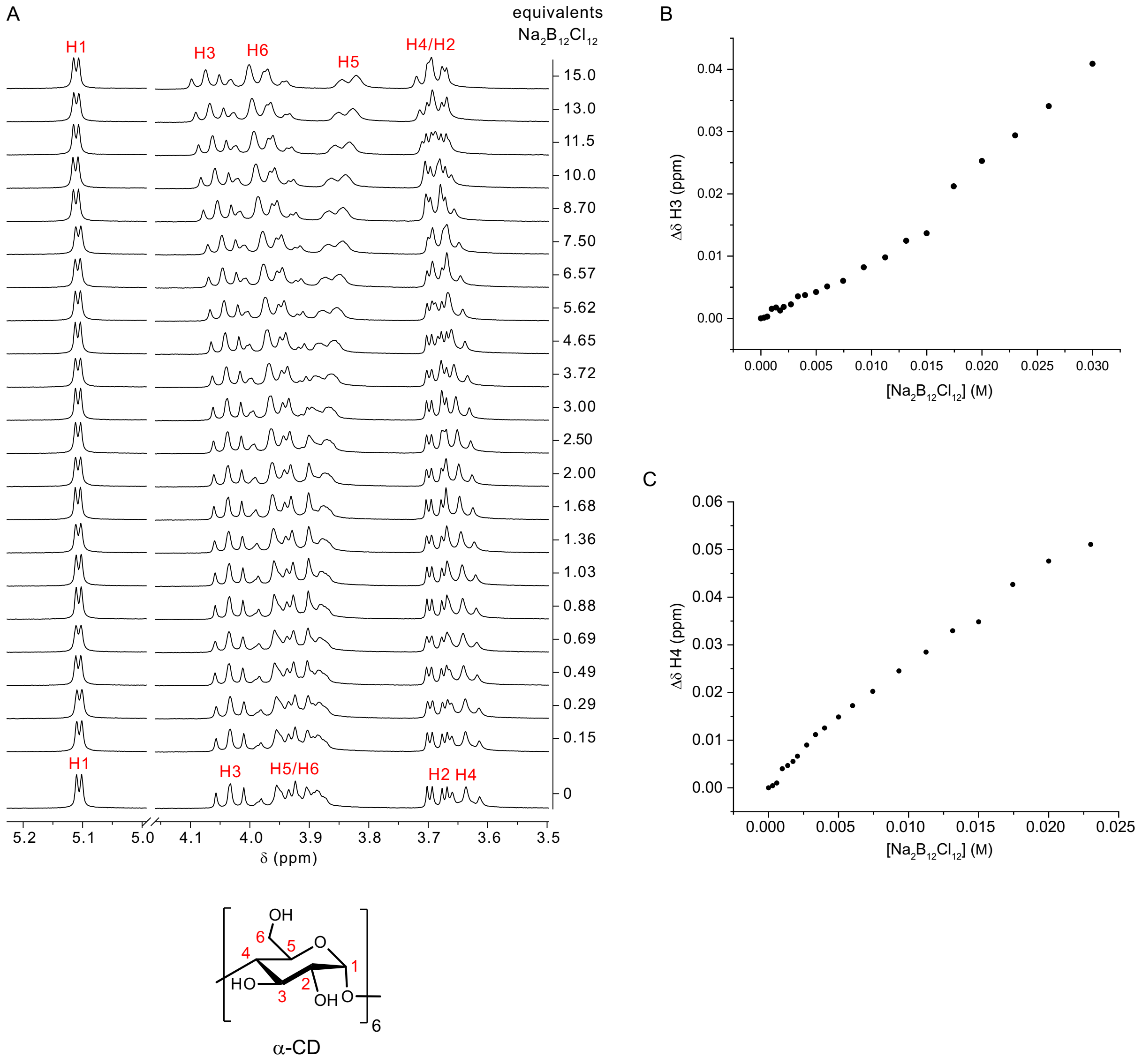

Figure S19. NMR spectroscopy titration of α-CD (2 mM) with $Na_2B_{12}Cl_{12}$ in D_2O. (A) Partial ^{1}H NMR (400 MHz) spectra of α-CD with increasing equivalents of $Na_2B_{12}Cl_{12}$. (B) Change in chemical shift (Δδ) for the H3 proton of α-CD. (C) Change in chemical shift (Δδ) for the H4 proton of α-CD.

S9.2 β-CD and $Na_2B_{12}Cl_{12}$

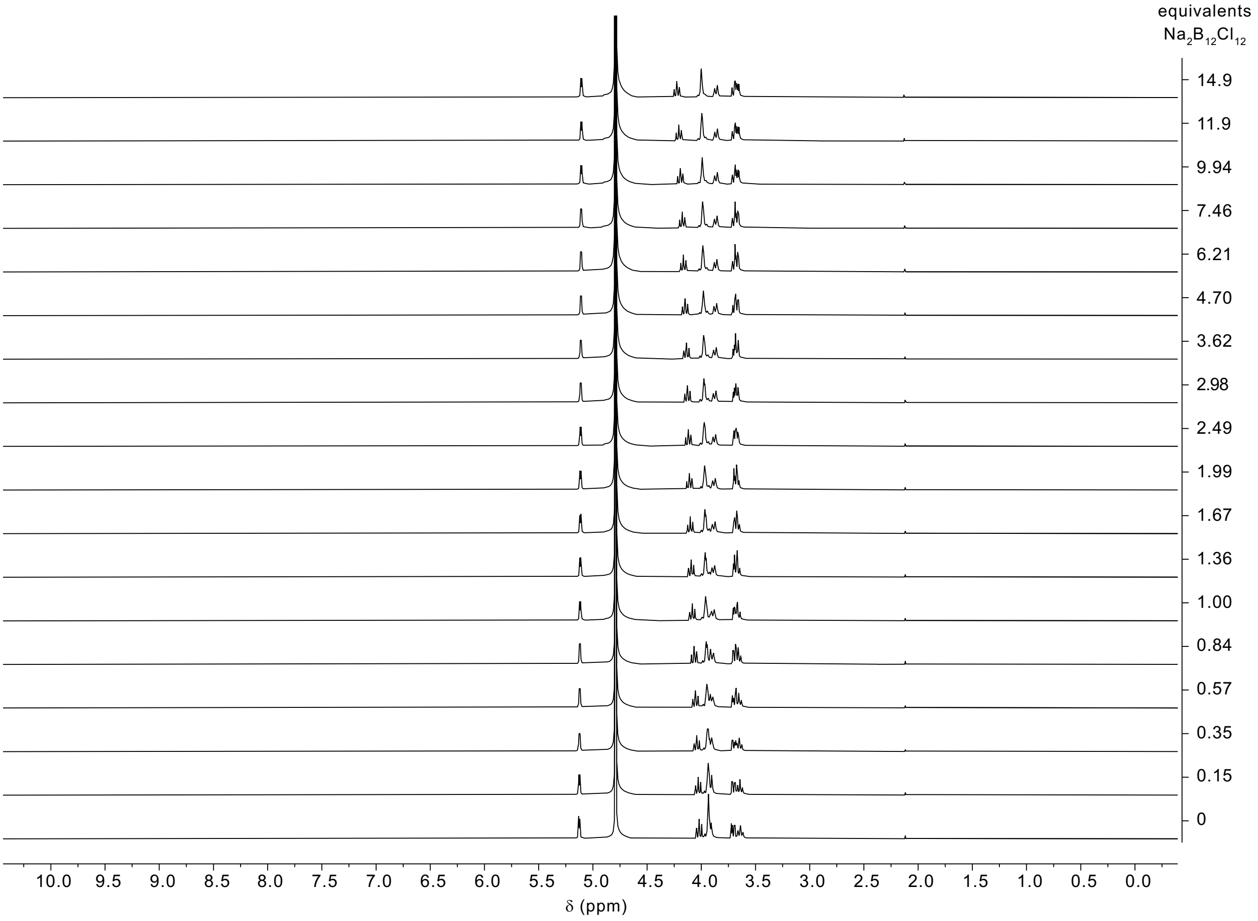

Figure S20. ^{1}H NMR spectroscopy (400 MHz) titration of β-CD (2 mM) with increasing equivalents of $Na_2B_{12}Cl_{12}$ in D_2O.

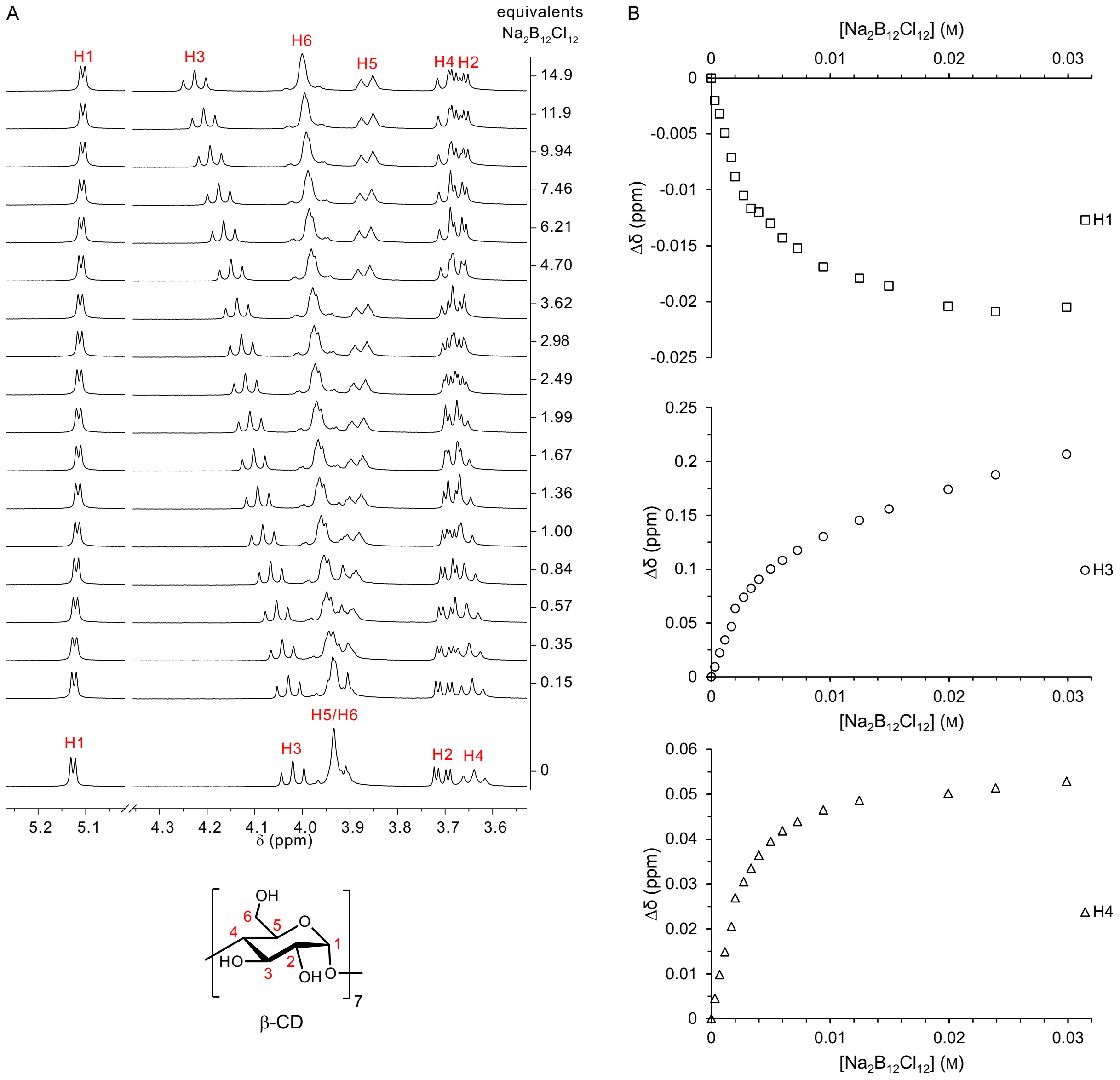

Figure S21. NMR spectroscopy titration of β-CD (2 mM) with $Na_2B_{12}Cl_{12}$ in D_2O. (A) Partial ^{1}H NMR (400 MHz) spectra of β-CD with increasing equivalents of $Na_2B_{12}Cl_{12}$. (B) Change in chemical shift (Δδ) for the H1, H3 and H4 protons of β-CD. While the Δδ for H1 and H4 could be fitted to a 1:1 binding isotherm (indicating a low association constant in the 10^2 M^{-1} range), the continuing movement of H3 indicates other processes are also taking place, and therefore no reliable association constants could be determined from this data.

S9.3 γ-CD and $Na_2B_{12}Cl_{12}$

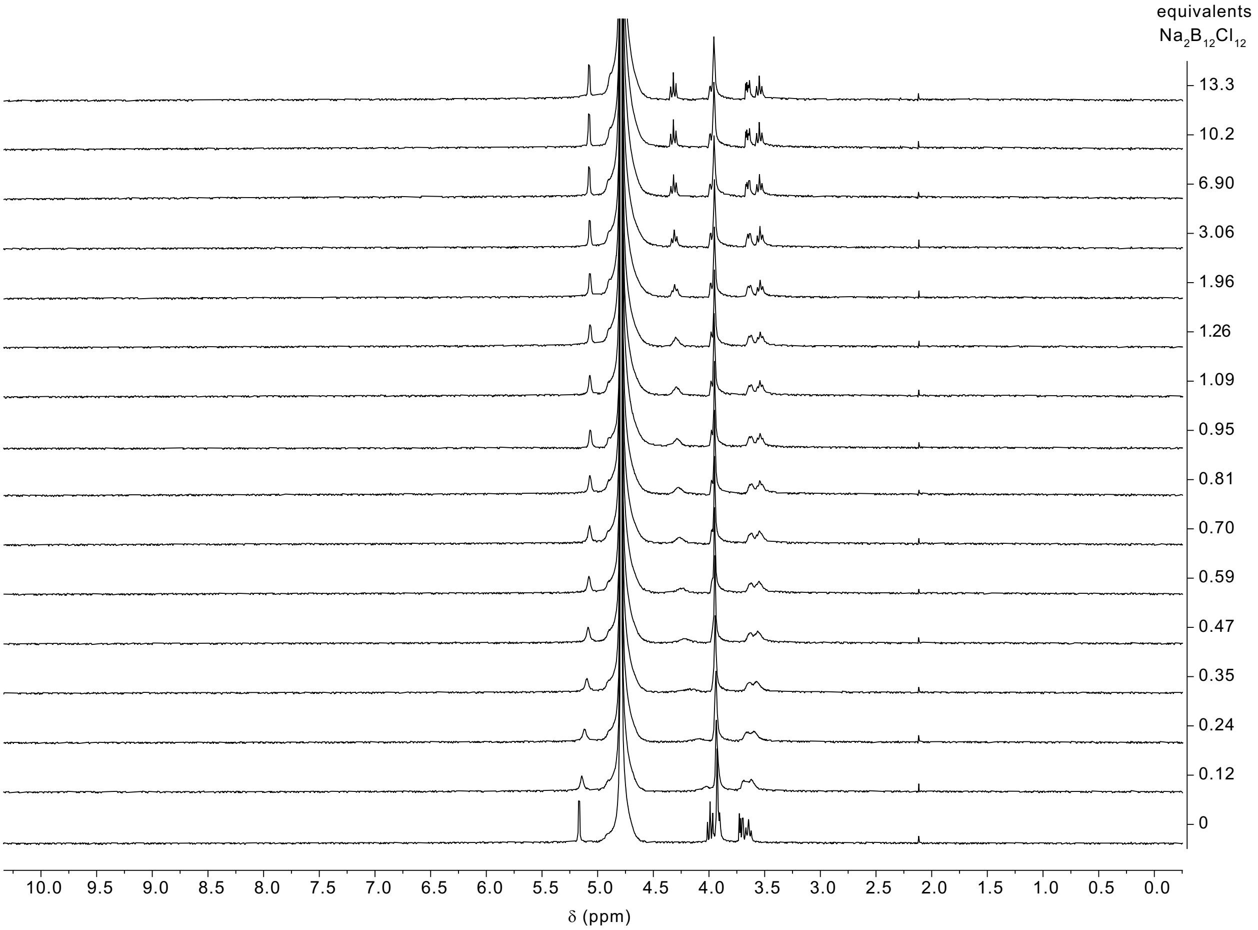

Figure S22. ^{1}H NMR spectroscopy (400 MHz) titration of γ-CD (0.3 mM) with increasing equivalents of $Na_2B_{12}Cl_{12}$ in D_2O.

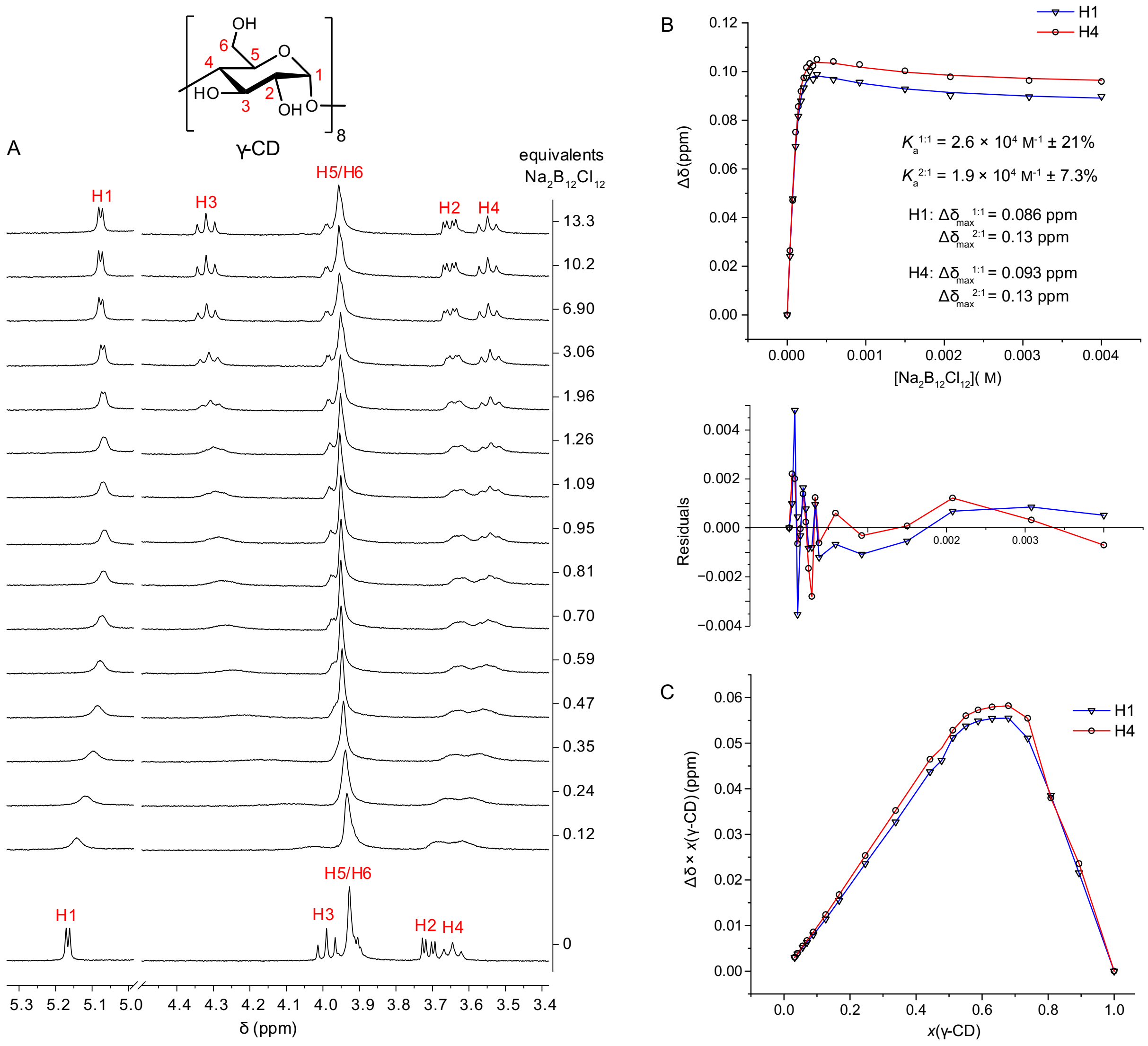

Figure S23. NMR spectroscopy titration of γ-CD (0.3 mM) with $Na_2B_{12}Cl_{12}$ in D_2O. (A) Partial ^{1}H NMR (400 MHz) spectra of γ-CD with increasing equivalents of $Na_2B_{12}Cl_{12}$. (B) Change in chemical shift (Δδ) for the H1 and H4 protons of γ-CD and the resulting fit to a 2:1 binding model. Link to data and fit: http://app.supramolecular.org/bindfit/view/ed3a3a31-20e2-4195-b879-caa95212ea9a. (C) Job plots (using the MacCarthy method)[9] of the H1 and H4 protons of γ-CD suggest 2:1 (γ-CD / $B_{12}Cl_{12}^{2-}$) binding. The mole fraction x(γ-CD) is calculated from the known concentrations of added host and guest: [γ-CD]/([γ-CD]+[$Na_2B_{12}Cl_{12}$]).

S9.4 γ-CD and $Na_2B_{12}Br_{12}$

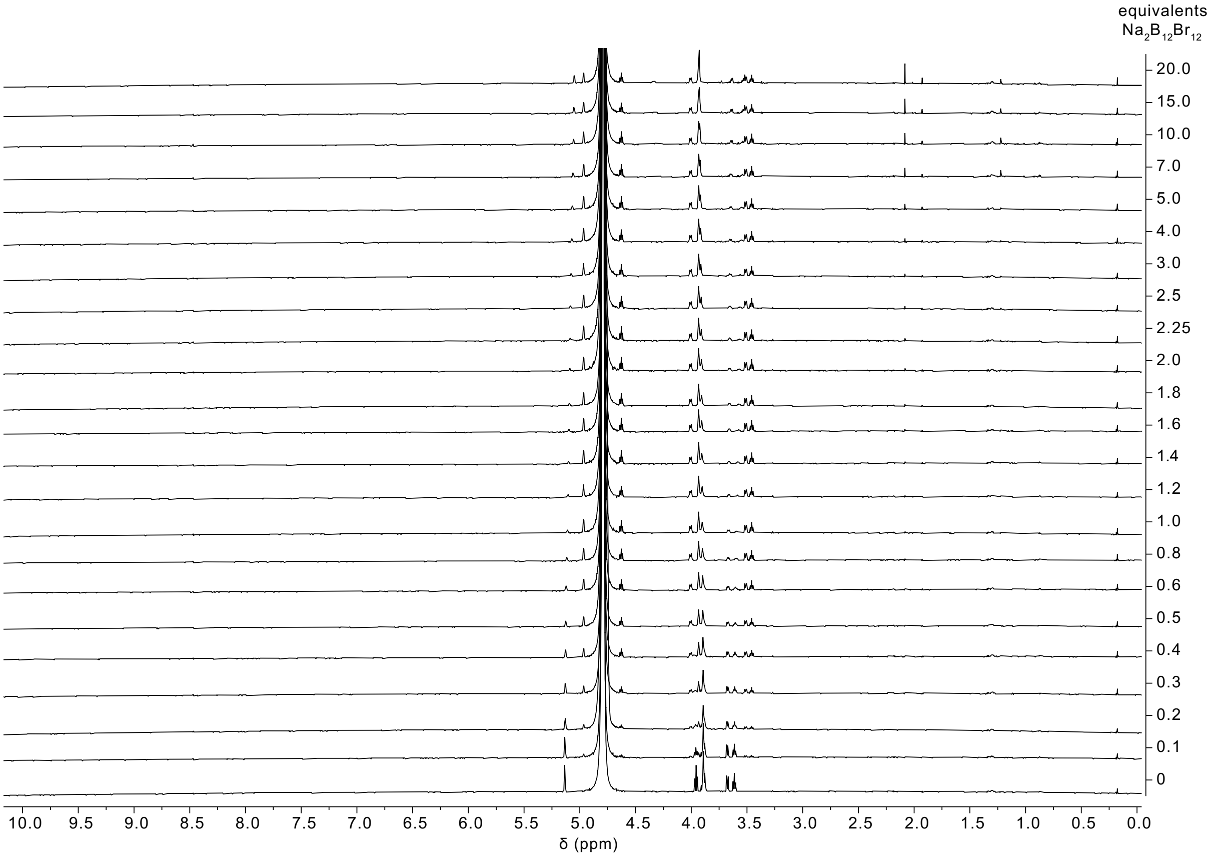

Figure S24. ^1H NMR spectroscopy (800 MHz) titration of γ-CD (0.01 mM) with increasing equivalents of $Na_2B_{12}Br_{12}$ in D_2O.

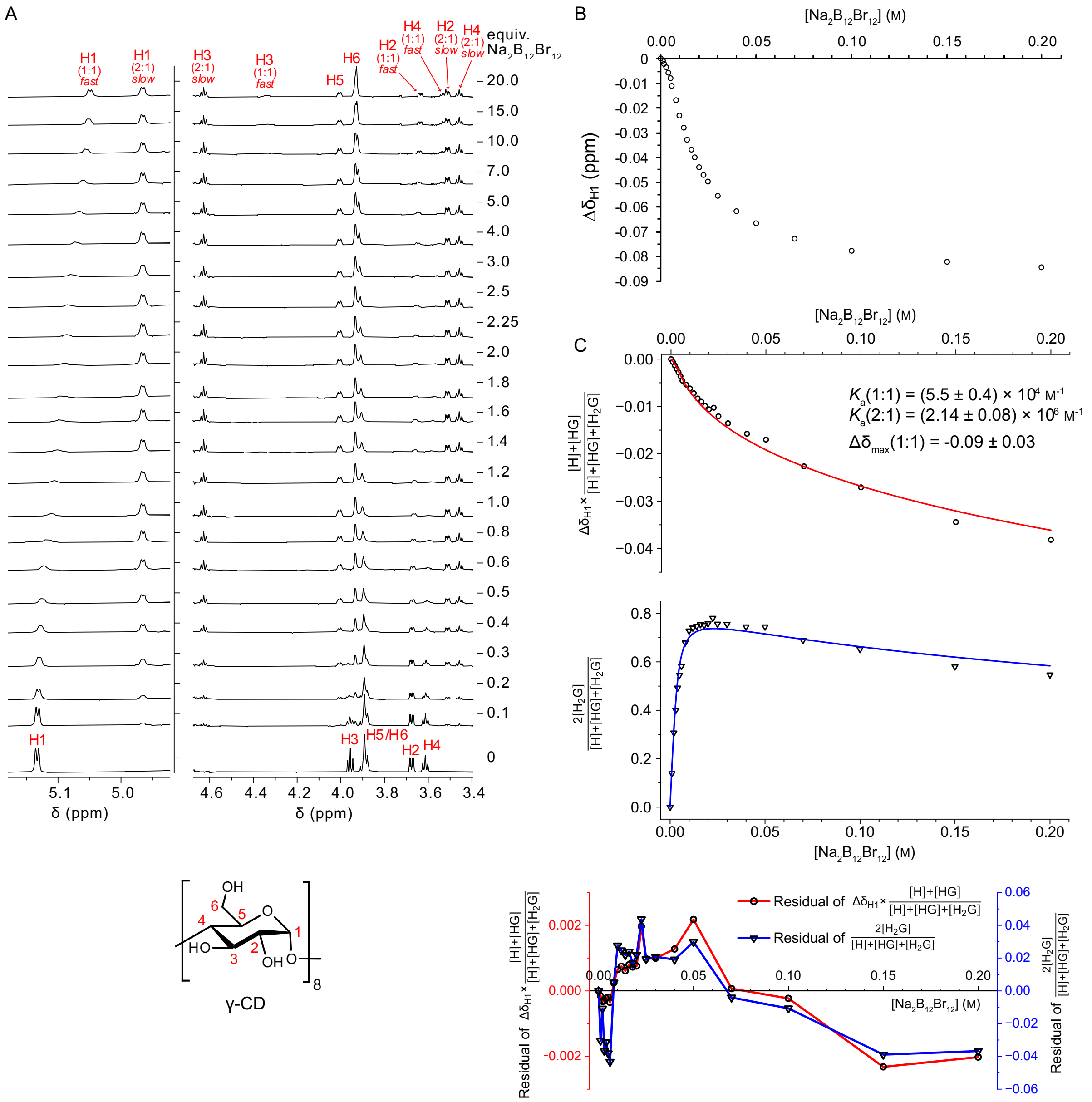

Figure S25. NMR spectroscopy titration of γ-CD (0.01 mM) with $Na_2B_{12}Br_{12}$ in D_2O. (A) Partial ^{1}H NMR (800 MHz) spectra of γ-CD with increasing equivalents of $Na_2B_{12}Br_{12}$. Note that the peaks exist both in fast exchange (γ-CD·$B_{12}Br_{12}^{2-}$ complex) and slow exchange ((γ-CD)$_2$·$B_{12}Br_{12}^{2-}$ complex) with the signals of the free γ-CD. (B) Change in chemical shift of H1 with increasing $Na_2B_{12}Br_{12}$ concentration. (C) Non-linear fits to values calculated based on the change in chemical shift of H1 and the concentrations of ([H]+[HG]) and [H_2G] as determined by integration of the two H1 peaks, as described in section S11.

S9.5 γ-CD and $Na_2B_{12}I_{12}$

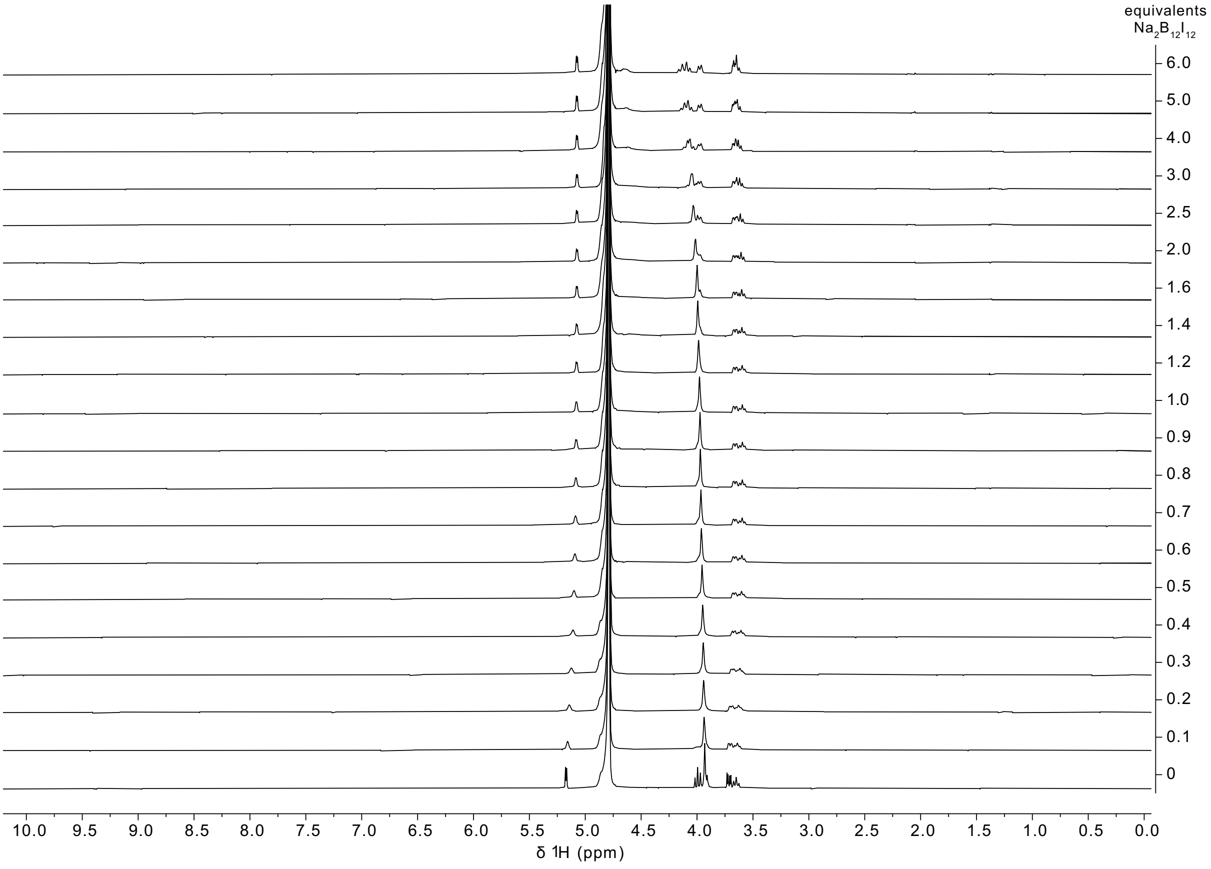

Figure S26. ^1H NMR spectroscopy (400 MHz) titration of γ-CD (0.2 mM) with increasing equivalents of $Na_2B_{12}I_{12}$ in D_2O.

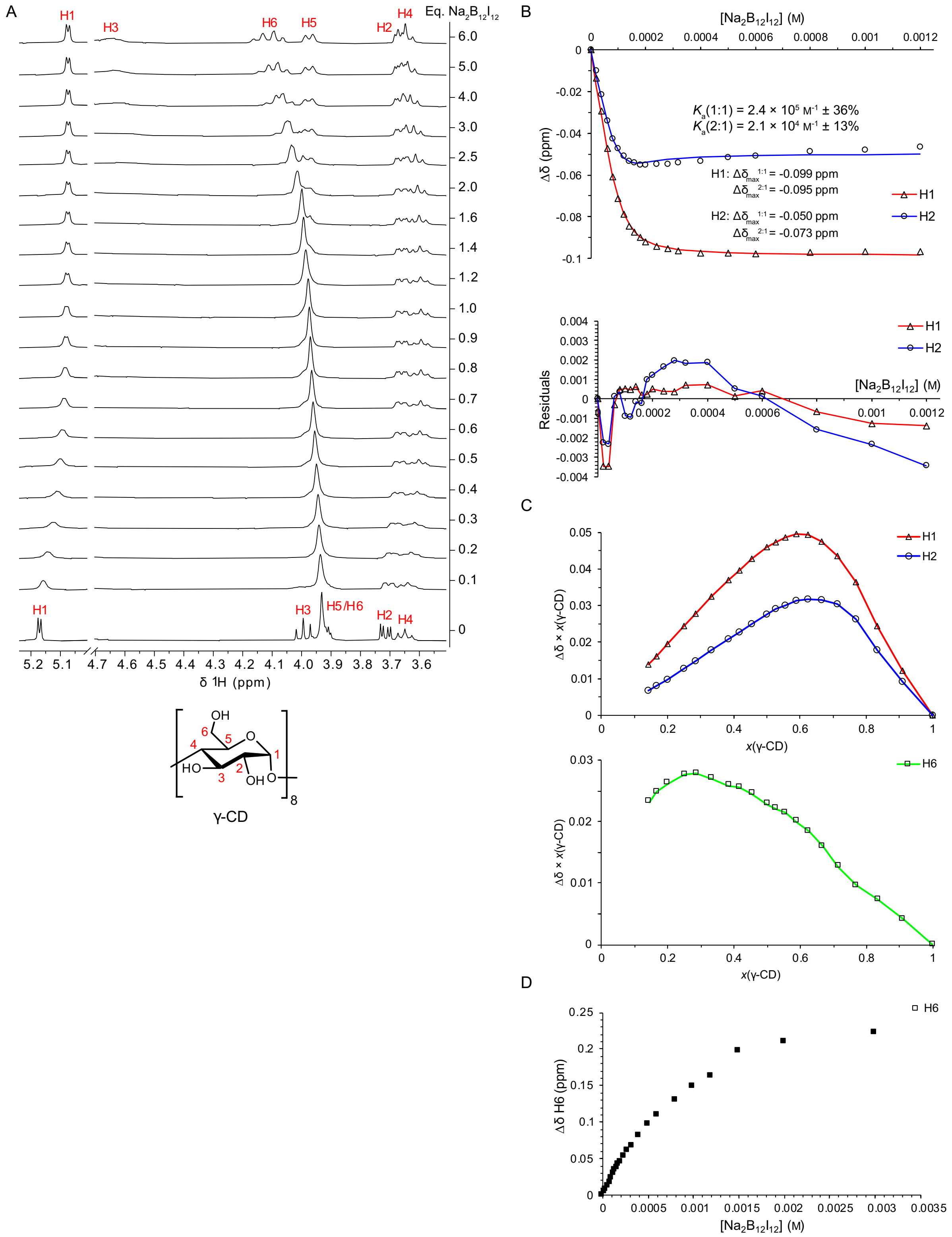

Figure S27. NMR spectroscopy titration of γ-CD (0.2 mM) with $Na_2B_{12}I_{12}$ in D_2O. (A) Partial ^{1}H NMR (400 MHz) spectra of γ-CD with increasing equivalents of $Na_2B_{12}I_{12}$. (B) Change in chemical shift (Δδ) for H1 and H2 protons of γ-CD and the resulting fit to a 2:1 binding model. Link to data and fit: http://app.supramolecular.org/bindfit/view/15d6c3e1-026d-4560-9521-0aa75444dfbf. (C) Job plots (using the MacCarthy method)[9] for H1, H2 and H6 of γ-CD. The mole fraction *x*(γ-CD) is calculated from the known concentrations of added host and guest: [γ-CD]/([γ-CD]+[$Na_2B_{12}I_{12}$]). (D) Change in chemical shift (Δδ) for H6 proton of γ-CD without fit. The continued downfield chemical shift change seen for H6 after saturation of the 1:1 complex is indicative of the formation of a weak 1:2 complex where a second $B_{12}I_{12}^{2-}$ ion binds at the narrow rim face of the γ-CD.

S9.6 δ-CD and $Na_2B_{12}Cl_{12}$

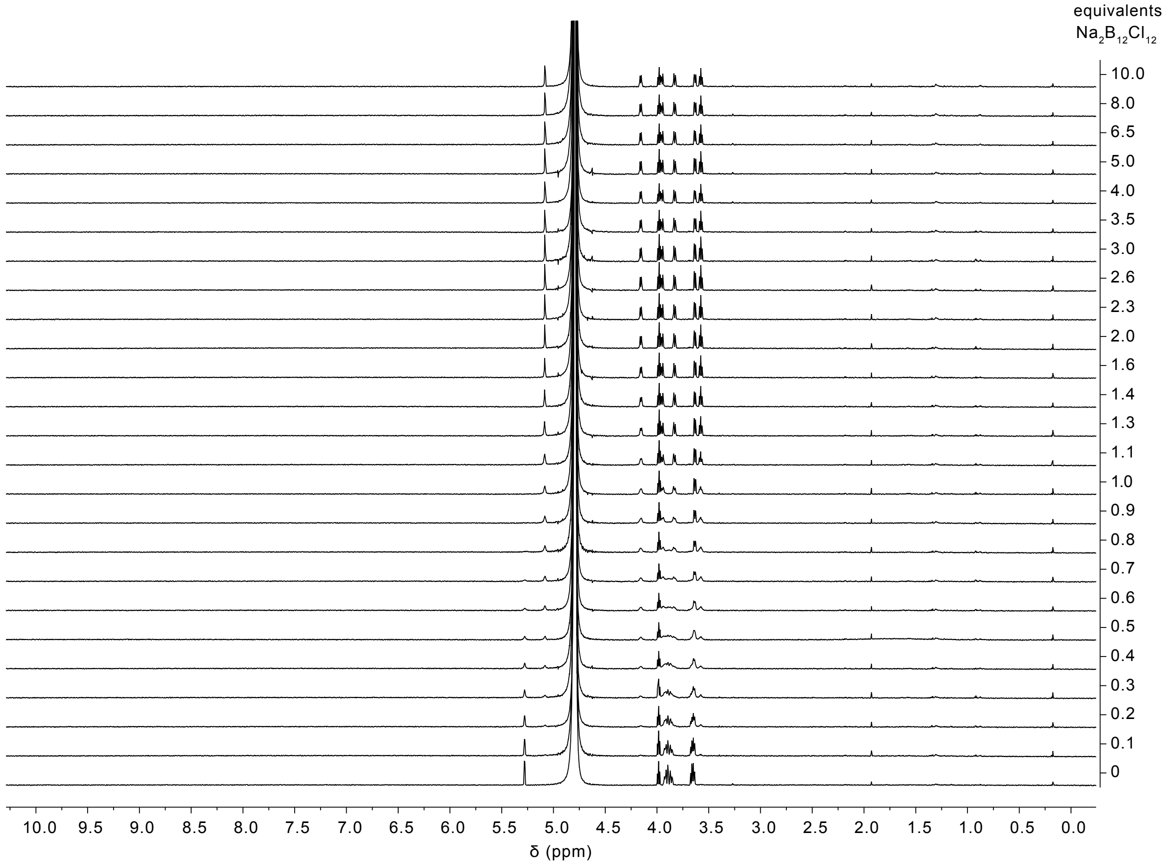

Figure S28. ^1H NMR spectroscopy (800 MHz) titration of δ-CD (0.01 mM) with increasing equivalents of $Na_2B_{12}Cl_{12}$ in D_2O.

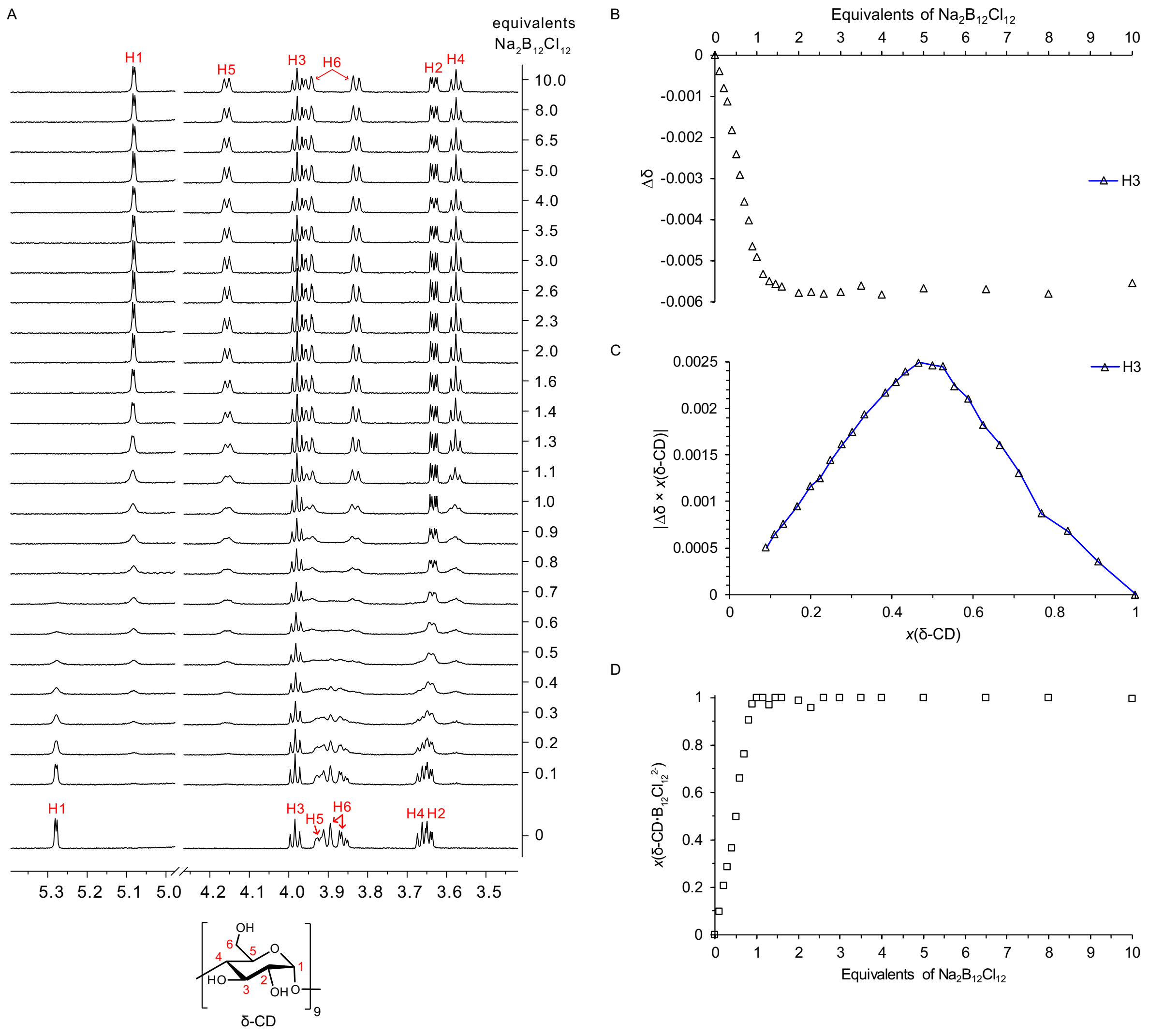

Figure S29. NMR spectroscopy titration of δ-CD (0.01 mM) with $Na_2B_{12}Cl_{12}$ in D_2O. (A) Partial ^1H NMR (800 MHz) spectra of δ-CD with increasing equivalents of $Na_2B_{12}Cl_{12}$. (B) Change in chemical shift (Δδ) for the H3 proton of δ-CD suggest a strong interaction and reaches saturation at about 1 equivalent. Performing a fit to this data suggested a binding constant in the 10^6 M^{-1} regime, but due to the small overall change in chemical shift of H3 (about 0.005 ppm), the value was found to be very dependent on careful shimming of the NMR instrument prior to data acquisition and proper referencing of the obtained data. For those reasons it was decided not to rely on these data for determination of the association constant. (C) Job plot (using the MacCarthy method)[9] for the H3 proton of δ-CD. The mole fraction *x*(δ-CD) is calculated from the known concentrations of added host and guest: [δ-CD]/([δ-CD]+[$Na_2B_{12}Cl_{12}$]). (D) Mole fraction *x*(δ-CD·$B_{12}Cl_{12}^{2-}$) calculated from the integrals of the H1 peaks (assuming formation of a 1:1 complex) as a function of added $Na_2B_{12}Cl_{12}$ indicates a strong interaction that reaches saturation at 1 equivalent of added guest. The uncertainties associated with integration of the widened H1 peaks prevent determination of the association constant with an acceptable accuracy. The association constant reported in the manuscript was determined using ITC (see section S12).

S9.7 δ-CD and $Na_2B_{12}Br_{12}$

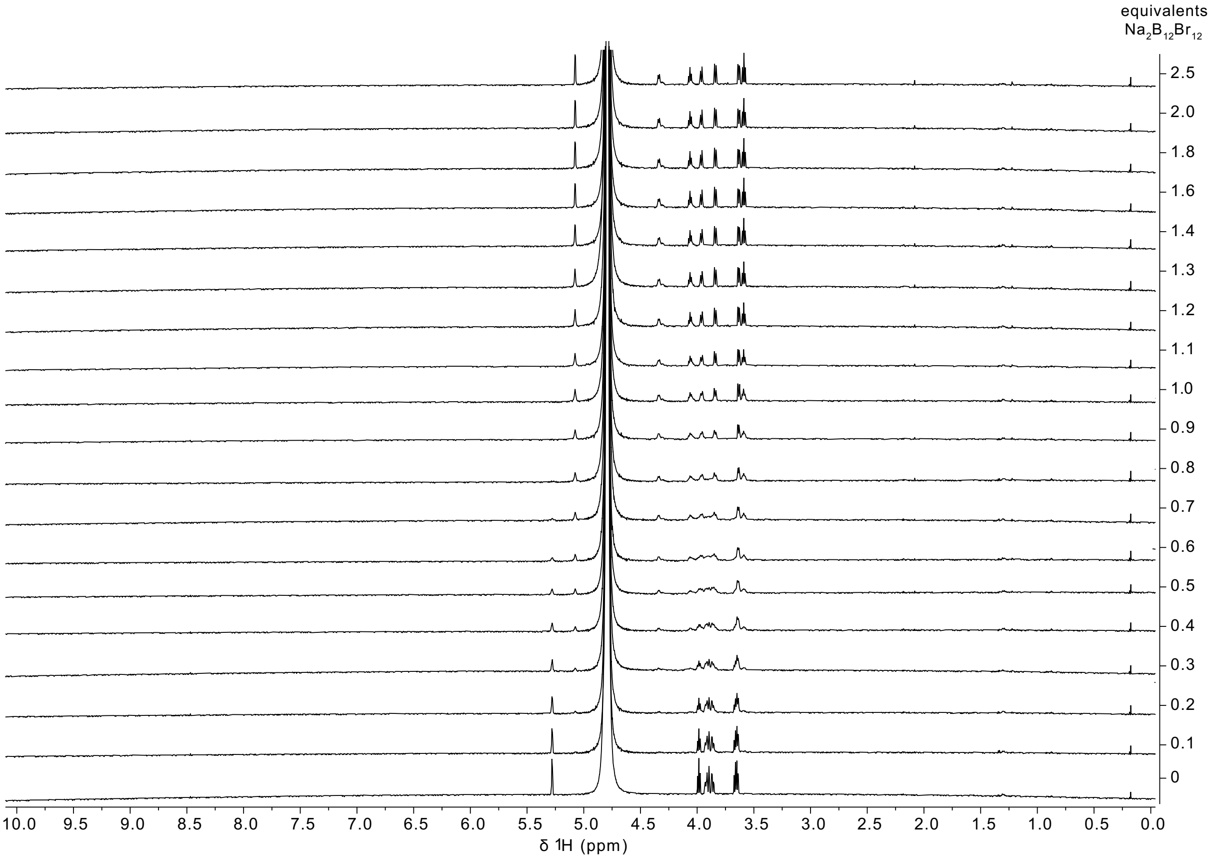

Figure S30. ^{1}H NMR spectroscopy (800 MHz) titration of δ-CD (0.01 mM) with increasing equivalents of $Na_2B_{12}Br_{12}$ in D_2O.

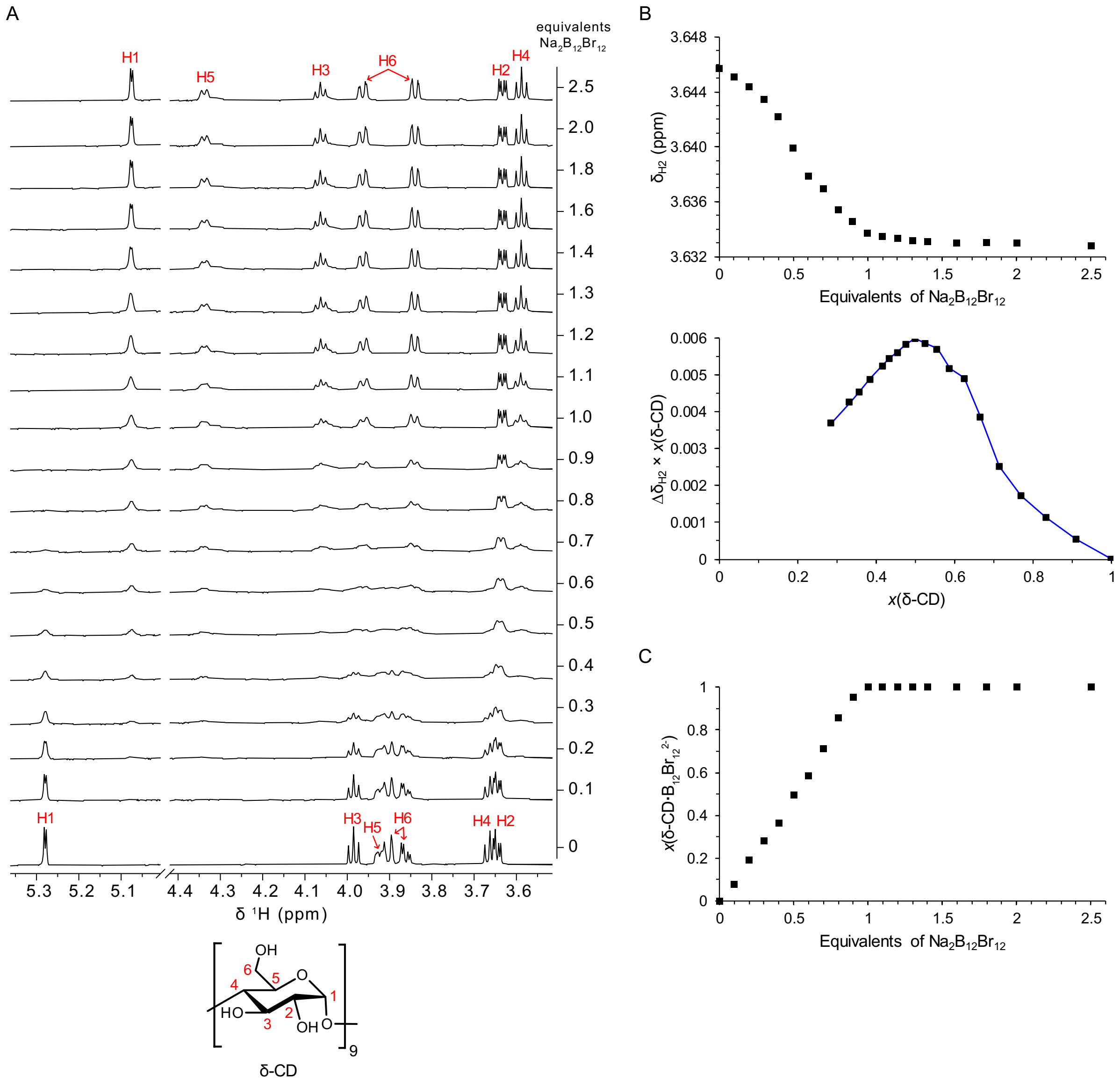

Figure S31. NMR spectroscopy titration of δ-CD (0.01 mM) with $Na_2B_{12}Br_{12}$ in D_2O. (A) Partial ^{1}H NMR (800 MHz) spectra of δ-CD with increasing equivalents of $Na_2B_{12}Br_{12}$. (B) Observed chemical shift of H2 with increasing $Na_2B_{12}Br_{12}$ concentration and resulting Job plot (using the MacCarthy method).[9] The mole fraction x(δ-CD) is calculated from the known concentrations of added host and guest: [δ-CD]/([δ-CD]+[$Na_2B_{12}Br_{12}$]). This data could not produce reasonable fits with 1:1, 1:2, or 2:1 binding models, and it is assessed that the curvature in the plot of the change in chemical shift of H2 is caused by difficulties in monitoring this peak due to the peak becoming broad during titration while also overlapping with the signal from H4, especially in early points in the titration. (C) Mole fraction $x(\delta\text{-CD}\cdot B_{12}Br_{12}^{2-})$ calculated from the integrals of the H1 peaks (assuming formation of a 1:1 complex) as a function of added $Na_2B_{12}Br_{12}$ indicates a strong interaction that reaches saturation at 1 equivalent of added guest. The uncertainties associated with integration of the widened H1 peaks prevent determination of the association constant with an acceptable accuracy (focusing on the data with 0.3 to 0.7 eq. of guest gave K_a values of (1.4 ± 13.8) × 10^6 M^{-1} (average value ± one standard deviation)). The association constant reported in the manuscript was determined using ITC (see section S12).

S9.8 δ-CD and $Na_2B_{12}I_{12}$

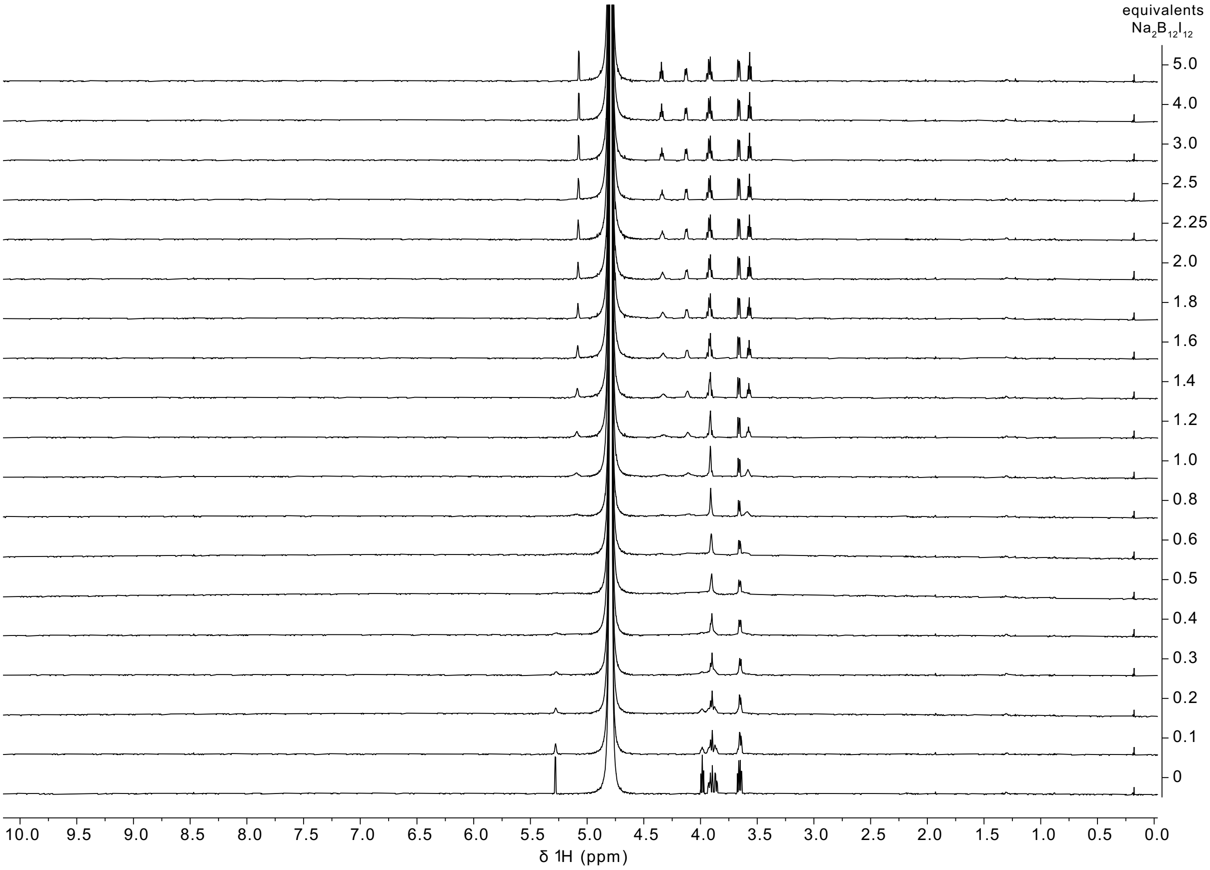

Figure S32. ^{1}H NMR spectroscopy (800 MHz) titration of δ-CD (0.01 mM) with increasing equivalents of $Na_2B_{12}I_{12}$ in D_2O.

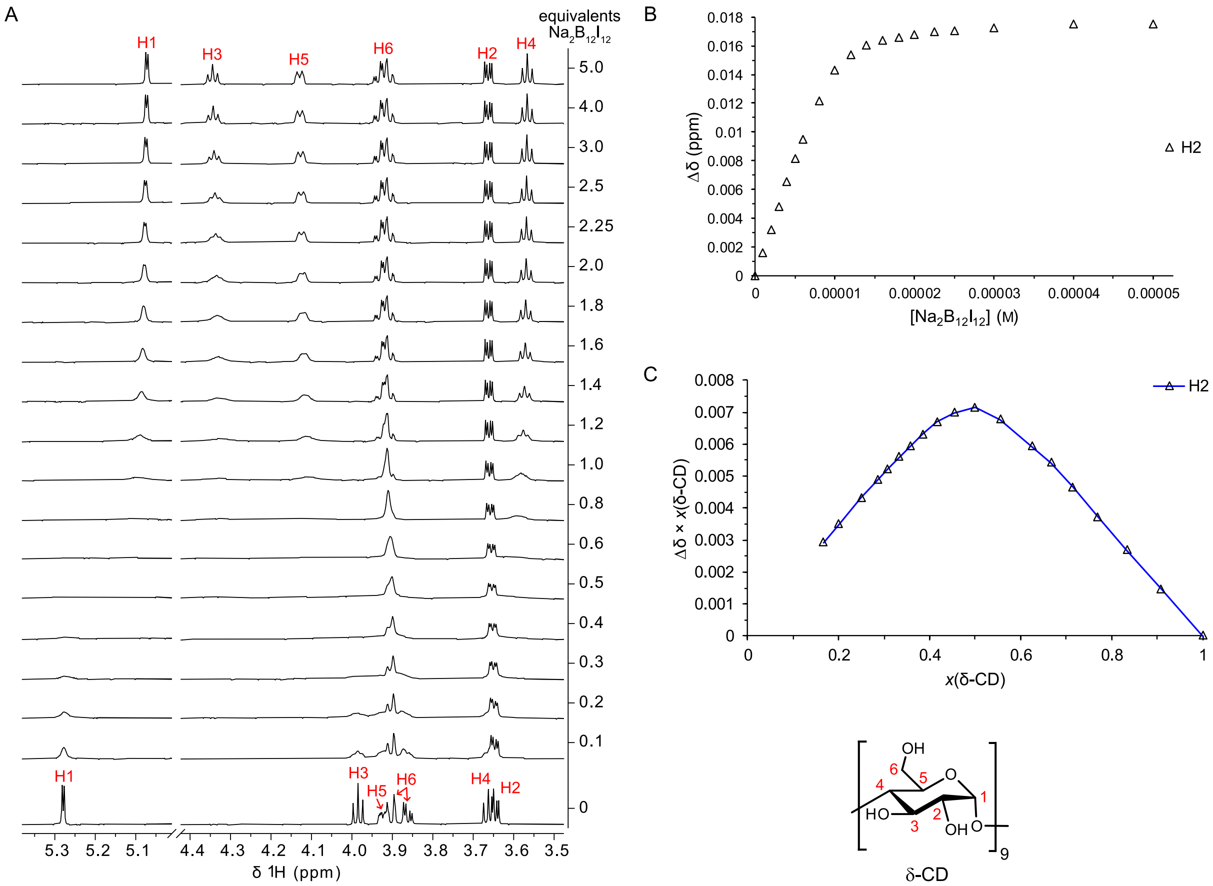

Figure S33. NMR spectroscopy titration of δ-CD (0.01 mM) with $Na_2B_{12}I_{12}$ in D_2O. (A) Partial ^{1}H NMR (800 MHz) spectra of δ-CD with increasing equivalents of $Na_2B_{12}I_{12}$. (B) Observed change in chemical shift (Δδ) of H2 of δ-CD with increasing $Na_2B_{12}I_{12}$ concentration. Note that the change in chemical shift is low (<0.02 ppm) and that all other peaks are in slow or intermediate exchange. (C) Job plot for H2 of δ-CD (using the MacCarthy method).[9] The association constant reported in the manuscript was determined using ITC (see section S12).

S9.9 ε-CD and $Na_2B_{12}Cl_{12}$

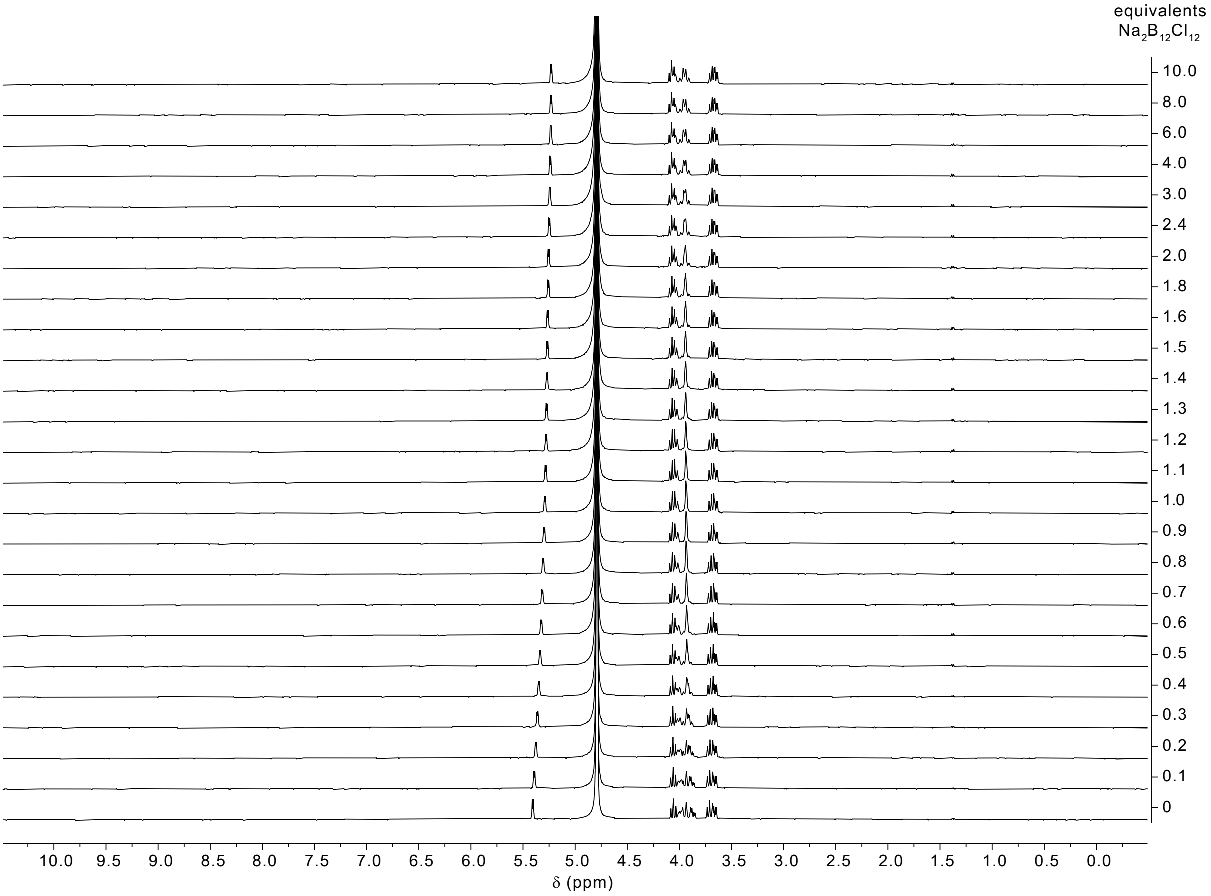

Figure S34. ^1H NMR spectroscopy (400 MHz) titration of ε-CD (0.2 mM) with increasing equivalents of $Na_2B_{12}Cl_{12}$ in D_2O.

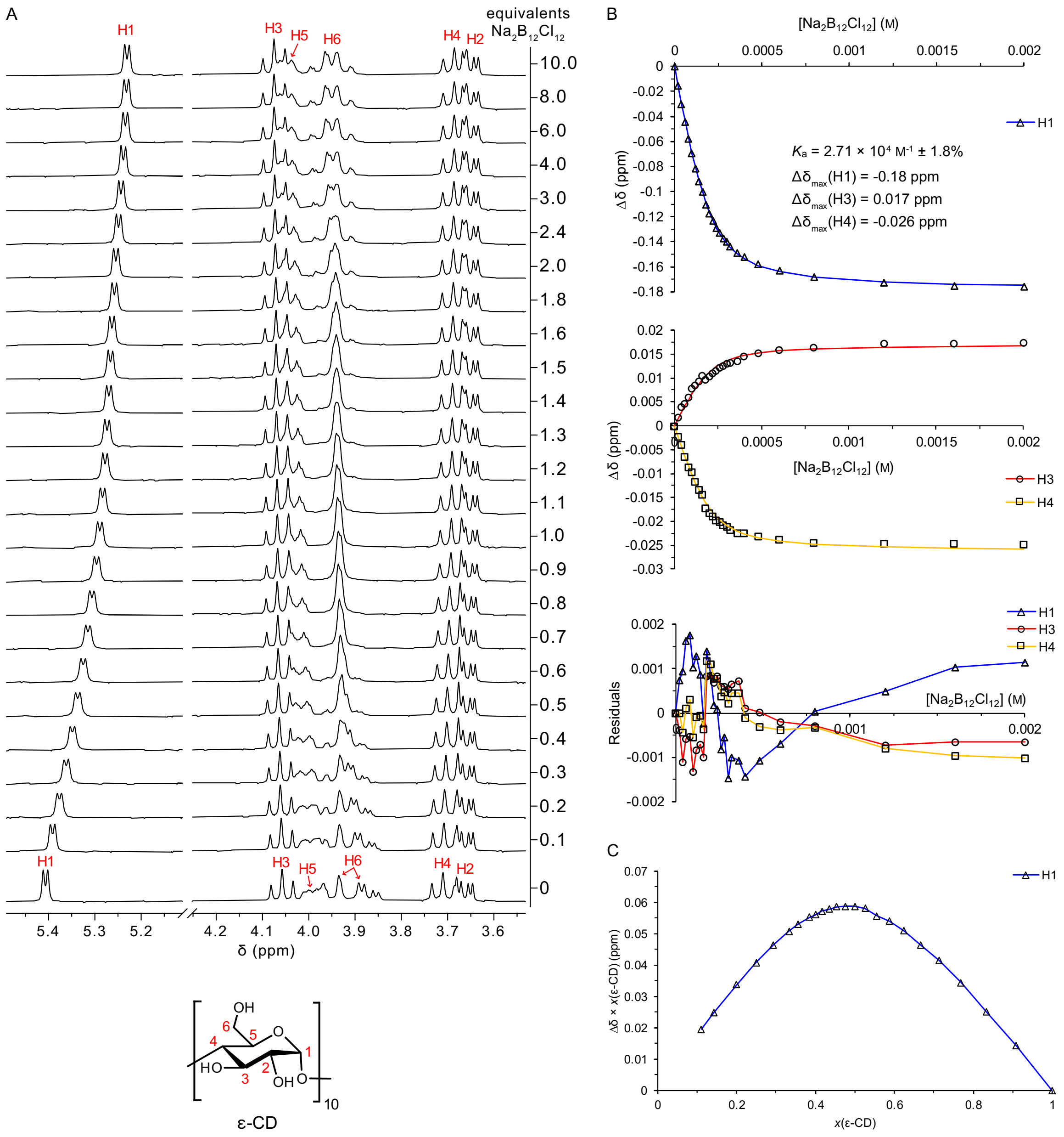

Figure S35. NMR spectroscopy titration of ε-CD (0.2 mM) with $Na_2B_{12}Cl_{12}$ in D_2O. (A) Partial ^1H NMR (400 MHz) spectra of ε-CD with increasing equivalents of $Na_2B_{12}Cl_{12}$. (B) Change in chemical shift (Δδ) for H1, H3 and H4 protons of ε-CD and the resulting fit to a 1:1 binding model. Link to data and fit: http://app.supramolecular.org/bindfit/view/efd17799-d1a4-4178-beb8-04972d2c0fb1. (C) Job plot for H1 (using the MacCarthy method).[9]

S9.10 ε-CD and $Na_2B_{12}Br_{12}$

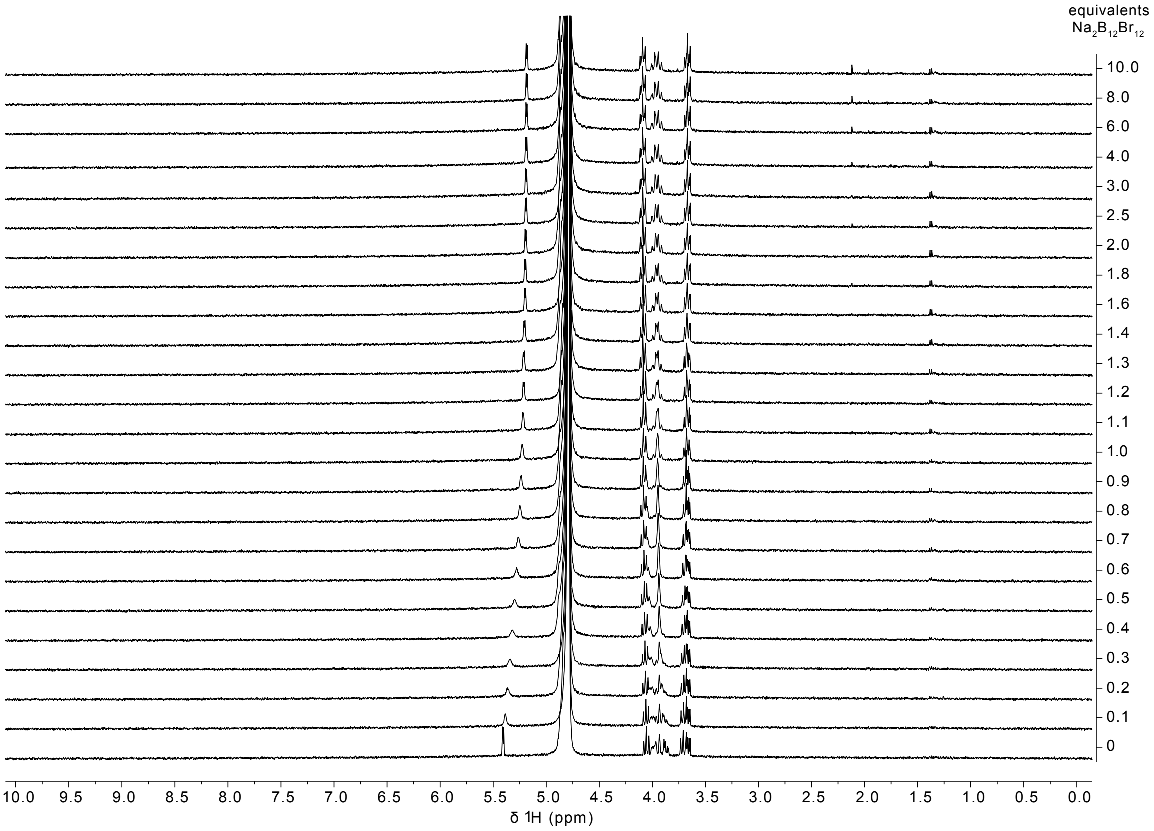

Figure S36. ^{1}H NMR spectroscopy (400 MHz) titration of ε-CD (0.1 mM) with increasing equivalents of $Na_2B_{12}Br_{12}$ in D_2O.

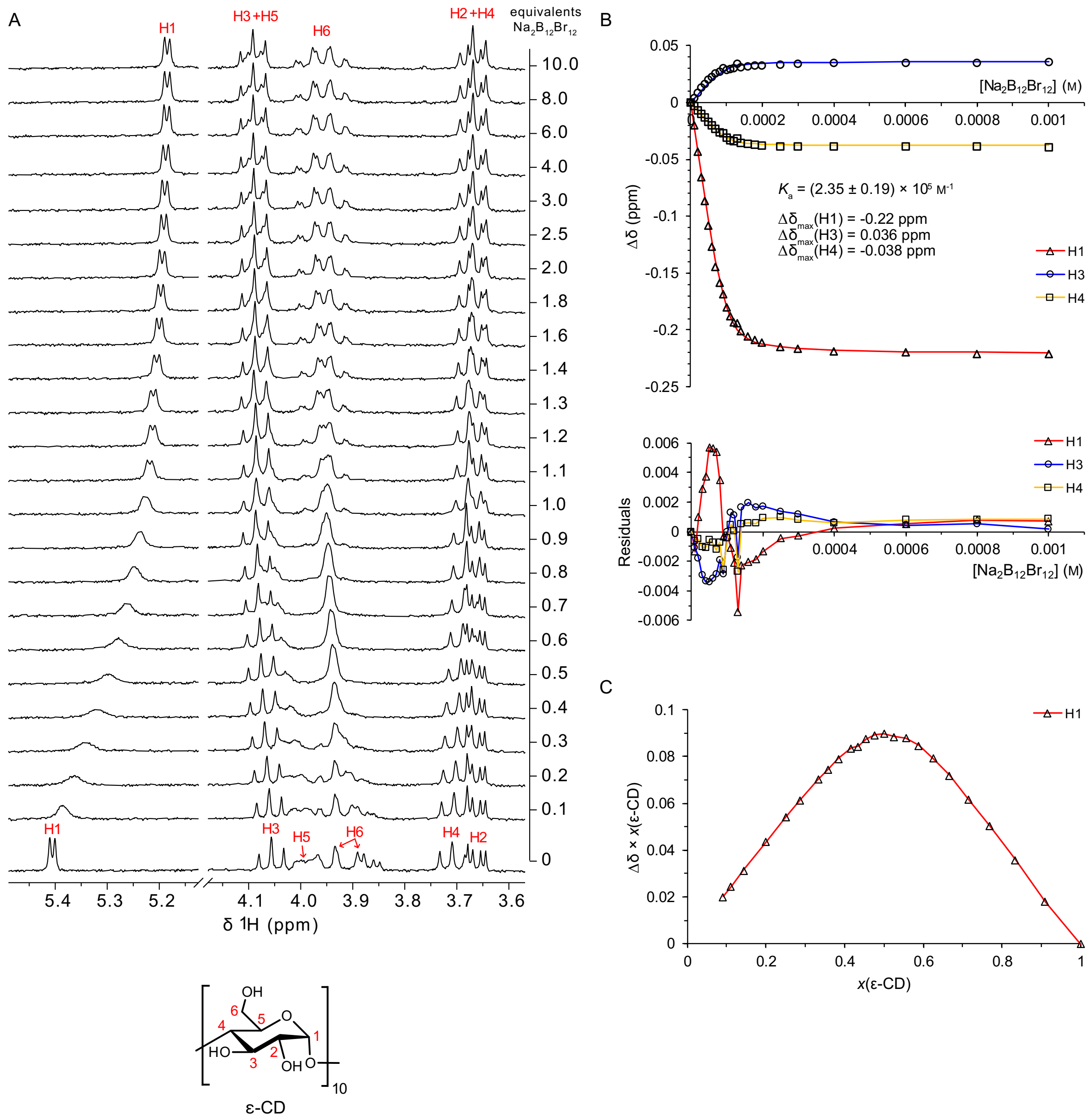

Figure S37. NMR spectroscopy titration of ε-CD (0.1 mM) with $Na_2B_{12}Br_{12}$ in D_2O. (A) Partial ^{1}H NMR (400 MHz) spectra of ε-CD with increasing equivalents of $Na_2B_{12}Br_{12}$. (B) Change in chemical shift (Δδ) for H1, H3 and H4 protons of ε-CD and the resulting fit to a 1:1 binding model. Link to data and fit: http://app.supramolecular.org/bindfit/view/72e23daf-42d1-4bf3-9140-a59b1e187428. (C) Job plot (using the MacCarthy method)[9] of H1 in ε-CD.

S9.11 ε-CD and $Na_2B_{12}I_{12}$

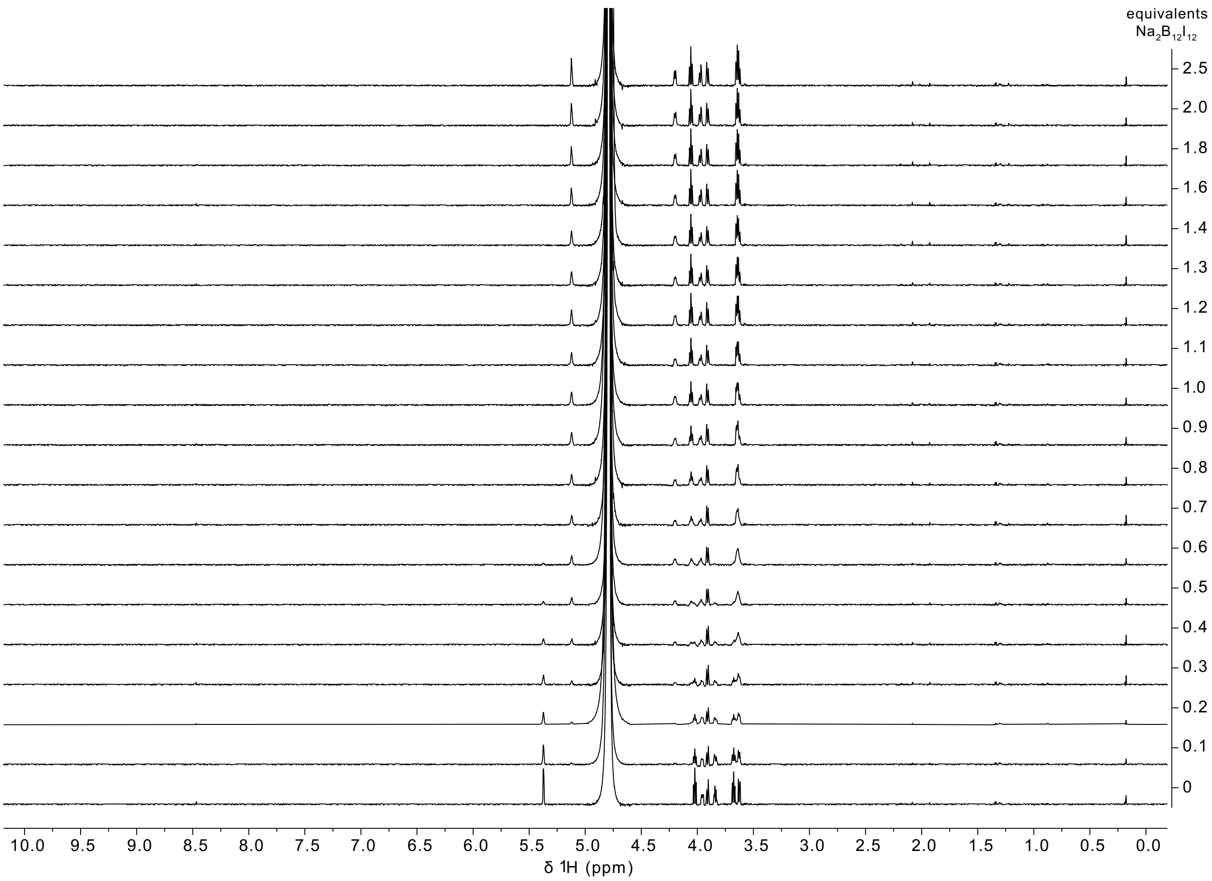

Figure S38. ^{1}H NMR spectroscopy (800 MHz) titration of ε-CD (0.01 mM) with increasing equivalents of $Na_2B_{12}I_{12}$ in D_2O.

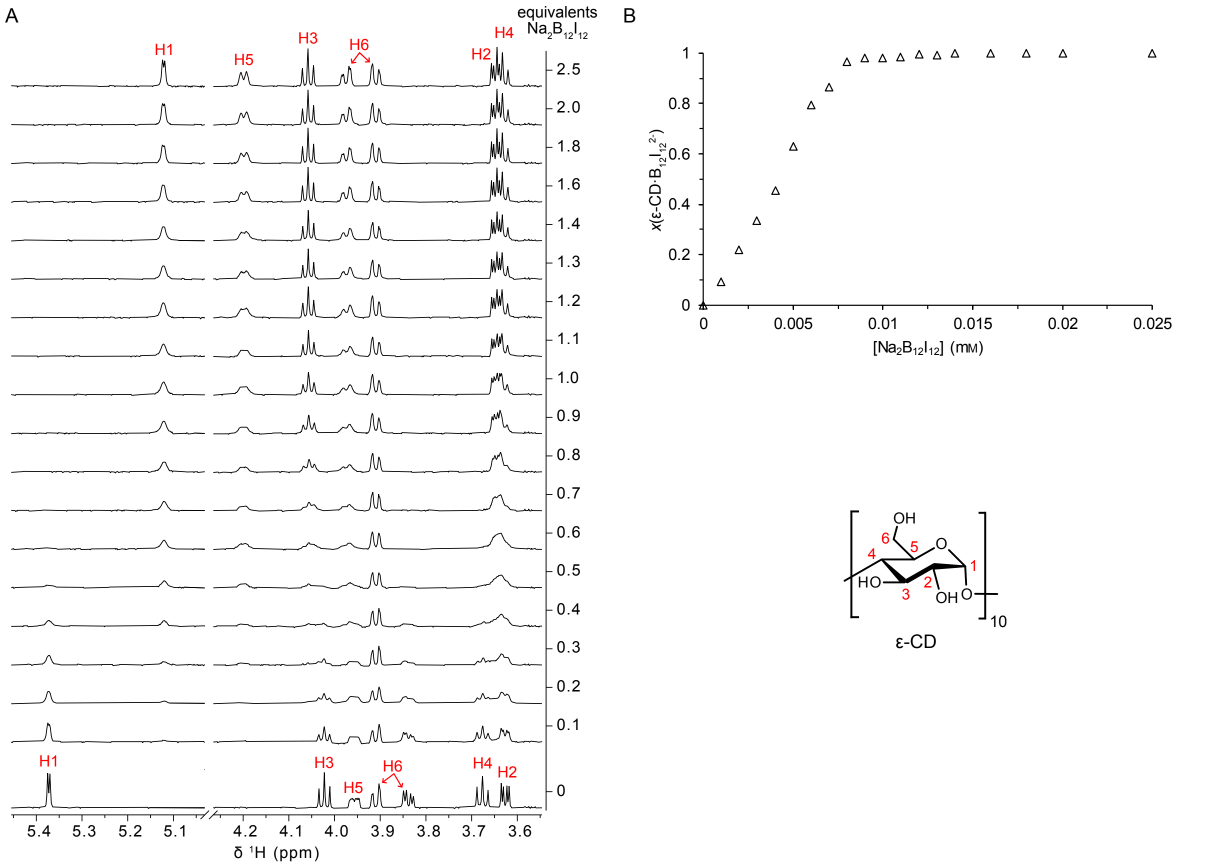

Figure S39. NMR spectroscopy titration of ε-CD (0.01 mM) with $Na_2B_{12}I_{12}$ in D_2O. (A) Partial ^1H NMR (800 MHz) spectra of ε-CD with increasing equivalents of $Na_2B_{12}I_{12}$. (B) Mole fraction $x(\delta\text{-CD}\cdot B_{12}I_{12}^{2-})$ calculated from the integrals of the H1 peaks (assuming formation of a 1:1 complex) as a function of added $Na_2B_{12}I_{12}$. The uncertainties associated with integration of the widened H1 peaks prevent determination of the association constant with an acceptable accuracy. The association constant reported in the manuscript was determined using ITC (see section S12).

S9.12 Comparison of NMR spectra for γ-CD, δ-CD and ε-CD binding $Na_2B_{12}X_{12}$

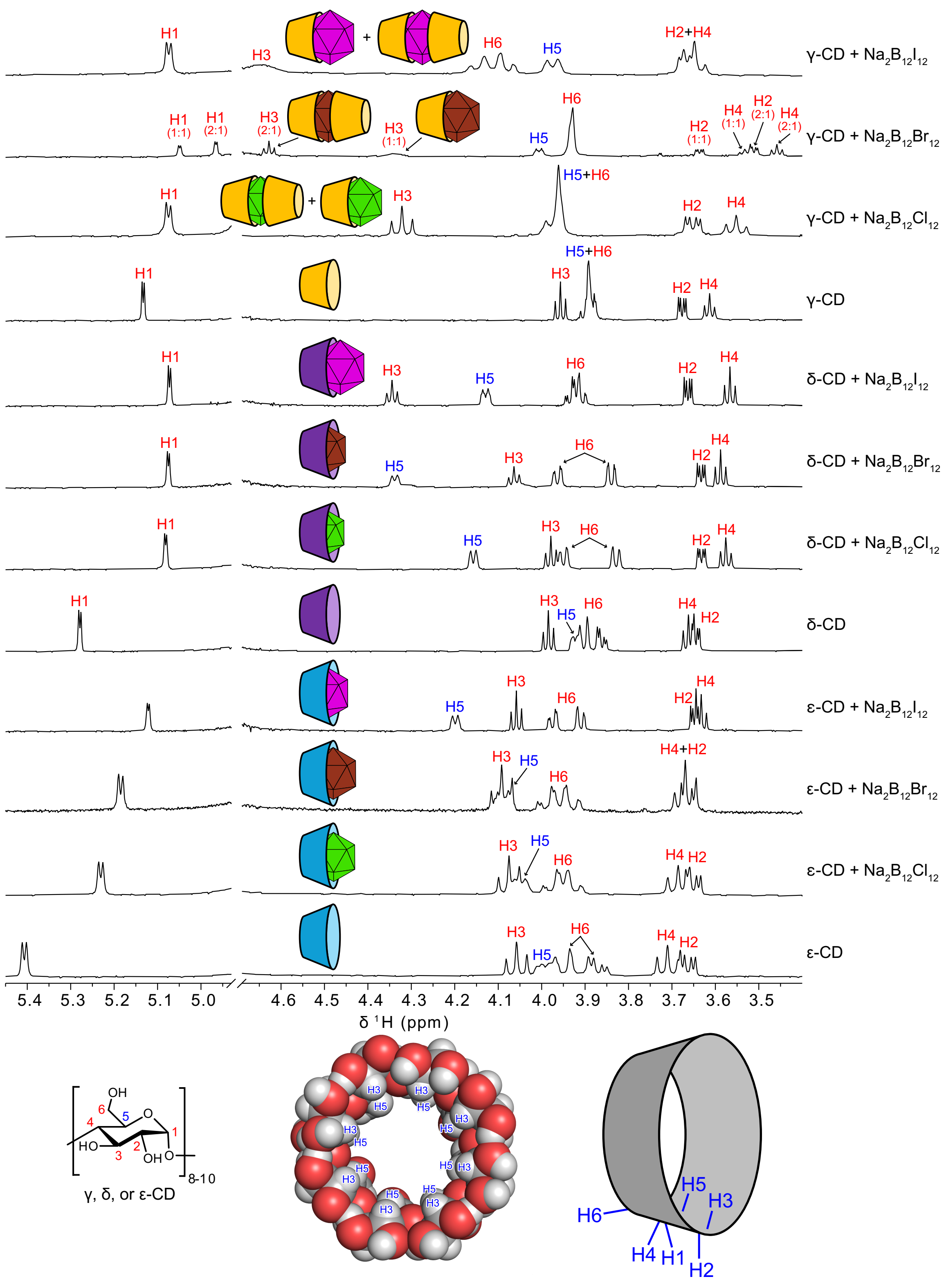

Figure S40. Comparison of the start and end points of ^{1}H NMR spectroscopy titrations of γ-, δ-, and ε-CD with $Na_2B_{12}Cl_{12}$, $Na_2B_{12}Br_{12}$ and $Na_2B_{12}I_{12}$. Note the position of the H5 and H3 protons. A downfield shift of H5 signifies deep binding within the CD cavity. A downfield shift of H3 signifies a shallow binding. The samples with γ-CD and $Na_2B_{12}I_{12}$, δ-CD and $Na_2B_{12}Cl_{12}$. ε-CD alone and with $Na_2B_{12}Br_{12}$ and $Na_2B_{12}Cl_{12}$ were obtained at 400 MHz. All other spectra were obtained at 800 MHz.

S10 DOSY NMR experiments

Diffusion-ordered spectroscopy (DOSY) experiments were performed on a 600 MHz Bruker NMR spectrometer. Samples of γ-CD were prepared at a concentration of 0.3 mM in D_2O with different template concentrations (0.3 and 3.0 mM for $Na_2B_{12}Cl_{12}$ and 3.0 mM for $Na_2B_{12}Br_{12}$). The DOSY spectra were recorded with 16 scans per 1D spectrum. A total of 16 1D spectra were acquired, with a gradient ranging from 2% to 98%. The data were processed using Bruker Topspin and DynamicsCenter, the diffusion coefficients were calculated using a standard non-linear fitting method and calibrated to the HDO signal.

DOSY with γ-CD and $Na_2B_{12}Cl_{12}$

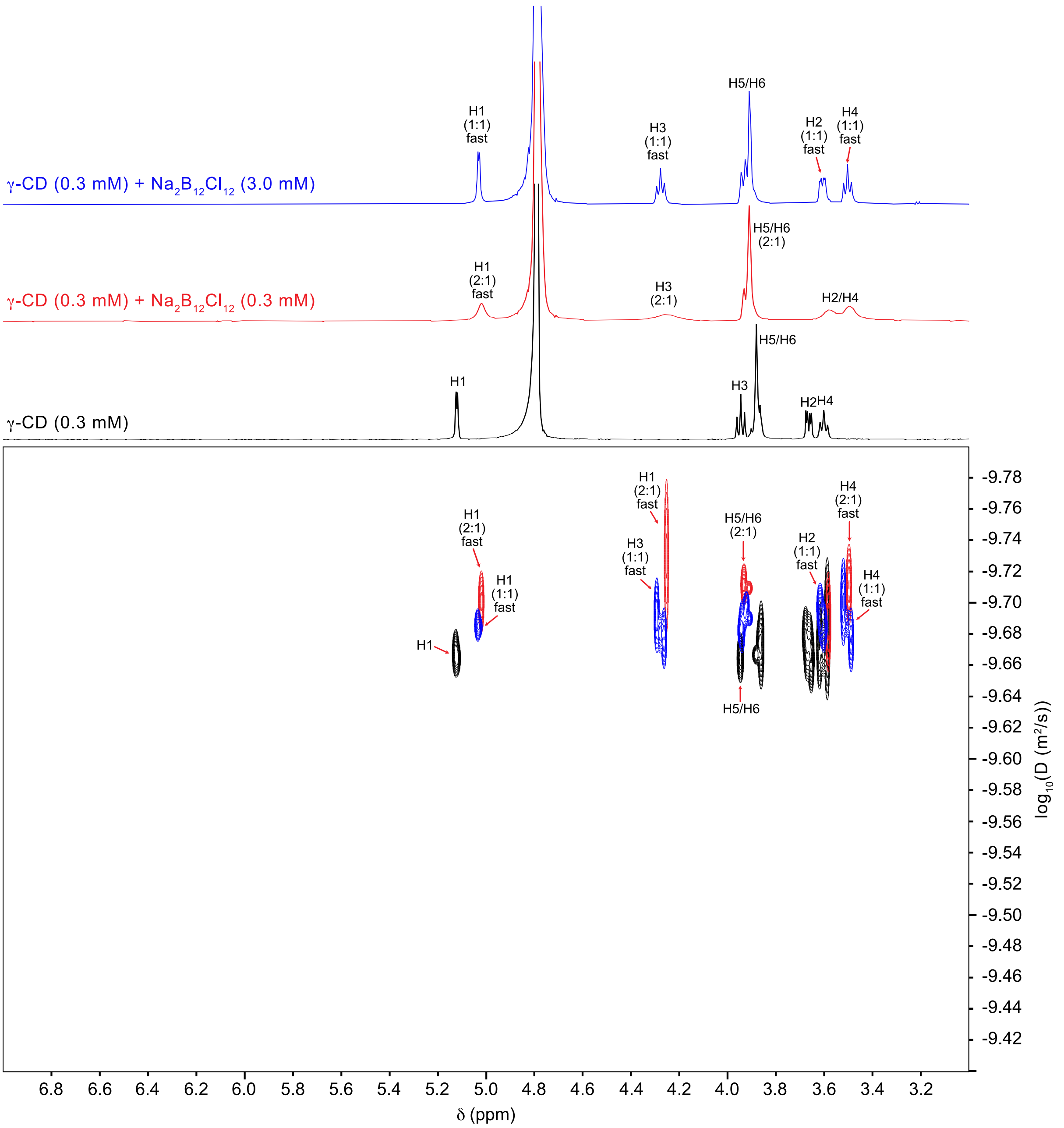

Figure S41. Stacked DOSY spectrum (600 MHz) of γ-CD (0.3 mM) with different equivalents of $Na_2B_{12}Cl_{12}$ in D_2O. *Black*: γ-CD (0.3 mM). *Red*: γ-CD (0.3 mM) and $Na_2B_{12}Cl_{12}$ (0.3 mM). *Blue*: γ-CD (0.3 mM) and $Na_2B_{12}Cl_{12}$ (3.0 mM).

DOSY with γ-CD and $Na_2B_{12}Br_{12}$

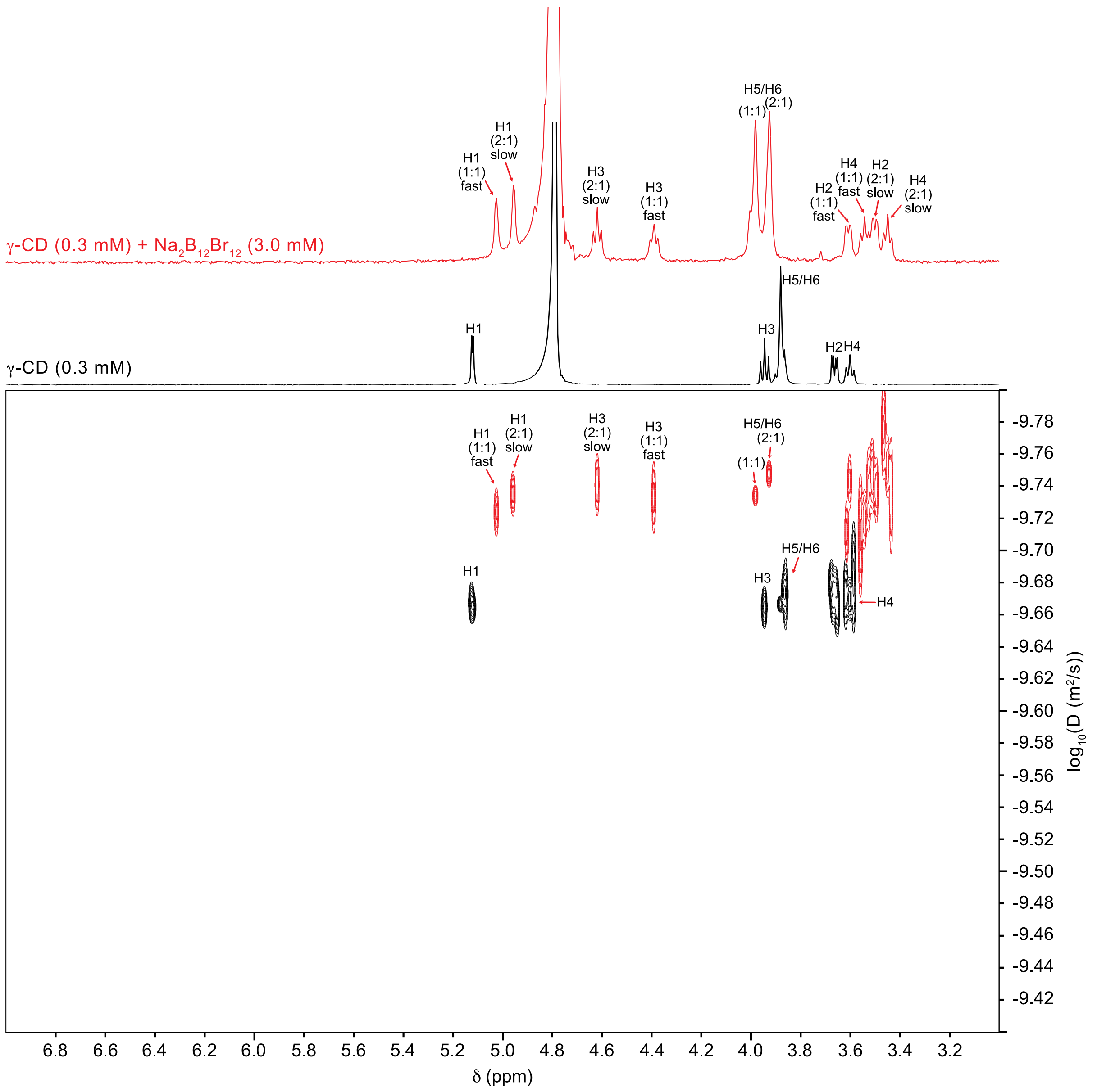

Figure S42. Stacked DOSY spectrum (600 MHz) of γ-CD (0.3 mM) with and without $Na_2B_{12}Br_{12}$ in D_2O. *Black*: γ-CD (0.3 mM). *Red*: γ-CD (0.3 mM) and $Na_2B_{12}Br_{12}$ (3.0 mM).

S11 Algorithm for determining association constants from data obtained in NMR titrations with 2:1 binding in mixed fast and slow exchange

In the titration of γ-CD with $Na_2B_{12}Br_{12}$, we observed formation of a 1:1 complex in fast exchange concurrently with a 2:1 complex in slow exchange (section Section S9, Figure S25). In the following is derived the equations for the determination of the association constants for this binding event. As this is a generally applicable formula, γ-CD will simply be referred to as the host (H) and $Na_2B_{12}Br_{12}$ as the guest (G) in the following derivation. For simplicity in naming the constants in the ensuing algorithm, the association constant for the 1:1 binding event (termed "K_a (1:1)" in the main manuscript) is simply named K_{a1}, while the association constant for the 2:1 binding event (termed "K_a (2:1)" in the main manuscript) is named K_{a2}.

For a situation where the host (H) can bind twice to the guest (G) to give HG and H_2G, we have two equilibria and two association constants, K_{a1} and K_{a2}.

$$\mathrm{H + G \rightleftharpoons HG}$$

$$\mathrm{H + HG \rightleftharpoons H_2G}$$

The association constants are expressed in equations (1) and (2).

$$K_{a1} = \frac{[\mathrm{HG}]}{[\mathrm{H}][\mathrm{G}]} \quad (1)$$

$$K_{a2} = \frac{[\mathrm{H_2G}]}{[\mathrm{HG}][\mathrm{H}]} \quad (2)$$

Equations (1) and (2) can be rearranged to give equations (3) and (4), which define the concentrations of the different complexes.

$$[\mathrm{HG}] = K_{a1}[\mathrm{H}][\mathrm{G}] \quad (3)$$

$$[\mathrm{H_2G}] = K_{a1}K_{a2}[\mathrm{H}]^2[G] \quad (4)$$

The total concentrations of H and G are defined as $[H]_t$ and $[G]_t$, respectively, and give the mass balance equations (5) and (6).

$$[\mathrm{H}]_t = [\mathrm{H}] + [\mathrm{HG}] + 2[\mathrm{H_2G}] \quad (5)$$

$$[\mathrm{G}]_t = [\mathrm{G}] + [\mathrm{HG}] + [\mathrm{H_2G}] \quad (6)$$

Substitution of equations (3) and (4) into equation (6) gives equation (7).

$$[\mathrm{G}]_t = [\mathrm{G}] + K_{a1}[\mathrm{H}][\mathrm{G}] + K_{a1}K_{a2}[\mathrm{H}]^2[\mathrm{G}] \quad (7)$$

Solving equation (7) for [G] gives equation (8). By substituting equation (8) into equations (3) and (4), we obtain equations for [H], [HG] and $[H_2G]$ based on only one unknown concentration ([H]) (equations (8), (9), (10)).

$$[\mathrm{G}] = \frac{[\mathrm{G}]_t}{1+K_{a1}[\mathrm{H}]+K_{a1}K_{a2}[\mathrm{H}]^2} \quad (8)$$

$$[\mathrm{HG}] = \frac{K_{a1}[\mathrm{H}][\mathrm{G}]_t}{1+K_{a1}[\mathrm{H}]+K_{a1}K_{a2}[\mathrm{H}]^2} \quad (9)$$

$$[\mathrm{H_2G}] = \frac{K_{a1}K_{a2}[\mathrm{H}]^2[\mathrm{G}]_t}{1+K_{a1}[\mathrm{H}]+K_{a1}K_{a2}[\mathrm{H}]^2} \quad (10)$$

Substituting equations (8), (9) and (10) into mass balance equation (5) gives equation (11).

$$[\mathrm{H}]_\mathrm{t} = [\mathrm{H}] + \frac{K_\mathrm{a1}[\mathrm{H}][\mathrm{G}]_\mathrm{t} + 2K_\mathrm{a1}K_\mathrm{a2}[\mathrm{H}]^2[\mathrm{G}]_\mathrm{t}}{1 + K_\mathrm{a1}[\mathrm{H}] + K_\mathrm{a1}K_\mathrm{a2}[\mathrm{H}]^2} \qquad (11)$$

Rearranging equation (11) results in the following cubic equation for [H]:

$$0 = K_\mathrm{a1}K_\mathrm{a2}[\mathrm{H}]^3 + K_\mathrm{a1}[\mathrm{H}]^2 + 2K_\mathrm{a1}K_\mathrm{a2}[\mathrm{G}]_\mathrm{t}[\mathrm{H}]^2 - K_\mathrm{a1}K_\mathrm{a2}[\mathrm{H}]_\mathrm{t}[\mathrm{H}]^2 + K_\mathrm{a1}[\mathrm{G}]_\mathrm{t}[\mathrm{H}] - K_\mathrm{a1}[\mathrm{H}]_\mathrm{t}[\mathrm{H}] + [\mathrm{H}] - [\mathrm{H}]_\mathrm{t}$$

For convenience, we can express the cubic equation as equation (12).

$$\mathrm{A}[\mathrm{H}]^3 + \mathrm{B}[\mathrm{H}]^2 + \mathrm{C}[\mathrm{H}] + \mathrm{D} = 0 \qquad (12)$$

wherein

$$\mathrm{A} = K_\mathrm{a1}K_\mathrm{a2}$$

$$\mathrm{B} = K_\mathrm{a1} + 2K_\mathrm{a1}K_\mathrm{a2}[\mathrm{G}]_\mathrm{t} - K_\mathrm{a1}K_\mathrm{a2}[\mathrm{H}]_\mathrm{t}$$

$$\mathrm{C} = 1 + K_\mathrm{a1}[\mathrm{G}]_\mathrm{t} - K_\mathrm{a1}[\mathrm{H}]_\mathrm{t}$$

$$\mathrm{D} = -[\mathrm{H}]_\mathrm{t}$$

With the [H]-dependent equation in hand, we must now derive equations that relate the NMR observable parameters to K_{a1}, K_{a2}, [H] and known concentrations $[H]_t$ and $[G]_t$.

In the situation where the 1:1 binding is observed to be in fast exchange, and the 2:1 binding is observed to be in slow exchange, we can directly determine the concentrations $[H_2G]$ and ([H]+[HG]) by integration of the two sets of peaks (as for example the H1 peaks in panel A of Figure S25).

In the NMR spectra obtained during the titration, we can detect the formation of HG from H by the change in chemical shift of the peak corresponding to these two species in fast exchange. The observed chemical shift (δ_{obs}) is determined by the weighted average of the chemicals shifts of H and HG (δ_H, δ_{HG}) according to equation (13).

$$\delta_\mathrm{obs} = \delta_\mathrm{H}\frac{[\mathrm{H}]}{[\mathrm{H}]+[\mathrm{HG}]} + \delta_\mathrm{HG}\frac{[\mathrm{HG}]}{[\mathrm{H}]+[\mathrm{HG}]} \qquad (13)$$

Rearranging equation (13) gives equation (14) where $\Delta\delta_{HG}$ is the chemical shift difference between δ_{HG} and δ_H, and $\Delta\delta_{obs}$ is the observed chemical shift change during the titration.

$$\delta_\mathrm{obs} = \left(1 - \frac{[\mathrm{HG}]}{[\mathrm{H}]+[\mathrm{HG}]}\right)\delta_\mathrm{H} + \frac{[\mathrm{HG}]}{[\mathrm{H}]+[\mathrm{HG}]}\delta_\mathrm{HG}$$

$$\delta_\mathrm{obs} - \delta_\mathrm{H} = (\delta_\mathrm{HG} - \delta_\mathrm{H})\frac{[\mathrm{HG}]}{[\mathrm{H}]+[\mathrm{HG}]}$$

$$\Delta\delta_\mathrm{obs} = \frac{[\mathrm{HG}]}{[\mathrm{H}]+[\mathrm{HG}]}\Delta\delta_\mathrm{HG} \qquad (14)$$

Rearranging equation (14) gives equation (15).

$$([\mathrm{H}] + [\mathrm{HG}])\Delta\delta_\mathrm{obs} = [\mathrm{HG}]\Delta\delta_\mathrm{HG} \qquad (15)$$

Substitution of equation (9) into equation (15) and dividing both sides by $[H]_t$ gives equation (16).

$$\left(\frac{[\mathrm{H}]+[\mathrm{HG}]}{[\mathrm{H}]_\mathrm{t}}\right)\Delta\delta_\mathrm{obs} = \frac{K_\mathrm{a1}[\mathrm{H}][\mathrm{G}]_\mathrm{t}}{[\mathrm{H}]_\mathrm{t} + K_\mathrm{a1}[\mathrm{H}]_\mathrm{t}[\mathrm{H}] + K_\mathrm{a1}K_\mathrm{a2}[\mathrm{H}]_\mathrm{t}[\mathrm{H}]^2}\Delta\delta_\mathrm{HG} \qquad (16)$$

From equation (10) we have an expression for $[H_2G]$, and by multiplying both sides by $2/[H]_t$ we obtain equation (17).

$$\frac{2[H_2G]}{[H]_t} = \frac{2K_{a1}K_{a2}[H]^2[G]_t}{[H]_t + K_{a1}[H]_t[H] + K_{a1}K_{a2}[H]_t[H]^2} \quad (17)$$

Together, equations (16) and (17) describe the relationships between the observed chemical shift change, the sum of the molar fractions of the free host and 1:1 complex $([H]+[HG])/[H]_t$, and the concentration of the 2:1 complex ($[H_2G]$) and the concentration of added guest ($[G]_t$).

Experimentally generated isotherms are made by plotting $(([H] + [HG])/[H]_t)\Delta\delta_{obs}$ and $2[H_2G]/[H]_t$ against $[G]_t$. Values for ([H]+[HG]) and $2[H_2G]$ are obtained from the integrals of signals for the species in slow exchange. The two isotherms are simultaneously fitted to equations (16) and (17), as described below.

Although the value of [H] in equations (16) and (17) varies depending on $[H]_t$ and $[G]_t$ (as described by equation (12)), the solution can be readily approached through an iterative data fitting procedure. We have adapted the method described by Hargrove *et al.*[10] for fitting of 1:2 binding in optical binding isotherms using the non-linear curve fitting program OriginPro 2019. The script we wrote for the fitting function in Origin is shown below. The code is written in LabTalk Script. Briefly, equation (12) is solved numerically for [H] using Newton's method while fitting the experimental data to equations (16) and (17). Given a set of estimated initial values for K_{a1}, K_{a2} and $\Delta\delta_{HG}$, the program iteratively determines the parameter values that best fit the experimental data in terms of both the integrals and the chemical shift changes.

```
A=K1*K2;
B=K1+2*K1*K2*x-K1*K2*Ht;
C=1+K1*x-K1*Ht;
D=-Ht;
for (H=Ht, step=1; abs(step)>1e-15; H=H-step){
        step=(A*H*H*H+B*H*H+C*H+D)/(3*A*H*H+2*B*H+C);};
y1=(Dd*K1*H*x)/(Ht+K1*Ht*H+K1*K2*Ht*H*H);
y2=(2*K1*K2*H*H*x)/(Ht+K1*Ht*H+K1*K2*Ht*H*H)
```

Where K1, K2, Dd, Ht, x, y1, and y2 represent K_{a1}, K_{a2}, $\Delta\delta_{HG}$, $[H]_t$, $[G]_t$, $(([H] + [HG])/[H]_t)\Delta\delta_{obs}$ and $2[H_2G]/[H]_t$, respectively.

S12 ITC titrations of δ-CD or ε-CD with $Na_2B_{12}X_{12}$ (X = Cl, Br, I)

In all ITC titrations, the CD (δ-CD or ε-CD, typically in 5.0 µL injection volume aliquots) was titrated into a solution of the sodium salt of the dodecaborate ion being investigated (typically at a specific concentration in 40 – 60 µM range). A blank run (injection of CD solution into water) was subtracted from each titration to account for the heat of dilution as well as heat evolved as result of turbulent mixing. These heats were found to be low, as seen in the two figures below, that show a blank run for δ-CD and ε-CD, respectively.

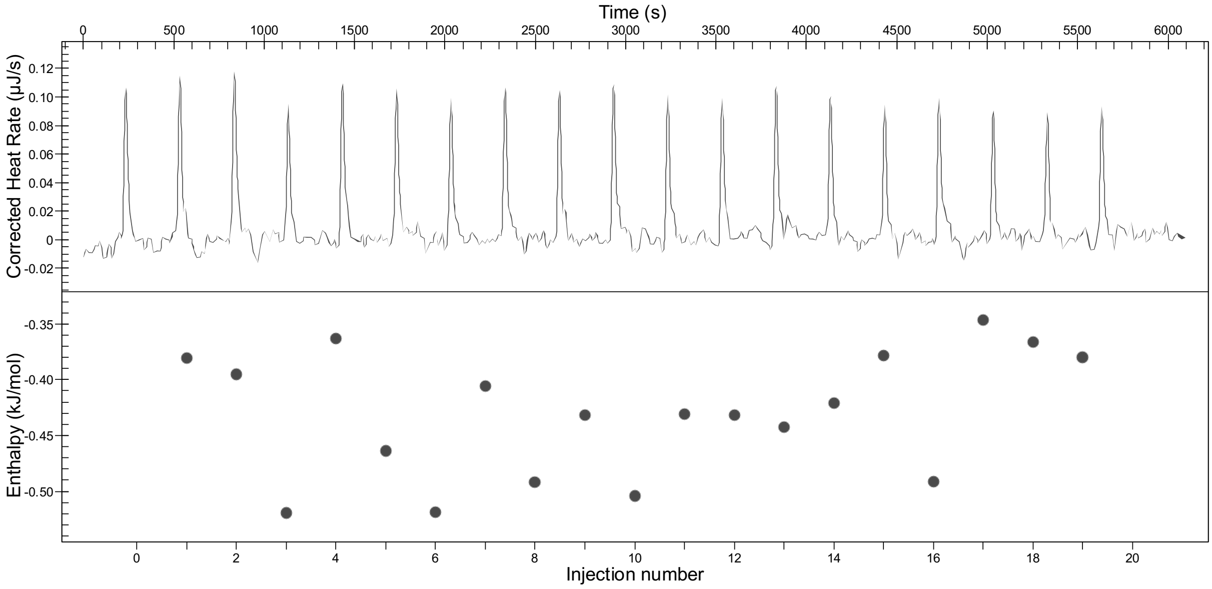

Figure S43. ITC blank run of a solution of δ-CD (1.2 mM in water) into water. Observed baseline-corrected heat rate during the injection of 19 aliquots of 5.0 µL (top), and the resulting enthalpies of each injection (bottom).

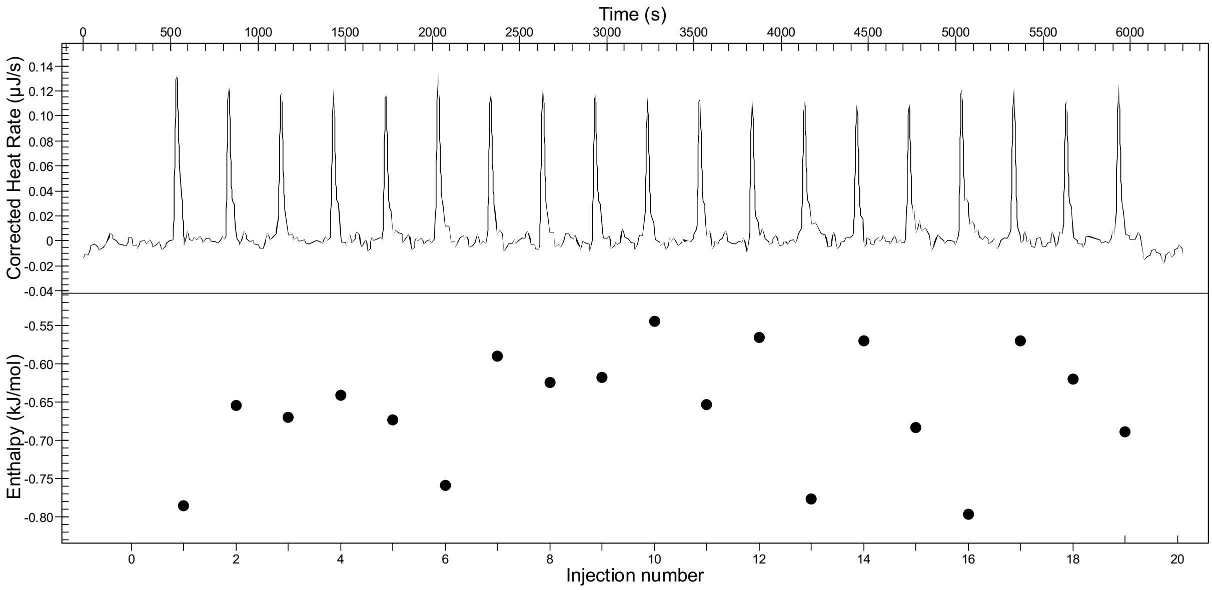

Figure S44. ITC blank run of a solution of ε-CD (1.0 mM in water) into water. Observed baseline-corrected heat rate during the injection of 19 aliquots of 5.0 µL (top), and the resulting enthalpies of each injection (bottom).

The enthalpies resulting from titrations (after subtraction of the blank runs) were fitted to a 1:1 binding model, since the NMR titrations in all cases indicated a 1:1 binding mode (section S9). All ITC titration data obtained gave good fits with stoichiometry values (n) close to 1.

S12.1 δ-CD and $Na_2B_{12}Cl_{12}$

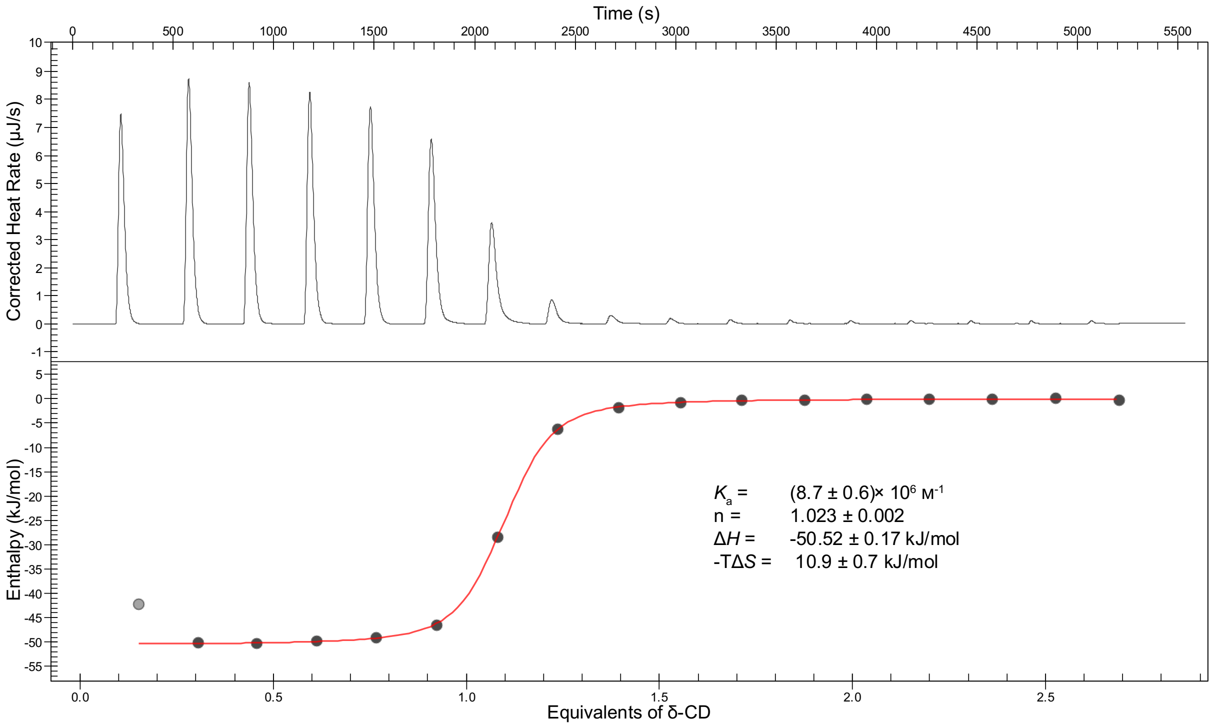

Figure S45. ITC titration of a solution of δ-CD (1.2 mM in water) into $Na_2B_{12}Cl_{12}$ (40 µM in water). Observed baseline-corrected heat rate during the injection of 17 aliquots of 5.0 µL (top), and the resulting enthalpies of each injection (black circles) after subtraction of a blank run (titration of δ-CD (1.2 mM) into water) along with best fit (red line) to a 1:1 binding model. Reported errors on fit are at the 95 % confidence interval based on 5000 statistical trials. The enthalpy value obtained for the first injection (gray circle) is off due to diffusion of titrant into the cell during the instrument equilibration. This data point was rejected and not included in the fit.

S12.2 δ-CD and $Na_2B_{12}Br_{12}$

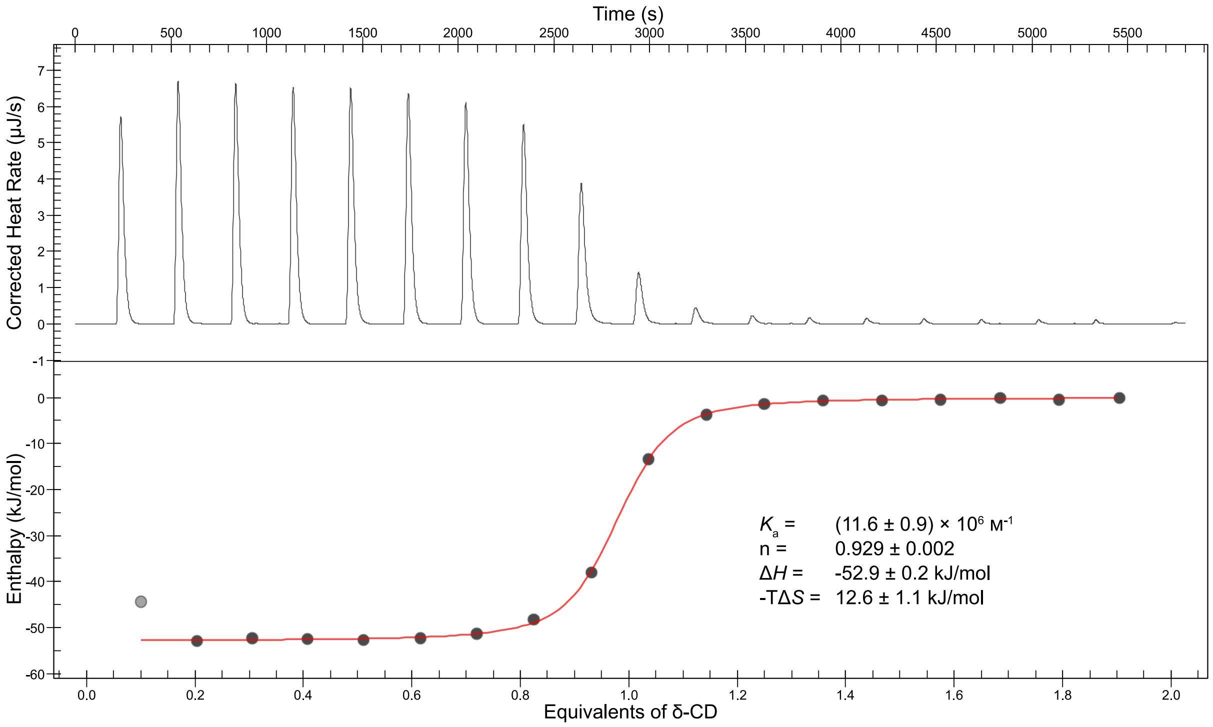

Figure S46. ITC titration of a solution of δ-CD (0.8 mM in water) into $Na_2B_{12}Br_{12}$ (40 µM in water). Observed baseline-corrected heat rate during the injection of 18 aliquots of 5.0 µL (top), and the resulting enthalpies of each injection (black circles) after subtraction of a blank run (titration of δ-CD (1.2 mM) into water) along with best fit (red line) to a 1:1 binding model. Reported errors on fit are at the 95 % confidence interval based on 5000 statistical trials. The enthalpy value obtained for the first injection (gray circle) is off due to diffusion of titrant into the cell during the instrument equilibration. This data point was rejected and not included in the fit.

S12.3 δ-CD and $Na_2B_{12}I_{12}$

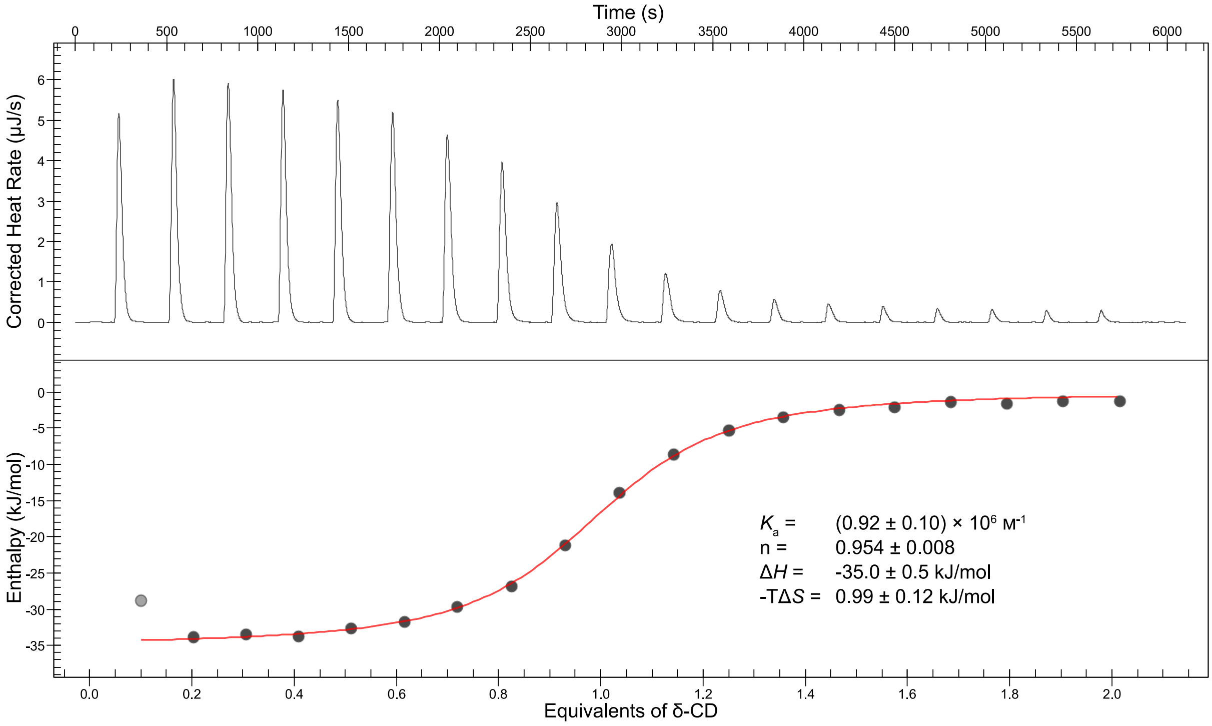

Figure S47. ITC titration of a solution of δ-CD (1.2 mM in water) into $Na_2B_{12}I_{12}$ (60 µM in water). Observed baseline-corrected heat rate during the injection of 19 aliquots of 5.0 µL (top), and the resulting enthalpies of each injection (black circles) after subtraction of a blank run (titration of δ-CD (1.2 mM) into water) along with best fit (red line) to a 1:1 binding model. Reported errors on fit are at the 95 % confidence interval based on 5000 statistical trials. The enthalpy value obtained for the first injection (gray circle) is off due to diffusion of titrant into the cell during the instrument equilibration. This data point was rejected and not included in the fit.

S12.4 ε-CD and $Na_2B_{12}I_{12}$

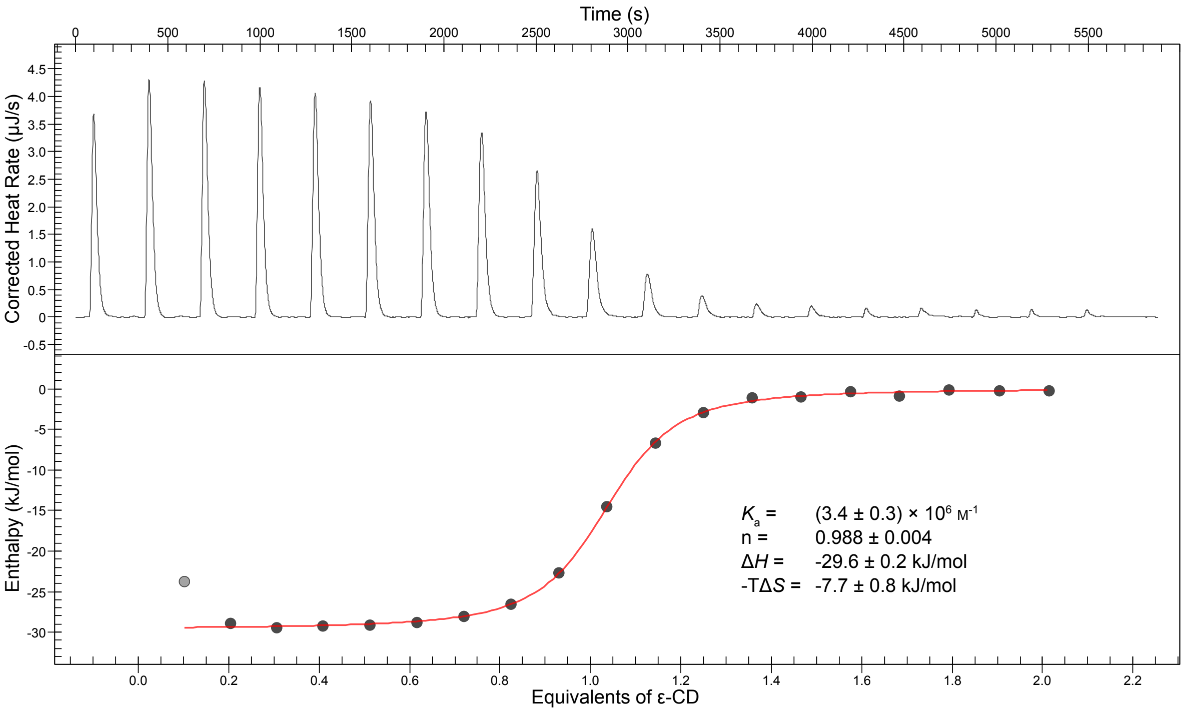

Figure S48. ITC titration of a solution of ε-CD (1.0 mM in water) into $Na_2B_{12}I_{12}$ (50 μM in water). Observed baseline-corrected heat rate during the injection of 19 aliquots of 5.0 μL (top), and the resulting enthalpies of each injection (black circles) after subtraction of a blank run (titration of ε-CD (1.0 mM) into water) along with best fit (red line) to a 1:1 binding model. Reported errors on fit are at the 95 % confidence interval based on 5000 statistical trials. The enthalpy value obtained for the first injection (gray circle) is off due to diffusion of titrant into the cell during the instrument equilibration. This data point was rejected and not included in the fit.

S13 Simulation of library distributions

A depiction of the equilibria being modelled by the *DCLsim* software is shown in Fig. 3 of the main manuscript. Three models were made, one for each dodecaborate (i.e. X = Cl, Br, or I, respectively), and graphical representations of the models with the binding constants included is shown in Figure S49 below. To simulate a dynamic combinatorial library, we first determined relative formation constants for α-, β-, γ-, δ-, ε-, and ζ-CD, as described in Section S13.1.

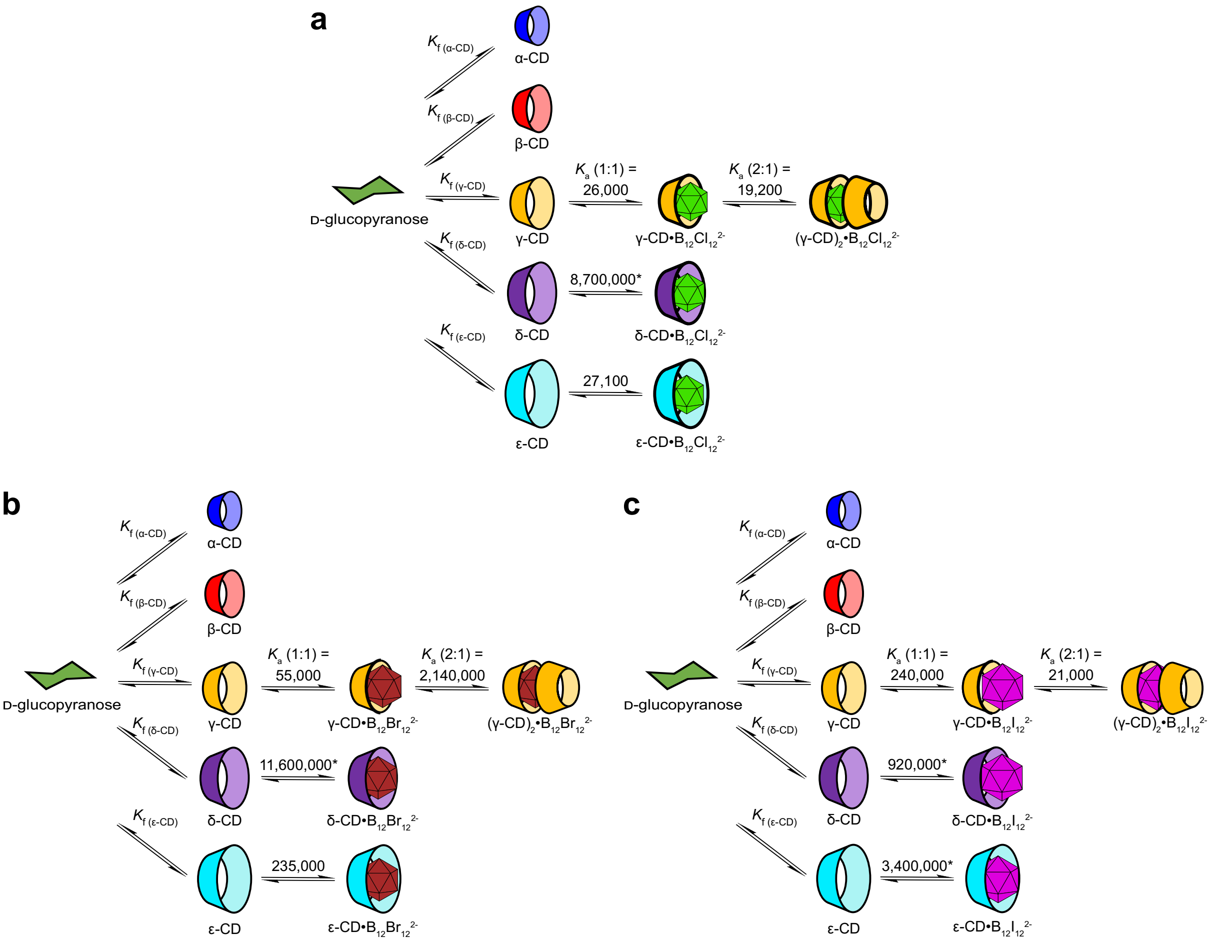

Figure S49. *DCLsim* models used to simulate library distributions. All K_a values were determined at 25 °C by ^{1}H NMR spectroscopy titration in D_2O (Section S9), except values marked by an asterisk (*), which were determined by ITC in water (section S12). (a) *DCLsim* model of libraries templated with $B_{12}Cl_{12}^{2-}$. (b) *DCLsim* model of libraries templated with $B_{12}Br_{12}^{2-}$. (b) *DCLsim* model of libraries templated with $B_{12}I_{12}^{2-}$.

S13.1 Determination of relative formation constants (rK_f) of α-, β-, γ-, δ-, ε-, and ζ-CD

To determine relative formation constants of α-, β-, γ-, δ-, ε- and ζ-CD, a CGTase-mediated untemplated DCL of CDs was set up and monitored using ^{1}H NMR spectroscopy. DMF (1.00 mM) was used as an internal standard for calibration of integrals of CDs to determine the total CD concentration.

A stock solution of α-CD (250 µl, 20 mg/ml) in D_2O sodium phosphate buffer (50 mM, pH 7.5) was mixed with D_2O sodium phosphate buffer (238.5 µl, 50 mM, pH 7.5), spin-filtered CGTase stock solution (6.5 µl) and a stock solution of DMF (5 µl, 100 mM in D_2O sodium phosphate buffer) to give a total reaction volume of 500 µl and final concentrations of α-CD (10 mg/ml), CGTase (13 µl stock solution per ml reaction mixture) and DMF (1.00 mM). The reaction was then monitored using ^{1}H NMR (800 MHz, 25 ºC) spectroscopy (Figure S50A) and the integrals of α-, β-, γ-, δ-, ε- and ζ-CD and DMF were used to calculate the concentrations of each CD and a total CD concentration over time (Figure S50B). The shimming becomes poor as the experiment progresses, so the sample was shimmed again before the final spectrum was acquired. Note that the quantity of enzyme used is lower than in the DCLs discussed in section S8, such that the DCL evolved more slowly and could be carefully monitored.

Individual CD peaks could be assigned based on previous ^{1}H–^{13}C heteronuclear single quantum coherence (HSQC) NMR experiments.[11] Due to significant overlap between peaks of interest, global spectral deconvolution (GSD) of the final spectrum was carried out in the software Mestrenova 11.0 (Figure S51). The relative formation constant for each CD was then calculated according to equation (18) based on the integrals of the deconvoluted peaks.

$$rK_f(CDn) = [CDn] / [G_0]^n \qquad (18)$$

where CDn is a CD with a degree of polymerization n, rK_f(CDn) is the relative formation constant of CDn, [CDn] is the concentration (M) of CDn and [G_0] is the total concentration of glucose units (M) in all CDs (ie. 6 × [α-CD] + 7 × [β-CD] + 8 × [γ-CD] + 9 × [δ-CD] + 10 × [ε-CD] + 11 × [ζ-CD]). Note that the rK_f value can only be calculated after equilibrium has been reached within the CD subsystem, during which time, some linear α-1,4-glucans are formed, and the total concentration of CDs has decreased. $[G]_0$ was measured at this time point (21.7 h). The calculated rK_f values are 1.44×10^5 M^{-5}, 4.27×10^6 M^{-6}, 1.66×10^7 M^{-7}, 2.15×10^7 M^{-8}, 1.99×10^8 M^{-9}, and 2.44×10^9 M^{-10}, for α-, β-, γ-, δ-, ε- and ζ-CD, respectively.

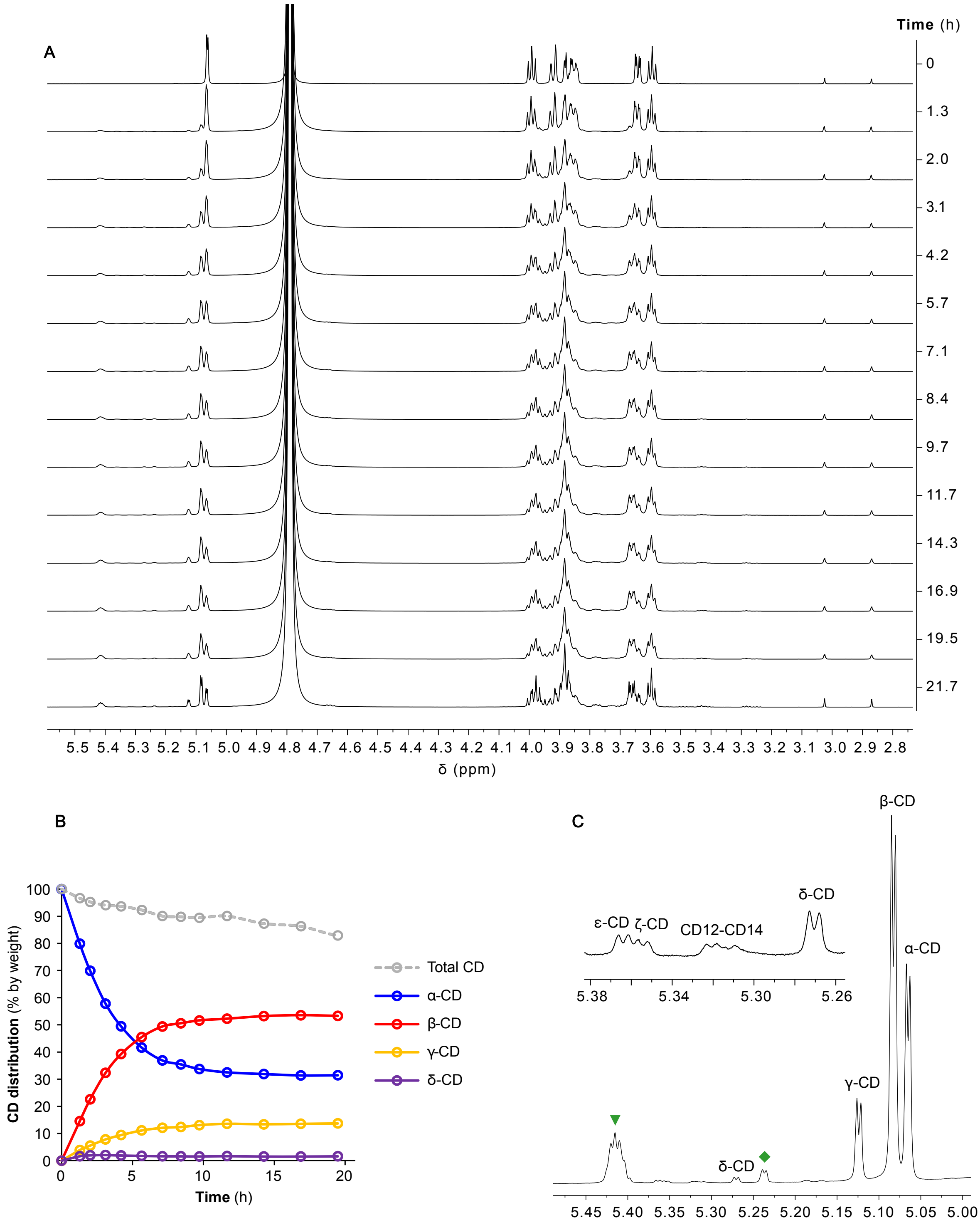

Figure S50. CGTase-mediated untemplated DCL of CDs used to determine r*K*f. (A) ^{1}H NMR spectra (800 MHz) of the reaction at various time points. (B) Plot of the CD distribution and % of all glucans in the library that are CDs over time, based on integrals from the ^{1}H NMR spectra. (C) Partial ^{1}H NMR spectrum of the reaction at the final time point (21.7 h) with assignment of the anomeric proton signals (H1) of CDs and α-1,4-glucans present in the reaction mixture, based on previous studies in our laboratory.[11] Green triangle: Internal and non-reducing end of linear α-1,4-glucans. Green diamond: Reducing end (α-anomer) of linear α-1,4-glucans.

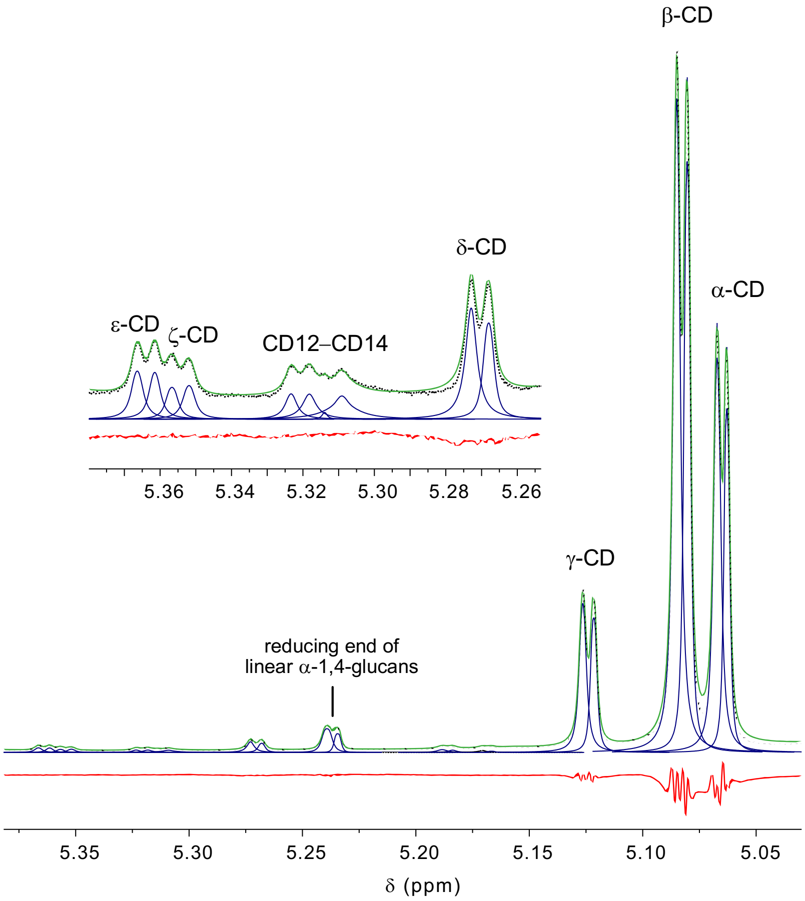

Figure S51. Peak deconvolution of the ^{1}H NMR spectrum (partial spectrum shown) of the final data point (21.7 h) used to obtain integrals of CDs to calculate rK_f. Black dotted line: Original ^{1}H NMR spectrum. Green line: Fitted spectrum (cumulative fitted peak curves). Blue lines: Fitted peak curves. Red line: Residuals.

S13.2 Simulation of the untemplated DCL at 6 h

To simulate an untemplated DCL, the inputs required are the relative formation constants for each CD (denoted rK_f), information about how many *DCLsim* monomers (in this case the imaginary D-glucopyranose monomer) are needed to make each library member (i.e. 6 for α-CD, 7 for β-CD, 8 for γ-CD and so on) and the total concentration of the *DCLsim* monomer (i.e. $[G]_0$) at the timepoint for which the simulation is made. For the untemplated DCL, equilibrium was reached after 6 h at which time the total concentration of glucose monomers making up the CD sub-library was 3.23 mg/mL (19.92 mM). The resulting simulated CD distribution for the untemplated library perfectly matched the observed distribution (Fig. 3b in the. manuscript).

S13.3 Simulation of templated DCLs at 48 h with 5 mM template

The next step of the *DCLsim* model is then to add the association constants for the formation of complexes with the template (T), which is done in practice by defining a new species for each CD/dodecaborate complex, whose relative formation constant is then the product of all the relative formation and/or association constants in the path to its formation from the imaginary *DCLsim* monomer. For example, the relative formation constant used in the model for the γ-CD•$B_{12}Cl_{12}^{2-}$ complex is r$K_{f(\gamma\text{-CD})}\cdot K_{a(1:1),\gamma\text{-CD}\bullet B_{12}Cl_{12}^{2-}}$, and to make this complex, the model needs use 8 *DCLsim* monomers and one template. Meanwhile, the overall relative formation constant used in the model for the (γ-CD)$_2$•$B_{12}Cl_{12}^{2-}$ complex is r$K_{f(\gamma\text{-CD})}\cdot$r$K_{f(\gamma\text{-CD})}\cdot K_{a(1:1),\gamma\text{-CD}\bullet B_{12}Cl_{12}^{2-}}\cdot K_{a(2:1),(\gamma\text{-CD})_2\bullet B_{12}Cl_{12}^{2-}}$ and to make this complex, the model needs to use 16 *DCLsim* monomers and one template.

Relative formation constants for all library members (free CDs, as well as CD·T and $(CD)_2$·T complexes) used as inputs in *DCLsim* are listed in Table S1.

Table S1: Overall relative formation constants used as input values in *DCLsim*[a]

Library member	rK_f	rK_f·$K_{a(1:1)}$	rK_f· rK_f·$K_{a(1:1)}$·$K_{a(2:1)}$
**α-CD**	1.443×10^5 M^{-5}		
**β-CD**	4.265×10^6 M^{-6}		
**γ-CD**	1.661×10^7 M^{-7}		
**δ-CD**	2.152×10^7 M^{-8}		
**ε-CD**	1.992×10^8 M^{-9}		
**γ-CD•$B_{12}Cl_{12}^{2-}$**		4.318×10^{11} M^{-8}	
**(γ-CD)$_2$•$B_{12}Cl_{12}^{2-}$**			1.377×10^{23} M^{-16}
**δ-CD•$B_{12}Cl_{12}^{2-}$**		1.873×10^{14} M^{-9}	
**ε-CD•$B_{12}Cl_{12}^{2-}$**		5.398×10^{12} M^{-10}	
**γ-CD•$B_{12}Br_{12}^{2-}$**		9.135×10^{11} M^{-8}	
**(γ-CD)$_2$•$B_{12}Br_{12}^{2-}$**			3.247×10^{25} M^{-16}
**δ-CD•$B_{12}Br_{12}^{2-}$**		2.497×10^{14} M^{-9}	
**ε-CD•$B_{12}Br_{12}^{2-}$**		4.681×10^{13} M^{-10}	
**γ-CD•$B_{12}I_{12}^{2-}$**		3.986×10^{12} M^{-8}	
**(γ-CD)$_2$•$B_{12}I_{12}^{2-}$**			1.390×10^{24} M^{-16}
**δ-CD•$B_{12}I_{12}^{2-}$**		1.980×10^{13} M^{-9}	
**ε-CD•$B_{12}I_{12}^{2-}$**		6.773×10^{14} M^{-10}	

[a] The values are reported with four significant digits since that is the level of precision that was put into the DCLsim input file. The errors associated with these values are likely much larger than the number of significant digits suggest.

Simulations were made for the three DCLs templated with 5 mM of $Na_2B_{12}Cl_{12}$, $Na_2B_{12}Br_{12}$ $Na_2B_{12}I_{12}$. Since we consider only the CDs in the *DCLsim* simulations, the total concentration of the building block $[G]_0$ that is put into the model must be equal to the total concentration of D-glucopyranose units that remain in the CD sub-system at the time of observation. Therefore, the simulated values for the 48-hour timepoint that are shown in Fig. 3b of the main manuscript were simulated with $[G]_0$ of 3.7330 mg/mL (23.024 mM) for $Na_2B_{12}Cl_{12}$, 6.2752 mg/mL (38.704 mM) for $Na_2B_{12}Br_{12}$ and 4.4026 mg/mL (27.154 mM) for $Na_2B_{12}I_{12}$. Figure S52 shows a screen shot of the input data for the simulation of the 48 h timepoint in the DCL templated with $Na_2B_{12}Cl_{12}$.

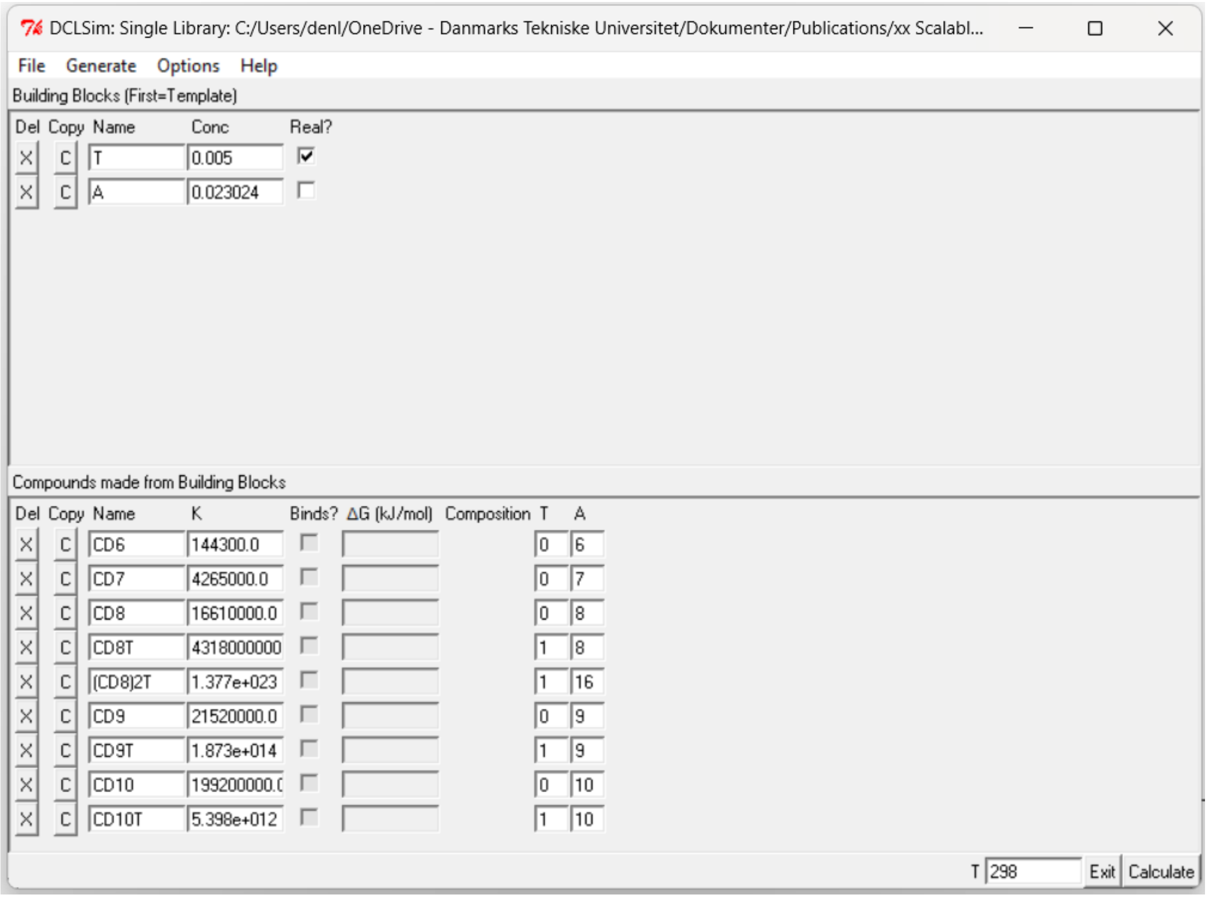

Figure S52.. Screen shot of the input data for the simulations of the 48 h timepoint in the DCL templated with 5 mM of $Na_2B_{12}Cl_{12}$. T denotes the template and A denotes the imaginary D-glucopyranose monomer.

The output file from the *DCLsim* calculation contains the molar concentrations of all library members. To turn those values into CD distributions in percentage by weight, the concentrations for all library members that contain a specific CD are combined (taking the number of equivalents of the CD in the specific complex into account), multiplied by the molecular weight of each CD and divided by the total concentration by weight of CDs ($[G]_0$).

S13.4 Simulation of DCLs at different time points.

The three DCLs templated with 5 mM of $Na_2B_{12}Cl_{12}$, $Na_2B_{12}Br_{12}$ and $Na_2B_{12}I_{12}$ were simulated at 24 h, 48 h and 72 h timepoints, and the distribution of CDs observed and predicted for these time points are shown in Figure S53. The 48-hour timepoint was chosen for comparison between template effects in the main manuscript (Figure 1) as this is the earliest timepoint where all three libraries were deemed to be at equilibrium. For both $Na_2B_{12}Br_{12}$ and $Na_2B_{12}I_{12}$, the simulations and the experiments matched well already after 24 hours (indicating that equilibrium had been reached). For the $Na_2B_{12}Cl_{12}$-templated library, however, the DCL took longer to evolve and only after 48 hours does the ratio γ-CD to δ-CD match the simulation.

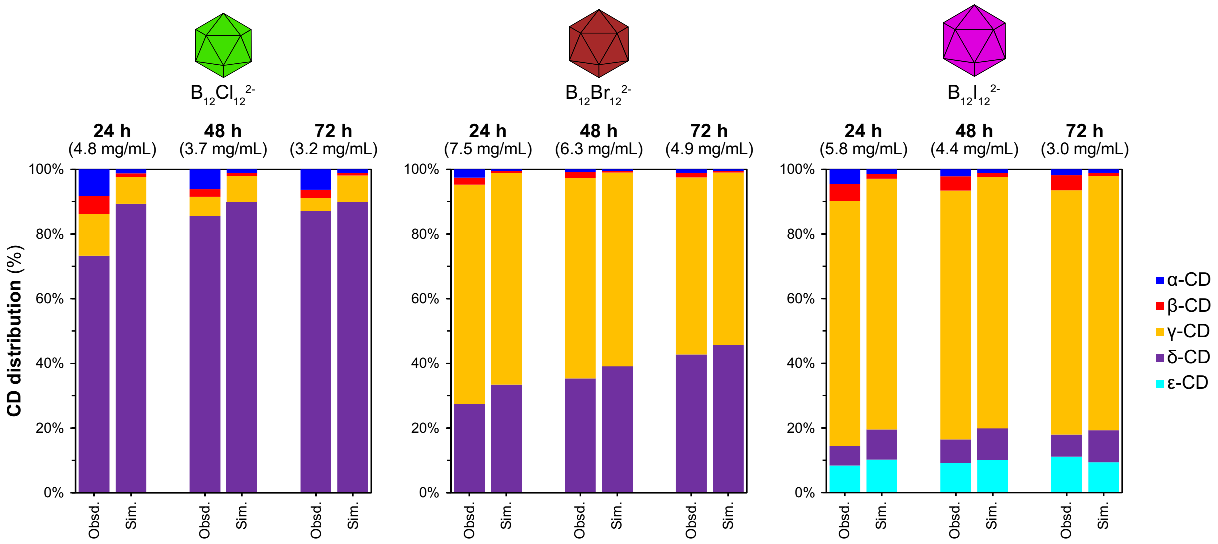

Figure S53. Observed (obsd.) CD distributions compared to simulated (sim.) CD distributions at the 24, 48 and 72-hour timepoints in libraries templated with 5 mM of $Na_2B_{12}Cl_{12}$, $Na_2B_{12}Br_{12}$ and $Na_2B_{12}I_{12}$. The observed total CD concentration (noted below each timepoint) was input as the total building block concentration ($[G]_0$) for the simulation in each case.

S13.5 Simulation of DCLs with varying template concentration

The *DCLsim* models were used to simulate the CD distributions over a range of dodecaborate concentrations. Simulations were performed all the way up to 500 mM, even though the dodecaborates might not be soluble at such high concentrations. For these simulations, the concentration of building block $[G]_0$ was set at 4 mg/mL (24.67 mM), since this roughly compares to the observed concentration of CDs remaining when equilibrium is reached in a library started from α-CD at 10 mg/mL. Since most of the changes in composition happens in the 0 – 20 mM range, that range is shown in the top graphs in Figure S54 below. Observed values are shown as data points in the top graphs in Figure S54 below. For the closest approximation to the simulated value, the observed values were all taken from the data point where the total CD concentration was closest to 4 mg/mL, which in all cases were the 48-hour time-point, except with 2 mM $Na_2B_{12}Cl_{12}$ (3.96 mg/mL observed at 24 hours) and with 5 mM $Na_2B_{12}Br_{12}$ (4.87 mg/mL observed at 72 hours). In all cases, the observed distributions are close to the simulated distributions, except for the presence of higher concentrations of α-CD and β-CD than simulated, probably due to fast formation of these small CDs from the linear species (that are not included in the simulations).

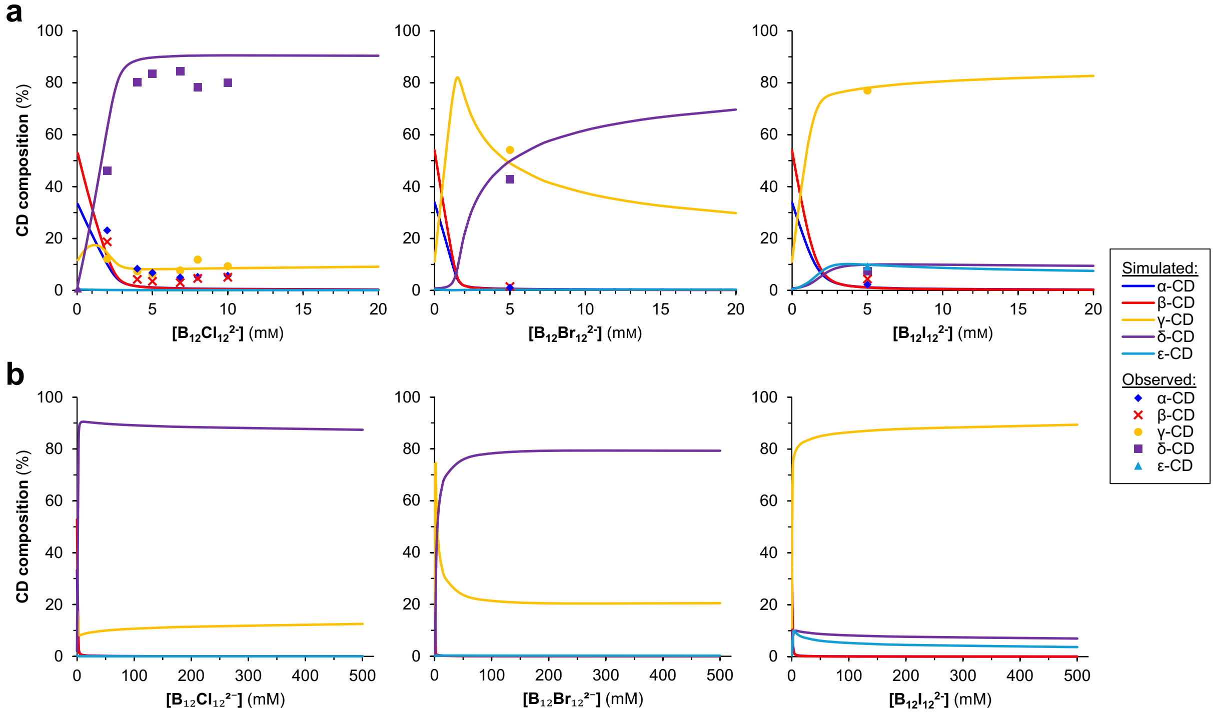

Figure S54. *DCLsim* results showing CD distributions as a function of dodecaborate concentration assuming a total CD concentration of 4 mg/mL. (a) Simulated (lines) and observed (data points) CD distributions in the range from 0 to 20 mM. The observed data points were taken from the actual data point where the observed total CD concentration ($[G]_0$) was closest to 4 mg/mL. (b) Simulated CD distributions in the range from 0 to 500 mM.

S14 References

1. Erichsen, A.; Peters, G. H. J. & Beeren, S. R. Templated Enzymatic Synthesis of δ-Cyclodextrin. *J. Am. Chem. Soc.* **2023**, *145*, 4882.
2. Larsen, D. & Beeren, S. R. Building up cyclodextrins from scratch - templated enzymatic synthesis of cyclodextrins directly from maltose. *Chem. Commun.* **2021**, *57*, 2503.
3. Thordarson, P. Determining association constants from titration experiments in supramolecular chemistry. *Chem. Soc. Rev.* **2011**, *40*, 1305.
4. Brynn Hibbert, D. & Thordarson, P. The death of the Job plot, transparency, open science and online tools, uncertainty estimation methods and other developments in supramolecular chemistry data analysis. *Chem. Commun.* **2016**, *52*, 12792.
5. Geis, V.; Guttsche, K.; Knapp, C.; Scherer, H. & Uzun, R. Synthesis and characterization of synthetically useful salts of the weakly-coordinating dianion [B12Cl12]2-. *Dalton Trans.* **2009**, 2687.
6. Tiritiris, I. & Schleid, T. Die kristallstrukturen der dicaesium-dodekahalogeno-closo-dodekaborate Cs2[B12X12] (X = Cl, Br, I) und ihrer hydrate. *Z Anorg Allg Chem* **2004**, *630*, 1555.
7. Juhasz, M. A.; Matheson, G. R.; Chang, P. S.; Rosenbaum, A. & Juers, D. H. Microwave-Assisted Iodination: Synthesis of Heavily Iodinated 10-Vertex and 12-Vertex Boron Clusters. *Synthesis and Reactivity in Inorganic, Metal-Organic and Nano-Metal Chemistry* **2016**, *46*, 583.
8. Ishizuka, Y. *et al.* Application of ultra-high magnetic field for saccharide molecules: 1H NMR spectra of 6-O-α-d-glucopyranosyl-cyclomaltoheptaose and -cyclomaltohexaose. *Carbohydr. Res.* **2005**, *340*, 1343.
9. MacCarthy, P. Simplified Experimental Route for Obtaining Job's Curves. *Anal. Chem.* **1978**, *50*, 2165.
10. Hargrove, A. E.; Zhong, Z.; Sessler, J. L.; Anslyn, E. V. Algorithms for the Determination of Binding Constants and Enantiomeric Excess in Complex Host : Guest Equilibria Using Optical Measurements. *New J. Chem.* **2010**, *34*, 348.
11. Larsen, D.; Erichsen, A.; Masciotta, G.; Meier, S. & Beeren, S. R. Quantitative determination of the binding capabilities of individual large-ring cyclodextrins in complex mixtures. *Chem. Commun.* **2024**, *60*, 2090.
